# Caregivers’ and healthcare providers’ perceptions of blood availability, safety, and acceptability for patients with cancer in southwestern Uganda

**DOI:** 10.1371/journal.pgph.0007203

**Published:** 2026-09-11

**Authors:** Ivan Mugisha Taremwa, Nixon Niyonzima, Scholastic Ashaba, Cecilia Akatukwasa, Elizabeth Kemigisha, Deusdedit Tusubira, Carlrona Ayebazibwe, Shiller Atuhaire, May Y. Choi, Guido van Marle, Hume A. Heather, Bernard Natukunda

**Affiliations:** 1 Faculty of Medicine, Mbarara University of Science and Technology, Mbarara, Uganda; 2 Institute of Allied Health Sciences, Clarke International University, Kampala, Uganda; 3 Uganda Cancer Institute, Kampala, Uganda; 4 Infectious Diseases Research Collaboration, Kampala, Uganda; 5 African Population and Health Research Center, Nairobi, Kenya; 6 Department of Medicine, Cumming School of Medicine, University of Calgary, Alberta, Canada; 7 Snyder Institute for Chronic Diseases, University of Calgary, Alberta, Canada; 8 Department of Microbiology, Immunology and Infectious Diseases, Cumming School of Medicine, University of Calgary, Alberta, Canada; 9 Department of Pediatrics, University of Montreal, Quebec, Canada; PLOS: Public Library of Science, UNITED STATES OF AMERICA

## Abstract

Blood transfusion is crucial in supporting the treatment of patients with cancer. However, in poor-resource settings, blood and blood components remain scarce, and their perceived health risks may hinder their use. This study explored the perceptions of blood availability, safety, and acceptability among caregivers and healthcare providers of patients with cancer at Mbarara Regional Referral Hospital in southwestern Uganda. This study employed a qualitative design involving in-depth interviews and key informant interviews with caregivers of patients with cancer and healthcare providers, between September and December 2024. The interviews were audio-recorded, transcribed, translated, and analyzed using thematic content analysis. Of the 30 participants enrolled, 16 were caregivers of patients with cancer (mean age: 36; standard deviation, SD: 10.65 years) and 14 were healthcare providers (mean: 6.5; SD: 5.77 years of practice). Blood availability relied mostly on volunteer donors in educational institutions, and blood shortages were common during school recesses. Other highlighted additional constraints included donor complacency, limited availability of some blood groups, and disruptions during disease outbreaks. While most caregivers trusted that transfused blood was safe, some expressed concerns regarding adverse events and infection risks. Caregivers highly accepted blood transfusion as a life-saving intervention, but concerns relating it to end-of-life care, as well as cultural and religious beliefs were documented, and these were reported to influence transfusion acceptance. These findings reveal persistent blood shortages, with transfusion acceptance influenced by system-level and sociocultural constraints. Since patients with cancer require frequent transfusions, addressing challenges of blood supply and transfusion uptake through coordinated interventions is essential to support cancer care.

## Introduction

Cancer is the second leading cause of death globally [1], with an estimated 20 million new cases diagnosed in 2022, including over 2.6 million in Africa and about 36,000 in Uganda [2]. Patients with cancer frequently develop reduced blood cell counts, necessitating blood transfusions.

Blood transfusion, the administration of whole blood and its components, remains critical in supportive cancer care. In fact, 6% of all transfusions in sub-Saharan Africa (SSA) are given for cancer care [3], and blood and component therapy are now included on the World Health Organization (WHO) model list of essential medicines [4]. Of concern, frequent blood transfusions among patients with cancer are complicated by the documented clinical and perceived risks [5,6]. For instance, studies conducted in the United States and the United Kingdom reported that while patients and healthcare providers recognized transfusion as beneficial, there were concerns related to adverse events following transfusion, infection risk, and treatment burden [7–9]. Relatedly, caregivers of children with severe anemia in Uganda worried about Human Immunodeficiency Virus (HIV) infection and transfusion with incompatible blood [10]. In Saudi Arabia, concerns related to the transmission of HIV, Hepatitis B, and C were reported [11]. These perceived safety concerns as well as cultural and religious barriers may negate transfusion uptake [12]; thus compromising the care of patients who require frequent transfusion. However, evidence on caregivers’ and healthcare providers’ perceptions of blood transfusion in cancer care remains limited in Uganda. Understanding these perspectives is essential for identifying barriers to optimal transfusion care as well as informing strategies to strengthen transfusion services in cancer settings. This study aimed to explore caregivers’ and healthcare providers’ perceptions of blood availability, safety, and acceptability at Mbarara Regional Referral Hospital, southwestern Uganda.

## Materials and methods

### Study design, duration, and sites

This qualitative study used an instrumental case study approach, examining Mbarara Regional Blood Bank (MRBB) and Mbarara Regional Referral Hospital (MRRH) as an interconnected blood transfusion unit, from donor to recipient. The case was defined as the full transfusion pathway linking the two institutions, encompassing blood donation, processing, distribution, clinical decision-making, and transfusion, and was geographically and functionally bound to these sites. The case study framework informed purposive sampling across key points in the transfusion pathway, guided data collection across both institutions, and supported thematic analysis and interpretation of the findings within the bounded system. This approach allowed an in-depth, interpretive exploration of processes and experiences within an integrated blood transfusion system.

The study was conducted between September and December 2024. Both MRBB and MRRH are under the Ministry of Health, located in Mbarara City, approximately 270 kilometres southwest of Kampala, the Capital City of Uganda. MRRH is a tertiary healthcare facility serving more than 6 million people across 13 districts in southwestern Uganda and the neighboring districts of Rwanda, Tanzania, and the eastern Democratic Republic of Congo. MRBB is an establishment of Uganda Blood Transfusion Services (UBTS), mandated with the collection, processing, and supply of safe blood and components in the southwestern region.

### Study participants and size

The study enrolled caregivers of patients with cancer at MRRH, as well as selected healthcare providers at both MRRH and MRBB. Caregivers of patients with cancer were recruited as proxies for the in-depth interviews (IDIs) rather than patients themselves because many patients with cancer requiring transfusion are clinically unstable and psychologically vulnerable [13], making additional research participation potentially burdensome and ethically challenging. Furthermore, as the transfusion processes in this setting are largely family-mediated, with caregivers playing a key role in consent and transfusion decision-making [14], caregivers’ perspectives may influence transfusion uptake. Therefore, given that the study aimed to explore the perceptions of transfusion acceptance and access rather than individual illness experience, caregivers were considered for IDIs.

The sample size was guided by the principle of data saturation [15,16]. Thus, 30 interviews were deemed appropriate as responses became repetitive and no new insights were identified. Of these, 16 IDIs were conducted with caregivers of patients with cancer, sampled purposively from both the pediatric and adult cancer wards. Eleven IDIs were conducted with healthcare providers, including six nurses, three laboratory personnel, and two medical officers. Additionally, three key informant interviews (KIIs) were carried out, comprising two ward in-charges at MRRH, and one quality assurance focal person from UBTS. Participants were selected based on their roles in blood transfusion. Nurses at the blood bank are responsible for donor eligibility assessment and blood collection, while hospital-based nurses provide patient care and oversee transfusion administration. Medical officers assess patients’ need for transfusion and make the transfusion orders. Laboratory personnel at the blood bank screen donor units for transfusion-transmissible infections (TTIs) and issue safe blood units, whereas hospital-based laboratory staff conduct pre-transfusion testing and participate in the investigations of any transfusion reactions. The UBTS quality assurance focal person oversees quality assurance processes and training support. By selecting participants from diverse groups (Table 1), we aimed to generate comprehensive and representative responses.

**Table 1:** Characteristics of participants.

Interview type	Description	Number of interviewees per site (category by gender)	
		MRRH	MRBB
In-depth Interviews	Adult caregivers (parents, spouse, or adult sons/daughters) of patients with cancer	16 (11 Females, 5 Males	Not Applicable
	Healthcare providers	8 (3 Females, 5 Males)	3 (1 Female, 2 Males)
Key Informant Interviews	Health care providers with the oversight responsibility	2 (1 Female, 1 Male)	1 Male
Total		26 (15 Females, 11 Males)	4 (1 Female, 3 Males)

### Participant enrollment

The treating medical officer identified the eligible patients with a cancer diagnosis who had previously received blood transfusions. For adult patients, the medical officer informed them about a study seeking to recruit their caregivers and obtained verbal consent to disclose the patient’s diagnosis and transfusion history to the research team for purposes of the caregiver recruitment. For pediatric patients, consent was obtained from the parent or legally authorized guardian, and assent was obtained from the stable child if the age was ≥ 8 in accordance with approved ethical procedures. This verbal consent or assent was documented in the recruitment study log, and the procedure was approved by the Mbarara University of Science and Technology Research Ethics Committee. Following permission, a designated member of the research team met with the patient and their caregiver in privacy, provided detailed study information, assessed caregiver eligibility, obtained written informed consent from the caregiver, and then scheduled the interview.

### Inclusion and exclusion criteria

Caregivers were eligible if they were aged ≥ 18 years, had provided care to a patient with cancer for at least 1 month, were aware that the patient had been diagnosed with cancer, and were caring for a patient who had received at least two blood transfusions in the past 3 months, including a transfusion during the current hospitalization as part of their clinical care. The 1 month caregiving period was considered sufficient to ensure familiarity with the patient’s ongoing care needs and recent transfusion experiences. Healthcare providers at MRRH were eligible if they had worked in the cancer unit for at least 3 months and had prescribed (for medical officers), administered blood (for nurses), or issued blood (for laboratory staff). Healthcare providers at MRBB were eligible if they had worked in their roles for at least 3 months. Key informants were considered if they had prescribed blood transfusions for patients with cancer or were involved in regional training for MRBB. Caregivers were excluded if the patient required continuous bedside monitoring and no temporary cover was available to allow participation in a private interview. We excluded caregivers with mental instability and those whose patient died before the interview could be conducted. Healthcare providers who were on annual leave during the study period were not eligible for inclusion.

### Sampling procedure

Purposive sampling was used to recruit caregivers and healthcare providers who met the inclusion criteria, ensuring inclusion of respondents with relevant experience. Key informants were recruited using convenience sampling due to their specialized roles.

### Data collection tools and approach

Data were collected using semi-structured in-depth interview and key informant interview (KII) topic guides developed in English from existing literature [7,9,11,17]. The topic guides included open-ended questions to explore the perceptions of blood needs, availability of blood, safety concerns, acceptability, and alternatives to blood transfusion (S1 Appendix). IDIs with caregivers were conducted in participants’ preferred commonly spoken local languages (Runyankore-Rukiiga, Runyoro-Rutoro, Rufumbiira, and Luganda) by trained graduate research assistants fluent in these languages. The interview guides were developed in English and administered through interviewer translation during the interviews. To maintain consistency across languages, the interviewers received training on the intent and meaning of each question and used the agreed-upon terminology for key concepts. On the other hand, interviews with healthcare providers were conducted in English. Each interview had a facilitator and a note-taker, who also managed audio recordings. Interviews were conducted in private rooms at the pediatric and adult wards at MRRH. Healthcare providers were interviewed at their convenience at their workplaces. Interviews lasted about 40–60 minutes and were audio-recorded. Probing questions were used to clarify responses and explore emerging issues, allowing for an iterative approach to data collection. Interviews continued until thematic saturation was reached [18], and detailed field notes were taken. One of the authors (IMT) transcribed all interviews and verified transcript accuracy, as well as the preservation of meaning. Interviews conducted in local languages were independently translated into English by two other authors (AC and AS), and IMT reviewed translations to ensure fidelity and resolved discrepancies.

To protect the participants confidentiality, audio recordings and transcripts were assigned unique study identification codes and did not contain participant names or any direct identifiers. Any potentially identifying information mentioned during the interview process (such as individuals and organizations) was removed from the transcription. More, only the authorized members of the research team had access to the audio recordings and transcripts, and de-identified transcripts were used for translation, transcription, coding as well as analysis.

### Quality assurance

The interview guides were pilot-tested at MRRH to assess content validity and comprehension. These were then refined to improve clarity. The topic guides were reviewed by experts in blood transfusion, cancer, and qualitative research. To minimize social desirability bias, interviewers used open-ended questions, probing techniques, and careful framing of sensitive questions.

Transcription accuracy was ensured by comparing transcripts with the audio recordings.

### Data management and analysis

Descriptive statistics were used to summarize the participants’ demographic characteristics. Qualitative data were analyzed using an inductive, team based thematic approach incorporating codebook development and iterative consensus coding. While the analysis was informed by the study objectives, no predetermined coding framework was applied. Instead, codes were generated from the data through repeated reading of transcripts and identification of concepts expressed by the participants. Three individuals (IMT, CA, and AS) independently reviewed and coded the transcripts. An initial codebook was developed using conventional content analysis after the coders independently reviewed the first six transcripts and was iteratively refined as new codes and themes emerged during analysis.

The coding process involved a line-by-line review of each transcript to identify patterns, relationships, and emerging themes. To enhance analytic rigor and minimize bias, three members of the research team (IMT, CA, and AS) independently coded transcripts and compared interpretations through regular consensus meetings. Discrepancies were resolved through discussion and re-reading the relevant sections of the transcripts together, and agreed on the best-fit interpretation of the data. Further analysis involved grouping similar codes to develop themes, which were refined through iterative team discussion.

All transcripts were uploaded into NVivo Software [19], checked for accuracy, and systematically analyzed. A structured 6-step thematic analysis process was used to guide the analytical process, including data familiarization, initial code generation, code refinement and theme development, consensus-based theme review, theme naming and definition, and synthesis of findings [20]. In reporting the findings, qualitative frequency descriptions such as “most participants,” “some participants,” and “a few participants” were used to indicate the relative prevalence of views across interviews without implying statistical quantification.

### Ethical considerations

The study obtained ethics approval from the Mbarara University of Science and Technology Research and Ethics Committee (MUST-2024–505) and the Uganda National Council for Science and Technology (HS4551ES). Written informed consent was obtained prior to study activities in which participants were informed about their right to decline or withdraw from the study and the confidentiality of their responses. Participants consented to the audio recording of the interviews and to the use of anonymized data and quotations in research reports and publications. Interviews were carried out in private rooms, and the audio recordings were transferred to a password-protected computer that was restricted to the authorized members of the study team. As caring for patients with cancer is associated with psychosocial distress, participants requiring emotional or psychological support were referred to the psychiatry unit at MRRH for psychosocial support services.

## Results

### Study participants characteristics

Of the 30 participants enrolled, 16 were caregivers of patients with cancer (mean age: 36; SD: 10.65 years), and 14 were healthcare providers (mean: 6.5; SD: 5.77 years of practice) (Table 2).

**Table 2:** Demographic characteristics of participants.

Variable	N (%)
*Caregivers of patients with cancer (N = 16)*	
**Age category (Years)**	
< 35	9 (56.2%)
35-65	7 (43.8%)
**Sex**	
Female	11 (68.7%)
Male	5 (31.3%)
**Relationship with the patient**	
Child	4 (25.0%)
Parent	8 (50.0%)
Spouse	4 (25.0%)
**Marital status**	
Single	1 (6.3%)
Married	13 (81.2%)
Others (divorced, widowed)	2 (12.5%)
**Level of education**	
No formal education	1 (6.3%)
Primary	7 (43.7%)
Secondary level	6 (37.5%)
Tertiary	2 (12.5%)
**Religion**	
Protestant	8 (50.0%)
Catholic	4 (25.0%)
Muslim	2 (12.5%)
Pentecostal	2 (12.5%)
*Healthcare providers (N = 14)*	
**Duration of healthcare service (Years)**	
≤ 5	7 (50.0%)
6-10	5 (35.7%)
> 10	2 (14.3%)
**Sex**	
Female	5 (35.7%)
Male	9 (64.3%)
**Healthcare role**	
Medical Officer	4 (28.6%)
Nurse	6 (42.8%)
Laboratory staff	4 (28.6%)

### Qualitative findings: Themes

Qualitative analysis of interviews with the caregivers of patients with cancer and healthcare providers identified three overarching themes relating to blood transfusion in the study setting: perceptions of blood availability, perceptions of blood safety, and acceptability of blood transfusion. These themes captured participants’ experiences and perceptions regarding access to blood and blood components, confidence in the transfusion safety, and factors influencing the acceptance or refusal of blood transfusion. More, each theme comprised several sub-themes that reflected the complexity of blood transfusion practices within the studied setting. Illustrative quotations are presented to support the findings.

### Theme 1: Perceptions of blood availability

#### Sub-theme 1: Perceived barriers to blood supply.

***Dependence on school-based blood donation cycles:*** Healthcare providers from UBTS stated that most of the blood donors were students in educational institutions and thus they perceived the school calendar to significantly influence blood collection, with donations reportedly peaking during the school term and declining during the school recess;

*“Over 80% of all the donated blood comes from school-going donors, and the availability of blood depends on school time...during the school term, we collect ≥100 units, but this drops to <20 units during the holidays.” (IDI, Healthcare Provider 7, Male)*

***Scarcity of certain blood groups:*** Healthcare providers reported a general scarcity of certain blood groups;

*“There is a scarcity of O and RhD-negative donor groups. …this frustrates patients and their caregivers, who may resort to alternatives.” (IDI, Healthcare Provider 1, Male)*

MRBB indicated challenges in mobilizing RhD-negative donors, noting that most of them were perceived to reside outside the region and would require transportation support to donate.

***Incentive expectations undermining altruistic blood donation:*** A healthcare provider from UBTS reported that some donors expected material incentives or benefits in exchange for donating blood, including tangible rewards such as bicycles or assurances of free medical services, as expressed;

*“Some donors believe in entitlements and rewards like bicycles...others demand assurance of completely free medical services if they fell sick.” (IDI, Healthcare Provider 4, Female)*

***Barriers to adult participation in blood donation:*** A healthcare provider from UBTS perceived adult participation in blood donation to be low due to daily life commitments. Some of the healthcare providers also expressed negative sentiments regarding adults’ willingness to donate blood, as quoted;

*“Some adults believe that blood donation is the responsibility of a specific group of people. …they express sentiments like, ‘why should I donate? I won’t be the one needing the blood anyway.” (IDI, Healthcare Provider 4, Female)*

***Impact of adverse weather on donor turnout and blood collections:*** Healthcare providers from UBTS indicated that adverse weather negatively affected donor turnout during field collections. They reported that cold conditions made phlebotomy more challenging and were occasionally associated with failed donation attempts.

***Fear during disease outbreaks limiting blood collection:*** Healthcare providers from UBTS recognized that disease outbreaks, such as Ebola, appeared to reduce communities’ willingness to donate blood. They viewed community members as being less willing to engage in donation due to fear and self-isolation, as reported;

*“During an outbreak, blood collections decline as field engagements are reduced...people self-isolate; reducing blood supply.” (IDI, Healthcare Provider 2, Male)*

#### Sub-theme 2: Perceived barriers to voluntary blood donation.

Some caregivers and healthcare providers expressed concerns that acted as barriers to voluntary blood donation. They reported that communities sometimes held negative attitudes toward the donation process, partly influenced by a perceived lack of reciprocity between blood donation and access to transfusion services. While blood donation is voluntary and non-remunerated, caregivers and healthcare providers reported that concerns about transfusion-related expenses incurred in healthcare facilities contributed to apprehension about donating blood, as expressed;

***Caregiver’s report*** *: “Blood transfusion isn’t for free! In private facilities, costs related to transfusion are included in a patient’s medical bill...in government healthcare facilities, you offer ‘a token of facilitation’..” (IDI, Caregiver 16, 32 Years, Male)*

***Healthcare provider report*** *: “In the communities, people are generally willing to donate blood. However, a common concern raised to us is: ‘why do patients have to pay for a transfusion when we donate blood for free?” (IDI, Healthcare Provider 4, Female)*

#### Sub-theme 3: Perceived turnaround time and operational constraints affecting blood component preparation.

***Field collections as a barrier to the preparation of components:*** Healthcare providers from UBTS reported that the majority of blood donations were collected in the field, and they noted that delays in transporting blood could sometimes limit its timely availability for component preparations;

*“The blood collection teams travel to distant locations, often spending the entire day in the field. By the time the blood reaches the blood bank, it is no longer within the required time frame for immediate use or for preparing platelets or fresh frozen plasma.” (IDI, Healthcare Provider 2, Male)*

***Centrifuge breakdown and prolonged equipment downtime affecting component preparation:*** Healthcare providers perceived the breakdown of the only centrifuge used for platelet preparation as a major factor compromising the timely and adequate availability of platelets. The impact was worsened by prolonged equipment downtime due to the absence of resident servicing personnel.

#### Sub-theme 4: Perceived chronic scarcity of blood and its clinical consequences.

***Increasing demand vis-à-vis limited supplies:*** Healthcare providers described blood as a scarce and highly needed resource, with demands extending beyond cancer care. They noted that in situations of limited blood supply, prioritization often favored ‘emergency cases’ as described;

*“During times of blood scarcity, there are prioritization decisions. Whenever a transfusion is requested for a patient with cancer and patients from other wards, the priority is the patient whose condition is more likely to be immediately stabilized by the transfusion...unfortunately, patients with cancer are often the least prioritized.”* (*IDI, Healthcare Provider 3, Female)*

***Missed blood transfusions due to shortages:*** Most caregivers reported that their patients had missed transfusions due to blood shortages. They noted that this resulted in the rescheduling of medical appointments for surgeries and chemotherapy. Caregivers attributed some deaths to missed transfusions.

Overall, blood was perceived as insufficient. Healthcare providers perceived the system as structurally fragile that was overly dependent on school-going donors, whose contributions sustained the blood availability during school terms but created severe shortages during recess periods. Also, the scarcity of blood group O and all RhD-negative donors was reported. Moreover, community disengagement from donation due to competing priorities and negative attitudes mostly among adults emerged. Of concern, healthcare providers from UBTS reported that some donors expected material incentives and assurance of medical benefits. This approach was perceived to conflict with the principle of voluntary, non-remunerated donation. Moreover, transfusion-related charges were perceived to contradict the altruistic basis. Additionally, public health emergencies like disease outbreaks were reported to disrupt field activities. Notably, distant field operations and the breakdown of the centrifuge were also reported to constrain the preparation and availability of components. These factors were considered to contribute to chronic scarcity and rationing of the available blood, often prioritizing acute emergencies over patients with cancer, resulting in missed transfusions, treatment delays, and deaths that caregivers perceived as preventable.

### Theme 2: Perceptions of blood safety

#### Sub-theme 1: Institutional trust in screening and laboratory safeguards.

Healthcare providers and most caregivers expressed confidence in the safety of donor blood due to the rigorous pre-donation screening and laboratory testing protocols for TTIs, as quoted;

***Caregiver report:*** *“I trust the safety of this blood...the blood bank team assured us that all donated units are tested, and blood units are only transfused if found free of infections...units found to be infected are discarded.” (IDI, Caregiver 11, 53 Years, Female)*

***Healthcare Provider report:*** *“The donor blood is safe. The Uganda Blood Transfusion Services use the best technology to screen the blood.” (IDI, Healthcare Provider 2, Male)*

#### Sub-theme 2: Perceived limitation of donor screening and residual risk of TTIs.

***High-risk donors:*** A key informant from UBTS noted that some walk-in donors were primarily motivated by access to diagnostic testing for HIV and other infections. This perception raised concerns regarding the reliability of self-reported risk histories collected during donor assessment, as quoted;

*“Some of the walk-in donors, including sex workers, come to donate blood with the intention of testing for HIV and other infections.” (KII, Healthcare Provider 1, Male)*

***Residual risk of TTIs:*** A few caregivers expressed worry about the limitations of laboratory testing, noting that the residual risk of TTI raised fears of possible transfusion-transmission, as quoted;

*“Early-stage infections may be missed during blood testing, risking transmission of HIV, hepatitis, or syphilis...a relative contracted HIV possibly through multiple childhood transfusions.” (IDI, Caregiver 10, 20 Years, Female)*

In some instances, caregivers’ prior experiences with post-transfusion illness appeared to reinforce concerns about transfusion safety, even when a direct causal link was uncertain. One caregiver expressed concern based on prior experiences with bacterial infections, leading to skepticism about whether or not the transfusions were the cause, as expressed;

*“After a blood transfusion, the patient got ill with a diagnosis of a bacterial infection. One cannot rule out whether it was through the transfusion.*” *(IDI, Caregiver 1, 43 Years, Female)*

#### Sub-theme 3: Concerns about adverse transfusion reactions and errors.

A few caregivers described apprehension arising from lived experiences of transfusion reactions and suspected laboratory error, which shaped their perceptions of transfusion safety as reported;

*“The patient started scratching her body. I (the caregiver) thought that the ‘new blood’ was mixing with the one it had found in the body.” (IDI, Caregiver 1, 43 Years, Female)*

*“The child was transfused with the wrong blood group, which caused the body to swell...I worry that such an error can kill a patient!” (IDI, Caregiver 9, 42 Years, Male)*

#### Sub-theme 4: Misbeliefs/Misconceptions.

Because of adverse effects and the prevailing community misconceptions, a few caregivers expressed concerns and believed that donor blood was from non-human sources, as narrated;

*“After transfusion, the patient often becomes restless. I (the caregiver) sometimes wonder where the blood comes from!..other caregivers ask which animal did this blood come from?” (IDI, Caregiver 4, 25 Years, Female)*

Generally, the perceptions of blood safety were shaped by institutional trust, personal experience, and socio-cultural beliefs. Most participants expressed confidence in the safety of donor blood based on the rigorous pre-donation screening and laboratory testing protocols. However, this trust was undermined by the perception that high-risk walk-in donors seek diagnostic testing, prior experiences with adverse transfusion events and purported transmission of TTIs. Thus, trust in procedural safety coexisted with caution. In addition, sociocultural narratives and misconceptions influenced the perceptions of donor blood.

### Theme 3: Acceptability of blood transfusion

#### Sub-theme 1: Enablers of blood transfusion uptake.

***Perceived benefit of restoring life and the patient’s well-being:*** Healthcare providers and most caregivers viewed blood transfusions as highly beneficial in the care and management of patients with cancer. Most caregivers trusted that healthcare providers administered treatments, including transfusions, with the intent to heal. Transfusions helped alleviate symptoms such as fatigue, pallor, restlessness, and palpitations. A caregiver highlighted the life-impacting benefits of blood transfusion, as quoted;

*“If he (the patient) hadn’t been transfused, he likely wouldn’t be alive...whenever he doesn’t receive a transfusion, he feels unhappy and dissatisfied with the treatment, often complaining that he has missed out on a lot.” (IDI, Caregiver 7, 25 Years, Male)*

Most caregivers expressed deep gratitude for the health improvements observed following transfusions, describing them as providing “a second chance at life” that restored energy and daily functioning. Healthcare providers similarly perceived blood transfusions as indispensable in clinical care, particularly for patients with cancer and those requiring palliative support. They highlighted that transfusions were essential for controlling bleeding, improving blood cell parameters, and sustaining physiological stability, which in turn enabled other therapeutic interventions.

***Life-saving impact as a driver of transfusion acceptance:*** A caregiver reported that the patient’s recovery from a near-death condition was clear evidence that blood transfusion saves lives and expressed willingness to accept transfusions, as reported;

*“I (the caregiver) have witnessed how much better the patient feels after a transfusion. I support it (blood transfusion), and would never refuse it*.” *(IDI, Caregiver 1, 43 Years, Female)*

***Failure of the alternative remedies:*** A few caregivers stated transitioning from alternative remedies to formal healthcare, choosing to accept blood transfusions for their patients, as other remedies, such as herbal treatments, did not improve the patients’ health conditions, as stated;

*“After saucepans of herbs, he (the patient) didn’t improve...I (the caregiver) took him to the hospital and accepted a blood transfusion.”* (*IDI, Caregiver 15, 48 Years, Male)*

#### Sub-theme 2: Fears and misconceptions about blood transfusion.

***Religious and cultural beliefs:*** Caregivers belonging to the Jehovah’s Witness denomination and some patients from the Kasese region and of Somali origin were reported to decline blood transfusions. Healthcare providers indicated that the Jehovah’s Witnesses involved their pastoral leaders, who strongly condemned blood transfusion, arguing that ‘it is forbidden and sacred’. To them, ‘blood symbolizes life, and God has reserved its use for His divine authority.’ Patients and caregivers from Somalia and Kasese were reported to cite cultural beliefs and fears that transfused blood could transfer undesirable traits from the donor, as narrated;

***Jehovah’s Witnesses’ religious perspective on blood transfusion*** *: “Blood is sacred and represents life...only God has authority over it, and using it outside His will is forbidden...accepting transfusion is a violation of divine law.” (KII, Healthcare Provider 2, Female)*

***Cultural perceptions of blood transfusion*** *: “I (caregiver) fear that if he (the patient) receives blood from another person, he will acquire their (blood donors’) traits and illnesses...in our culture (Bakonzo), blood is more than just life. It is an identity and essence...we can not take that risk.” (IDI, Caregiver 16, 32 Years, Male)*

*“In our community (Somali), receiving blood touches our identity and family lineage...the foreign blood can bring unwanted behaviors!” (IDI, Caregiver 6, 60 Years, Female)*

***Fear associated with blood transfusion:*** A few caregivers related blood transfusions to very sick and near-death patients, as quoted;

*“I (the caregiver) grew up believing that when a patient reaches the stage of needing a transfusion, it often means they are about to die!..the patient was too weak, and I feared that if she got a transfusion, she might die!” (IDI, Caregiver 4, 25 Years, Female)*

#### Sub-theme 3: Complementary and supportive therapeutic practices alongside blood transfusion.

***Drugs:*** Healthcare providers perceived patient management as multifaceted and extending beyond blood transfusion. They reportedly administered tranexamic acid, prophylactic anticoagulants, hematinics, vitamin supplementation, folic acid, and iron. These medications were prescribed as part of a comprehensive therapeutic approach aimed at controlling bleeding, preventing thromboembolic complications, and correcting the underlying defects.

***Complementary and traditional approaches:*** Most caregivers indicated supplementing the care of patients with cancer with fruits and vegetables, such as mangoes, passion fruits, watermelons, hibiscus, beetroots, and a vegetable locally known as ‘*buga*’. Additionally, patients with cancer consumed foods like millet, liver, fresh blood from slaughtered animals (like cows, goats, sheep), and others used herbal concoctions comprising the leaves of avocado, pawpaw, jackfruit, and mango, among others. These remedies were viewed as supportive therapies intended to replenish blood, enhance the patient’s overall health, and aid in recovery. A caregiver reportedly gave a herbal concoction to a patient following a blood transfusion, as quoted;

*“By the time the healthcare provider tells you that the patient needs a blood transfusion, the patient is critically ill...you accept the transfusion, but you also provide herbs to add to that blood!” (IDI, Caregiver 6, 60 Year, Female)*

Collectively, the acceptability of blood transfusion was shaped by therapeutic benefits, lived experiences of clinical improvement, trust, sociocultural and religious beliefs, and recourse to complementary and supportive therapies. On the contrary, blood transfusion was simultaneously constrained by deeply rooted religious and cultural beliefs where blood was not merely regarded as a biological fluid but as an embodiment of identity, lineage, essence, and moral attributes generating concerns that the transfused blood could transmit undesirable traits, illnesses, or spiritual qualities. Additional hesitation emerged from the perception that transfusion signifies impending death or extreme disease severity. Due to the barriers, alternatives to transfusion were opted for and sometimes were reportedly co-administered, illustrating medical pluralism.

## Discussion

This study explored the perceptions of blood availability, safety, and acceptability among caregivers and healthcare providers of patients with cancer at Mbarara Regional Referral Hospital in southwestern Uganda. From this study, the availability of blood was affected by school holidays, donor complacency, high demand, scarcity of O and RhD-negative blood groups, rainy seasons, and disease outbreaks. Caregivers generally viewed blood as safe amidst concerns of adverse events and infection risks. Blood transfusions were highly valued; however, cultural and religious beliefs hindered their acceptability. Thus, supportive medical therapies and complementary practices were used alongside blood transfusion, and in some instances when transfusion was declined or unavailable. Healthcare providers perceived the blood supply as fragile and largely reliant on institution-based donors, and it was characterized by marked seasonal fluctuations during school terms and recesses.

Overall, while both caregivers of patients with cancer and healthcare providers identified similar structural constraints affecting blood availability, differences were observed in how these challenges were framed. Caregivers of patients with cancer tended to emphasize cultural, religious and experiential concerns related to transfusions, whereas the healthcare providers more frequently highlighted systemic and supply-chain limitations within the blood transfusion system.

This perception is supported by data from UBTS: in 2022–2023, only 313,659 units were collected, satisfying approximately 70% of the country’s requirements [21] and falling short of the WHO’s recommended minimum donation level of 1% of the population annually [22]. The high dependency on educational institution-based donors reflects organized campaigns targeting students, a group perceived as a lower-risk population for TTIs [23], and aligns with patterns documented in low- and middle-income countries. In contrast, adult participation in voluntary blood donation was viewed as lower, mostly attributed to their competing socio-economic demands and, as some healthcare providers reported, limited motivation or engagement with blood donation initiatives. Additional challenges of blood availability were compounded by the perceived scarcity of O and RhD-negative blood groups, possibly due to a low donor-to-recipient ratio. As reported by previous studies [24–26], factors such as donor complacency and disease outbreaks remain a hindrance to blood collection. The above factors collectively were perceived to result in the scarcity of blood, undermining the healthcare provision by prolonging the waiting times and hospital stays. Moreover, the reports of ‘token of facilitation’ in the attempt to secure blood for the patients contravene the basis of altruism in blood banking. Further, private health facilities reportedly included ‘blood transfusion-related costs’ in their medical bills, likely reflecting charges for the transfusion services themselves rather than the cost of the blood unit. These reported practices were viewed to create a perception of commodification of blood and likely discourage sustainable participation by altruistically motivated donors, as previously reported [27]. Further research is needed to better understand the nature, extent, and implications of these reported practices and to inform appropriate policy and regulatory responses. Given the challenges posed by the insufficiency of blood, there is a need for public awareness to address the persistent suboptimal annual blood collection in Uganda. More, although not explored in this study, avoidable transfusions create an imbalance between blood supply and demand, leading to unavailability for patients with critical needs [28]. This study adds to the advocacy for the proper utilization of blood as a scarce and expensive resource.

Caregivers of patients with cancer perceived blood safety with ‘mixed reactions’. Although blood was highly regarded as safe, concerns about the associated risks emerged. These perceptions reflect awareness of potential risks, even in systems that adhere to WHO-recommended safety standards. The safety attributes are evidence of the rigorous pre-donation screening, laboratory testing, and quality assurance protocols, aligning perceptions with reality and reinforcing trust in the blood supply. On the other hand, caregivers expressed worries about infections and adverse events, similar to previous reports [7,9–11,17]. The perceived safety concerns underscore the challenges caregivers may face in balancing the perceived risks of blood transfusion with its life-saving benefits [11].

Blood transfusion was generally acceptable; however, underlying expression of discontent revealed safety and appropriateness concerns. The enormous acceptance correlates with the existing data on the indispensable life-saving role of blood transfusion. For instance, in the United States, blood transfusion was regarded as “critical” and “of greatest importance” [29], similar to the findings in a study conducted at Ohio Hospital, where the benefits of blood transfusion were difficult to discern due to the patient’s disease condition [30]. Notably, cultural and religious contempt of blood use was also reported, similar to previous studies [10,11,31], and may negatively influence transfusion uptake. Due to the limited availability, safety concerns, and objections to blood, participants reportedly used a range of complementary and traditional therapies. The role, effectiveness, and safety of these complementary and traditional practices remain complex and require further research to safeguard patients’ health.

While blood transfusion is generally perceived as acceptable and life-saving, various perceptions identified in this study may undermine both its perceived safety and acceptability. For instance, some caregivers viewed transfusion as interventions reserved for the ‘very sick patients and as a last resort’, which may explain the misbelief that transfused patients were likely to die. This perception is likely shaped by the clinical context in which transfusions are frequently administered after alternative treatments have failed or when the patient’s condition is severe. Thus, associating transfusion symbolically with impending death rather than recovery. Moreover, delayed consent or hesitation in such cases can compromise timely management of severe anemia or hemorrhagic shock, thereby increasing the risk of mortality. Therefore, the perceived association between transfusion and death likely reflects temporal proximity rather than causation, and hemovigilance data indicate that transfusion-attributable mortality is rare and usually linked to identifiable adverse events [32,33]. Further, caregivers reported concerns about donors in the window period, during which infections may not be detectable, contributing to fears of TTIs. Healthcare providers also perceived risk from ‘walk-in’ donors seeking infectious disease testing through donation, while caregivers reported anecdotes of individuals testing HIV-negative before transfusion but positive afterwards. Although TTIs remain theoretically possible, rigorous donor screening has markedly reduced the risk. Additionally, the perceptions that transfused blood might originate from non-human sources or the transfer of a donor’s trait are incorrect. These perceptions suggest that blood may be understood not only as a biological substance but also as a carrier of identity, personal attributes, and social meaning. The findings highlight the importance of culturally sensitive educational and counselling interventions that address transfusion-related concerns while acknowledging the social and symbolic meanings attached to blood. Finally, although participants reported the use of animal blood or herbal remedies to support recovery, the role, effectiveness and safety of such practices in the management of severe or chemotherapy-induced anemia remain insufficiently understood and warrant further investigation. Of concern, in already resource-constrained settings, such unsupported perceptions may shape how patients and caregivers view blood transfusion and could influence transfusion-related decision-making. Addressing these concerns through culturally sensitive communication may help support informed decisions regarding transfusion within the broader context of the existing blood system challenges. The strengths of this study are highlighted by its dual focus on transfusion perceptions from both patients’ caregivers and healthcare providers in a context that has not been studied before in SSA. On the other hand, the study findings ought to be interpreted in light of the following: its single-center design and the fact that its results may be skewed towards the studied categories, excluding blood donors. Despite these limitations, the findings offer critical insights into the caregivers’ perception on blood availability, safety, and acceptability, which will guide future research.

## Conclusions

This study reports divergent perceptions on blood availability, safety, and acceptability at a tertiary healthcare facility. The perceptions of blood availability reflected systemic challenges, including seasonal shortages, donor complacency, scarcity of certain blood groups, and increased clinical demand. Blood safety remains a worry due to the perceived risk of infections and adverse events. Also, blood acceptability was subjective, mainly affected by religious, cultural, and experiential misconceptions. Notably, some perceptions that are not supported by evidence, such as beliefs in non-human sources of blood, transfer of personality, and inherent cause of death reflect the social and symbolic meanings attached to blood and may influence how patients and caregivers approach transfusion-related decision making.

Given that patients with cancer often require blood transfusions, efforts to ensure availability, safety, and improve acceptability are critical. Additionally, addressing both evidence-based and misinformed perceptions is essential for ensuring timely and appropriate use of blood. Thus, multipronged approaches such as information, education, and communication campaigns may help improve understanding of blood donation and improve the acceptability of blood transfusion.

## Supporting information

S1 Appendix Data collection tool. (DOCX)

S1 Data Supporting Information file with minimal dataset. (DOCX)
