# Supplementary material for: Caregivers’ and healthcare providers’ perceptions of blood availability, safety, and acceptability for patients with cancer in southwestern Uganda
Source: PLOS Glob Public Health. 2026 Sep 11;6(9):e0007203. doi: 10.1371/journal.pgph.0007203 (PMC13567789; doi:10.1371/journal.pgph.0007203)
Supplement: Supplementary file 1 [file pgph.0007203.s003.pdf]

PLOS Global Public Health

Caregivers’ and healthcare providers’ perceptions of blood availability, safety, and acceptability for patients with cancer in southwestern Uganda.

--Manuscript Draft--

**Manuscript Number:**	PGPH-D-26-01258
**Article Type:**	Research Article
**Full Title:**	Caregivers’ and healthcare providers’ perceptions of blood availability, safety, and acceptability for patients with cancer in southwestern Uganda.
**Short Title:**	Perceptions of blood use in Uganda
**Corresponding Author:**	Ivan Mugisha Taremwa, MSc<br>Mbarara University of Science and Technology Faculty of Medicine<br>Kampala, UGANDA
**Order of Authors:**	Ivan Mugisha Taremwa, MSc
	Nixon Niyonzima, PhD
	Scholastic Ashaba, PhD
	Cecilia Akatukwasa, PhD
	Elizabeth Kemigisha, PhD
	Deusdedit Tusubira, PhD
	Carlrona Ayebazibwe, BSc
	Shiller Atuhaire, BSc
	May Y. Choi, MSc
	Guido van Marle, PhD
	Hume A. Heather, PhD
	Bernard Natukunda, PhD
**Keywords:**	Caregivers, healthcare providers, perceptions, blood use, availability, safety, acceptability, qualitative research, Uganda
**Abstract:**	Blood transfusion is crucial in supporting the treatment of patients with cancer. However, in poor-resource settings, blood and blood components remain scarce, and their perceived health risks may hinder their use. This study explored the perceptions of blood availability, safety, and acceptability among caregivers and healthcare providers of patients with cancer at Mbarara Regional Referral Hospital in southwestern Uganda.<br>This study employed a qualitative design involving in-depth interviews and key informant interviews with caregivers of patients with cancer and healthcare providers, between September and December 2024. The interviews were audio-recorded, transcribed, translated, and analyzed using thematic content analysis.<br>Of the 30 participants enrolled, 16 were caregivers of patients with cancer (mean age: 36; standard deviation, SD: 10.65 years) and 14 were healthcare providers (mean: 6.5; SD: 5.77 years of practice). Blood availability relied mostly on volunteer donors in educational institutions, and blood shortages were common during school recesses. Other highlighted additional constraints included donor complacency, limited availability of some blood groups, and disruptions during disease outbreaks. While most caregivers trusted that transfused blood was safe, some expressed concerns regarding adverse events and infection risks. Caregivers highly accepted blood transfusion as a life-saving intervention, but concerns relating it to end-of-life care, as well as cultural and religious beliefs were documented, and these were reported to influence transfusion acceptance. These findings show that caregivers experienced persistent shortages in blood availability in Uganda, while transfusion acceptance for patients with cancer was driven by system-level and sociocultural constraints. Since patients with cancer require frequent transfusions, addressing challenges of blood

Powered by Editorial Manager® and ProduXion Manager® from Aries Systems Corporation

	supply and transfusion uptake through coordinated interventions is essential to support cancer care.
Additional Information:	
Question	Response
**Financial Disclosure**<br><br>Enter a financial disclosure statement that describes the sources of funding for the work included in this submission and the role the funder(s) played. This includes grants and any commercial funding of the work or authors.<br><br>This statement will be typeset if the manuscript is accepted for publication.<br><br>*Please review the submission guidelines and the instructions link below for detailed requirements and guidance.*	This research was funded by the Government of Uganda through Mbarara University of Science and Technology, and MicroResearch Canada (24J_MUS_01). The funders did not influence the design, conduct, data acquisition, analysis, interpretation of the results, or the decision to publish
**Competing Interests**<br><br>On behalf of all authors, disclose any competing interests that could be perceived to bias this work.<br><br>This statement will be typeset if the manuscript is accepted for publication.<br><br>*Please review the instructions link below and PLOS Global Public Health's competing interests policy to determine what information must be disclosed at submission.*	None to declare
**Data Availability**<br><br>Before publication, Authors are required to make fully available and without restriction all data underlying their findings. Please see our PLOS Data Policy page for detailed information on this policy.	The relevant data used is available in the manuscript.

A **Data Availability Statement**, detailing where the data can be accessed, is required at first submission. Insert your Data Availability Statement in the box below.<br><br>Please see the data reporting section of our submission guidelines for instructions on what you need to include in your Data Availability Statement.<br><br>This statement will be typeset if the manuscript is accepted for publication.<br><br>*PLOS allows rare exemptions to address legal and ethical concerns. If you have legal or ethical restrictions, please use the box below to detail these in full sentences for the Journal team to consider.*	

Powered by Editorial Manager® and ProduXion Manager® from Aries Systems Corporation

Manuscript

Click here to access/download;Manuscript;Perceptions of blood in Uganda.docx

Caregivers' and healthcare providers' perceptions of blood availability, safety, and acceptability for patients with cancer in southwestern Uganda.

Author names and Affiliations

Ivan Mugisha Taremwa[1,2], Nixon Niyonzima[3], Scholastic Ashaba[1], Cecilia Akatukwasa[4], Elizabeth Kemigisha[1,5], Deusdedit Tusubira[1], Carlrona Ayebazibwe[2], Shiller Atuhaire[1], May Y. Choi[6,7], Guido van Marle[7,8], Hume A. Heather[9], Bernard Natukunda[1]

[1]Faculty of Medicine, Mbarara University of Science and Technology, Mbarara, Uganda

[2]Institute of Allied Health Sciences, Clarke International University, Kampala, Uganda

[3]Uganda Cancer Institute, Kampala, Uganda

[4]Infectious Diseases Research Collaboration, Kampala, Uganda

[5]African Population and Health Research Center, Nairobi, Kenya

[6]Department of Medicine, Cumming School of Medicine, University of Calgary, Alberta, Canada

[7]Snyder Institute for Chronic Diseases, University of Calgary, Alberta, Canada

[8]Department of Microbiology, Immunology and Infectious Diseases, Cumming School of Medicine, University of Calgary, Alberta, Canada

[9]Department of Pediatrics, University of Montreal, Quebec, Canada

Authors' full names, institutional addresses, and email addresses

Ivan Mugisha Taremwa,
Mbarara University of Science and Technology, P.O. Box 1410, Mbarara, Uganda
Email: imugisha@ymail.com

Nixon Niyonzima,
Uganda Cancer Institute, P.O. Box 3935, Kampala, Uganda
Email: nixon.niyonzima@uci.or.ug

Scholastic Ashaba,
Mbarara University of Science and Technology, P.O. Box 1410, Mbarara, Uganda

Email: sashaba@must.ac.ug

Cecilia Akatukwasa,
Infectious Diseases Research Collaboration, P.O. BOX 7475, Kampala, Uganda
Email: cakatukwasa@idrc-uganda.org

Elizabeth Kemigisha,
Mbarara University of Science and Technology, P.O. Box 1410, Mbarara, Uganda
African Population and Health Research Center, P.O. Box 10787 00100, Nairobi, Kenya
Email: ekemigisha@must.ac.ug

Deusdedit Tusubira,
Mbarara University of Science and Technology, P.O. Box 1410, Mbarara, Uganda
Email: dtusubira@must.ac.ug

Carlrona Ayebazibwe,
Clarke International University, P.O Box 7782, Kampala, Uganda.
Email: acarlrona36@gmail.com

Shiller Atuhaire,
Mbarara University of Science and Technology, P.O. Box 1410, Mbarara, Uganda
Email: atuhaireshiller@gmail.com

May Y. Choi,
University of Calgary, 3330 Hospital Drive NW, Calgary, Alberta, T2N 4N1
Email: may.choi@ucalgary.ca

Guido van Marle,
University of Calgary, 3330 Hospital Drive NW, Calgary, Alberta, T2N 4N1
Email: vanmarle@ucalgary.ca

Hume A. Heather,
Université de Montréal, Department of Paediatrics, CHU Sainte-Justine 3175, Côte-Sainte-Catherine, Montréal, QC, Canada, H3T 1C5
Email: heather.hume@umontreal.ca

Bernard Natukunda,
Mbarara University of Science and Technology, P.O. Box 1410, Mbarara, Uganda
Email: bnatukunda@must.ac.ug

Corresponding author
Email: imugisha@ymail.com (IMT)

Abstract

Blood transfusion is crucial in supporting the treatment of patients with cancer. However, in poor-resource settings, blood and blood components remain scarce, and their perceived health risks may hinder their use. This study explored the perceptions of blood availability, safety, and acceptability among caregivers and healthcare providers of patients with cancer at Mbarara Regional Referral Hospital in southwestern Uganda.

This study employed a qualitative design involving in-depth interviews and key informant interviews with caregivers of patients with cancer and healthcare providers, between September and December 2024. The interviews were audio-recorded, transcribed, translated, and analyzed using thematic content analysis.

Of the 30 participants enrolled, 16 were caregivers of patients with cancer (mean age: 36; standard deviation, SD: 10.65 years) and 14 were healthcare providers (mean: 6.5; SD: 5.77 years of practice). Blood availability relied mostly on volunteer donors in educational institutions, and blood shortages were common during school recesses. Other highlighted additional constraints included donor complacency, limited availability of some blood groups, and disruptions during disease outbreaks. While most caregivers trusted that transfused blood was safe, some expressed concerns regarding adverse events and infection risks. Caregivers highly accepted blood transfusion as a life-saving intervention, but concerns relating it to end-of-life care, as well as cultural and religious beliefs were documented, and these were reported to influence transfusion acceptance. These findings show that caregivers experienced persistent shortages in blood availability in Uganda, while transfusion acceptance for patients with cancer was driven by system-level and sociocultural constraints. Since patients with cancer require

frequent transfusions, addressing challenges of blood supply and transfusion uptake through coordinated interventions is essential to support cancer care.

Introduction

Cancer is the second leading cause of death globally (1), with an estimated 20 million new cases diagnosed in 2022, including over 2.6 million in Africa and about 36,000 in Uganda (2). Patients with cancer frequently develop reduced blood cell counts, necessitating blood transfusions.

Blood transfusion, the administration of whole blood and its components, remains critical in supportive cancer care. In fact, 6% of all transfusions in sub-Saharan Africa (SSA) are given for cancer care (3), and blood and component therapy are now included on the World Health Organization (WHO) model list of essential medicines (4). Of concern, frequent blood transfusions among patients with cancer are undermined by the documented risks (5,6). For instance, studies conducted in the United States and the United Kingdom reported that while patients and healthcare providers recognized transfusion as beneficial, there were concerns related to adverse events following transfusion, infection risk, and treatment burden (7–9). Relatedly, caregivers of children with severe anemia in Uganda worried about Human Immunodeficiency Virus (HIV) infection and transfusion with incompatible blood (10). In Saudi Arabia, concerns related to the transmission of HIV, Hepatitis B, and C were reported (11). These perceived safety concerns as well as cultural and religious barriers may negate transfusion uptake (12); thus compromising the care of patients who require frequent transfusion. This study aimed to explore caregivers' and healthcare providers' perceptions of blood availability, safety, and acceptability at Mbarara Regional Referral Hospital, southwestern Uganda.

Materials and Methods

Study design, duration, and sites

This qualitative study used a case study approach, considering Mbarara Regional Blood Bank (MRBB) and Mbarara Regional Referral Hospital (MRRH) as an interconnected blood transfusion unit, from donor to recipient. This approach allowed an in-depth, interpretive exploration of processes and experiences within an integrated blood transfusion system. The study was conducted between September and December 2024. Both MRBB and MRRH are under the Ministry of Health, located in Mbarara City, approximately 270 kilometres southwest of Kampala, the Capital City of Uganda. MRRH is a tertiary healthcare facility serving more than 6 million people across 13 districts in southwestern Uganda and the neighboring districts of Rwanda, Tanzania, and the eastern Democratic Republic of Congo. MRBB is an establishment of Uganda Blood Transfusion Services (UBTS), mandated with the collection, processing, and supply of safe blood and components in the southwestern region.

Study participants and size

The study enrolled caregivers of patients with cancer at MRRH, as well as selected healthcare providers at both MRRH and MRBB. Caregivers of patients with cancer were recruited as proxies for the in-depth interviews (IDIs) rather than patients themselves because many patients with cancer requiring transfusion are clinically unstable and psychologically vulnerable (13), making additional research participation potentially burdensome and ethically challenging. Furthermore, as the transfusion processes in this setting are largely family-mediated, with caregivers playing a key role in consent and transfusion decision-making (14), caregivers' perspectives may influence transfusion uptake. Therefore, given that the study aimed to explore the perceptions of transfusion acceptance and access rather than individual illness experience, caregivers were considered for IDIs.

The sample size was guided by achieving data saturation (15,16). Thus, 30 interviews were deemed appropriate as responses became repetitive and no new insights were identified. Of these, 16 IDIs were conducted with caregivers of patients with cancer, sampled purposively from both the pediatric and adult cancer wards. Eleven IDIs were conducted with healthcare providers, including six nurses, three laboratory personnel, and two medical officers. Additionally, three key informant interviews (KIIs) were carried out, comprising two ward in-charges at MRRH, and one quality assurance focal person from UBTS. Participants were selected based on their roles in blood transfusion. Nurses at the blood bank are responsible for donor eligibility assessment and blood collection, while hospital-based nurses provide patient care and oversee transfusion administration. Medical officers assess patients' need for transfusion and make the transfusion orders. Laboratory personnel at the blood bank screen donor units for transfusion-transmissible infections (TTIs) and issue safe blood units, whereas hospital-based laboratory staff conduct pre-transfusion testing and participate in the investigations of any transfusion reactions. The UBTS quality assurance focal person oversees quality assurance processes and training support. By selecting participants from diverse groups (Table 1), we aimed to generate comprehensive and representative responses.

Table 1: Characteristics of participants

Interview type	Description	Number of interviewees per site (category by gender)	
		MRRH	MRBB
In-depth Interviews	Adult caregivers (parents, spouse, or child) of patients with cancer	16 (11 Females, 5 Males	Not Applicable
	Healthcare providers	8 (3 Females, 5 Males)	3 (1 Female, 2 Males)
Key Informant Interviews	Health care providers with the oversight responsibility	2 (1 Female, 1 Male)	1 Male
Total		26 (15 Females, 11 Males)	4 (1 Female, 3 Males)

Participant enrollment

The treating medical officer identified the eligible patients with a cancer diagnosis who had previously received blood transfusions. The medical officer informed patients about a study seeking to recruit their caregivers and obtained verbal consent or assent to disclose the patients' diagnosis and transfusion history to the research team. With permission, a designated member of the research team met with the patient and their caregiver in privacy, provided study information, assessed caregiver eligibility, obtained informed consent, and then scheduled the interview.

Inclusion and exclusion criteria

Caregivers were eligible if they were aged ≥ 18 years, had provided care to a patient with cancer for at least 1 month, were aware that the patient had been diagnosed with cancer, and the patient had received at least two blood transfusions in the past 3 months, including a transfusion during the current hospitalization as part of their clinical care. Healthcare providers at MRRH were eligible if they had worked in the cancer unit for at least 3 months and had prescribed (for medical officers), administered blood (for nurses), or issued blood (for laboratory staff). Healthcare providers at MRBB were eligible if they had worked in their roles for at least 3 months. Key informants were considered if they had prescribed blood transfusions for patients with cancer or were involved in regional training for MRBB. Caregivers were excluded if the patient required continuous bedside monitoring and no temporary cover was available to allow participation in a private interview. We also excluded caregivers who had mental instability or if the patient died before the interview. Healthcare providers who were on annual leave during the study period were not eligible for inclusion.

Sampling procedure

Purposive sampling was used to recruit caregivers and healthcare providers who met the inclusion criteria, ensuring inclusion of respondents with relevant experience. Key informants were recruited using convenience sampling due to their specialized roles.

Data collection tools and approach

Data were collected using semi-structured in-depth interview and key informant interview (KII) topic guides developed in English from existing literature (7,9,11,17). The topic guides included open-ended questions to explore the perceptions of blood needs, availability of blood, safety concerns, acceptability, and alternatives to blood transfusion (Supplementary Material). IDIs with caregivers were conducted in commonly spoken local languages (Runyankore-Rukiiga, Runyoro-Rutoro, Rufumbiira, and Luganda), while interviews with healthcare providers were conducted in English. Each interview had a facilitator and a note-taker, who also managed audio recordings. Interviews were conducted in private rooms at the pediatric and adult wards at MRRH. Healthcare providers were interviewed at their convenience at workplaces. Trained research assistants with experience in qualitative research and fluent in interview languages supported data collection. Interviews lasted about 40-60 minutes and were audio-recorded. Probing questions were used to clarify responses and explore emerging issues, allowing for an iterative approach to data collection. Interviews continued until thematic saturation was reached (18), and detailed field notes were taken. One of the authors (IMT) transcribed all interviews and verified transcript accuracy. Interviews conducted in local languages were independently translated into English by two other authors (AC and AS), and IMT reviewed translations to ensure fidelity and resolved discrepancies.

Quality assurance

The interview guides were pilot-tested at MRRH to assess content validity and comprehension. These were then refined to improve clarity. The topic guides were reviewed by experts in blood transfusion, cancer, and qualitative research. To minimize social desirability bias, interviewers used open-ended questions, probing techniques, and careful framing of sensitive questions. Transcription accuracy was ensured by comparing transcripts with the audio recordings.

Data management and analysis

Descriptive statistics were used to summarize the participants' demographic characteristics. Qualitative data were analyzed using an inductive thematic approach. Three individuals (IMT, CA, and AS) independently coded the transcripts guided by the study objectives. An initial codebook was developed using conventional content analysis after the coders independently reviewed the first six transcripts and identified key concepts. The codebook was applied to subsequent transcripts and iteratively refined as new codes and themes emerged. The coding process involved a line-by-line review of each transcript to identify patterns, relationships, and emerging themes. The coders met to compare coding and resolved discrepancies through discussion and re-reading the relevant sections of the transcripts together, and agreed on the best-fit interpretation of the data. Further analysis involved collapsing multiple codes that had a similar concept (19). All transcripts were uploaded into NVivo Software (20), checked for accuracy, and systematically analyzed. The 6-step thematic analysis process was followed: data familiarization, initial code generation, code refinement and theme development, consensus-based theme review, theme naming and definition, and synthesis of findings (21). Where language was potentially identifying (such as individuals and organizations), the details were removed. In reporting the findings, “most participants” refers to over 75%, “some participants” refers to 25-75%, and “a few participants” refers to less than 25%.

Ethical considerations

The study obtained ethics approval from the Mbarara University of Science and Technology Research and Ethics Committee (MUST-2024-505) and the Uganda National Council for Science and Technology (HS4551ES). Written informed consent was obtained prior to study activities in which participants were informed about their right to decline or withdraw from the study and the confidentiality of their responses. They authorized the audio recording and the publication of anonymized responses. Interviews were carried out in private rooms, and the audio recordings were transferred to a password-protected computer that was restricted to the authorized members of the study team. As caring for patients with cancer is associated with psychosocial distress, the study offered psychosocial support to participants through the psychiatry unit at MRRH.

Results

Study participants characteristics

Of the 30 participants enrolled, 16 were caregivers of patients with cancer (mean age: 36; SD: 10.65 years), and 14 were healthcare providers (mean: 6.5; SD: 5.77 years of practice) (Table 2).

Table 2: Demographic characteristics of participants

**Variable**	**N (%)**
Caregivers of patients with cancer (N=16)	
**Age category (Years)**	
< 35	9 (56.2%)
35-65	7 (43.8%)
**Sex**	
Female	11 (68.7%)
Male	5 (31.3%)
**Relationship with the patient**	
Child	4 (25.0%)
Parent	8 (50.0%)
Spouse	4 (25.0%)

**Marital status**	
Single	1 (6.3%)
Married	13 (81.2%)
Others (divorced, widowed)	2 (12.5%)
**Level of education**	
No formal education	1 (6.3%)
Primary	7 (43.7%)
Secondary level	6 (37.5%)
Tertiary	2 (12.5%)
**Religion**	
Protestant	8 (50.0%)
Catholic	4 (25.0%)
Muslim	2 (12.5%)
Pentecostal	2 (12.5%)
Healthcare providers (N=14)	
**Duration of healthcare service (Years)**	
≤5	7 (50.0%)
6-10	5 (35.7%)
>10	2 (14.3%)
**Sex**	
Female	5 (35.7%)
Male	9 (64.3%)
**Healthcare role**	
Medical Officer	4 (28.6%)
Nurse	6 (42.8%)
Laboratory staff	4 (28.6%)

Qualitative findings: Themes

The ensuing themes were: perceptions of blood availability, perceptions of blood safety, and acceptability of blood transfusion.

Theme 1: Perceptions of blood availability

Sub-theme 1: Perceived barriers to blood supply

School calendar dependence

Healthcare providers from UBTS stated that most of the blood donors were learners in educational institutions and thus they perceived the school calendar to significantly influence blood collection, with donations reportedly peaking during the school term and declining during the school recess;

> *"Over 80% of all the donated blood comes from school-going donors, and the availability of blood depends on school time. ..during the school term, we collect≥100 units, but this drops to < 20 units during the holidays." IDI-Healthcare Provider-7; Male*

Scarcity of some blood group donations

Healthcare providers reported a general scarcity of certain blood groups;

> *"There is a scarcity of O and RhD-negative donor groups. …this frustrates* patients and *their caregivers, who may resort to alternatives." IDI-Healthcare Provider-1; Male*

MRBB indicated challenges in mobilizing RhD-negative donors, noting that most of them were perceived to reside outside the region and would require transportation support to donate.

Incentive expectations undermining altruistic blood donation

A healthcare provider from UBTS reported that some donors thought that blood transfusion should be associated with costly incentives, as expressed;

> *"Some donors believe in entitlements and rewards like bicycles. ..others demand assurance of completely free medical services if they fell sick." IDI-Healthcare Provider-4; Female*

Low adult participation in blood donation programs due to lifestyle and attitude

A healthcare provider from UBTS perceived adult participation in blood donation to be limited, which was attributed to being too busy with their daily routines. Some of them expressed negative sentiments, as quoted;

> *"Some adults believe that blood donation is the responsibility of a specific group of people. …they express sentiments like, 'why should I donate? I won't be the one needing the blood anyway." IDI-Healthcare Provider-4; Female*

Impact of adverse weather on donor turnout and blood collections

Healthcare providers from UBTS indicated that cold weather negatively affected field operations. They described lower donor turnout and further reported that cold conditions made phlebotomy more challenging, were perceived to occasionally result in failed donations.

Fear during disease outbreaks limiting blood collection

Healthcare providers from UBTS recognized that disease outbreaks, such as Ebola, appeared to reduce communities' willingness to donate blood. They viewed community members as being less willing to engage in donation due to fear and self-isolation, as reported;

> *"During an outbreak, blood collections decline as field engagements are reduced. ..people self-isolate; reducing blood supply." IDI-Healthcare Provider- 2; Male*

Sub-theme 2: Perceived inequities affecting blood availability

Some caregivers and healthcare providers expressed concerns about blood donation as a key barrier to voluntary donation. They stated that communities sometimes held negative attitudes toward the donation process, influenced by a perceived sense of 'unmatched loyalty'. While blood donation is voluntary and non-remunerated, caregivers and healthcare providers reported that concerns about transfusion-related 'charges' incurred in healthcare facilities contributed to apprehension;

> ***Caregiver's report**: "Blood transfusion isn't for free! In private facilities, costs related to transfusion are included in a patient's medical bill. ..in government healthcare facilities, you offer 'a token of facilitation'.." IDI-Caregiver-16; 32 Years, Male*

> ***Healthcare provider report**: "In the communities, people are generally willing to donate blood. However, a common concern raised to us is: 'why do patients have to pay for a transfusion when we donate blood for free?" IDI-Healthcare Provider-4; Female*

Sub-theme 3: Perceived operational limitations to blood and component availability

Field collections as a barrier to the preparation of components

Healthcare providers from UBTS reported that the majority of blood donations were collected in the field, and they noted delays in transporting blood could sometimes limit its timely availability for component preparations;

> *"The blood collection teams travel to distant locations, often spending the entire day in the field. By the time the blood reaches the blood bank, it is no longer within the required time frame for immediate use or for preparing platelets or fresh frozen plasma." IDI-Healthcare Provider-2; Male*

Operational challenges undermining access to timely component availability

Healthcare providers perceived the breakdown of the only centrifuge used for platelet preparation as a major factor compromising the timely and adequate availability of platelets. The impact was worsened by prolonged equipment downtime due to the absence of resident servicing personnel.

Sub-theme 4: Perceived chronic scarcity of blood and its clinical consequences

Increasing demand vis-à-vis limited supplies

Healthcare providers described blood as a scarce and highly needed resource, with demands extending beyond cancer care. They noted that in situations of limited blood supply, prioritization often favored 'emergency cases' as described;

"During times of blood scarcity, there are prioritization decisions. Whenever a transfusion is requested for a patient with cancer and patients from other wards, the priority is the patient whose condition is more likely to be immediately stabilized by the transfusion. ..unfortunately, patients with cancer are often the least prioritized." IDI-Healthcare Provider-3; Female

Missed blood transfusions due to shortages

Most caregivers reported that their patients had missed transfusions due to blood shortages. They noted that this resulted in the rescheduling of medical appointments for surgeries and chemotherapy. Caregivers attributed some deaths to missed transfusions.

Overall, blood was perceived as insufficient. Healthcare providers perceived the system as structurally fragile that was overly dependent on school-going donors, whose contributions sustained the blood availability during school terms but created severe shortages during recess periods. Also, the scarcity of blood group O and all RhD-negative donors was reported. Moreover, community disengagement from donation due to competing priorities and negative attitudes mostly among adults emerged. Of concern, healthcare providers from UBTS reported that some donors expected material incentives and assurance of medical benefits. This approach was perceived to conflict with the principle of voluntary, non-remunerated donation. Moreover, transfusion-related charges were perceived to contradict the altruistic basis. Additionally, public health emergencies like disease outbreaks were reported to disrupt field activities. Notably, distant field operations and the breakdown of the centrifuge were also reported to constrain the preparation and availability of components. These factors were considered to contribute to chronic scarcity and rationing of the available blood, often prioritizing acute emergencies over patients with cancer, resulting in missed transfusions, treatment delays, and deaths that caregivers perceived as preventable.

Theme 2: Perceptions of blood safety

Sub-theme 1: Institutional trust in screening and laboratory safeguards

Healthcare providers and most caregivers expressed confidence in the safety of donor blood due to the rigorous pre-donation screening and laboratory testing protocols for TTIs, as quoted;

Caregiver report: *"I trust the safety of this blood. ..the blood bank team assured us that all donated units are tested, and blood units are only transfused if found free of infections. ..units found to be infected are discarded." IDI-Caregiver-11; 53 Years, Female*

Healthcare Provider report: *"The donor blood is safe. The Uganda Blood Transfusion Services use the best technology to screen the blood." IDI-Healthcare Provider-2; Male*

Sub-theme 2: Perceived limitation of donor screening and residual risk of TTIs

High-risk donors

A key informant from UBTS noted that some walk-in donors were primarily motivated by access to diagnostic testing for HIV and other infections. This perception raised concerns regarding the reliability of self-reported risk histories collected during donor assessment, as quoted;

"Some of the walk-in donors, including sex workers, come to donate blood with the intention of testing for HIV and other infections." KII-Healthcare Provider-1; Male

Residual risk of TTIs

A few caregivers expressed worry about the limitations of laboratory testing, noting that the residual risk of TTI raised fears of possible transfusion-transmission, as quoted;

"Early-stage infections may be missed during blood testing, risking transmission of HIV, hepatitis, or syphilis. ..a relative contracted HIV possibly through multiple childhood transfusions." IDI-Caregiver-10; 20years, Female

In some instances, caregivers' prior experiences with post-transfusion illness appeared to reinforce concerns about transfusion safety, even when a direct causal link was uncertain. One caregiver expressed concern based on prior experiences with bacterial infections, leading to skepticism about whether or not the transfusions were the cause, as expressed;

"After a blood transfusion, the patient got ill with a diagnosis of a bacterial infection. One cannot rule out whether it was through the transfusion." *IDI-Caregiver-1; 43years, Female*

Sub-theme 3: Concerns about adverse transfusion reactions and errors

A few caregivers described apprehension arising from lived experiences of transfusion reactions and suspected laboratory error, which shaped their perceptions of transfusion safety as reported;

"The patient started scratching her body. I (the caregivers) thought that the 'new blood' was mixing with the one it had found in the body." IDI- Caregiver-1; 43years, Female

"The child was transfused with the wrong blood group, which caused the body to swell. ..I worry that such an error can kill a patient!" IDI-Caregiver-9; 42years, Male

Sub-theme 4: Misbeliefs/Misconceptions

Because of adverse effects and the prevailing community misconceptions, a few caregivers expressed concerns and believed that donor blood was from non-human sources, as narrated;

"After transfusion, the patient often becomes restless. I (the caregiver) sometimes wonder where the blood comes from! ..other caregivers ask which animal did this blood come from?" IDI-Caregiver-4; 25 years, Female.

Generally, the perceptions of blood safety were shaped by institutional trust, personal experience, and socio-cultural beliefs. Most participants expressed confidence in the safety of donor blood based on the rigorous pre-donation screening and laboratory testing protocols. However, this trust was undermined by the perception that high-risk walk-in donors seek diagnostic testing, prior experiences with adverse transfusion events and purported

transmission of TTIs. Thus, trust in procedural safety coexisted with caution. In addition, sociocultural narratives and misconceptions influenced the perceptions of donor blood.

Theme 3: Acceptability of blood transfusion

Sub-theme 1: Enablers of blood transfusions uptake

Perceived benefit of restoring life and the patient's well-being

Healthcare providers and most caregivers viewed blood transfusions as highly beneficial in the care and management of patients with cancer. Most caregivers trusted that healthcare providers administered treatments, including transfusions, with the intent to heal. Transfusions helped alleviate symptoms such as fatigue, pallor, restlessness, and palpitations. A caregiver highlighted the life-impacting benefits of blood transfusion, as quoted;

> *"If he (the patient) hadn't been transfused, he likely wouldn't be alive. ..whenever he doesn't receive a transfusion, he feels unhappy and dissatisfied with the treatment, often complaining that he has missed out on a lot." IDI- Caregiver-7; 25years, Male*

Most caregivers expressed deep gratitude for the health improvements observed following transfusions, describing them as providing "a second chance at life" that restored energy and daily functioning. Healthcare providers similarly perceived blood transfusions as indispensable in clinical care, particularly for patients with cancer and those requiring palliative support. They highlighted that transfusions were essential for controlling bleeding, improving blood cell parameters, and sustaining physiological stability, which in turn enabled other therapeutic interventions.

Life-saving impact as a driver of transfusion acceptance

A caregiver reported that the patient's recovery from a near-death condition was clear evidence that blood transfusion saves lives and expressed willingness to accept transfusions, as reported;

"I (the caregiver) have witnessed how much better the patient feels after a transfusion. I support it (blood transfusion), and would never refuse it." *IDI-Caregiver-1; 43years, Female*

Failure of the alternative remedies

A few caregivers stated transitioning from alternative remedies to formal healthcare, choosing to accept blood transfusions for their patients, as other remedies, such as herbal treatments, did not improve the patients' health conditions, as stated;

"After saucepans of herbs, he (the patient) didn't improve. ..I (the caregiver) took him to the hospital and accepted a blood transfusion." IDI-Caregiver-15; 48 years, Male

Sub-theme 2: Concerns of blood transfusions

Religious and cultural concerns

Caregivers belonging to the Jehovah's Witness denomination and some patients from the Kasese region and of Somali origin were reported to decline blood transfusions. Healthcare providers indicated that the Jehovah's Witnesses involved their pastoral leaders, who strongly condemned blood transfusion, arguing that 'it is forbidden and sacred'. To them, 'blood symbolizes life, and God has reserved its use for His divine authority.' Patients and caregivers from Somalia and Kasese were reported to cite cultural beliefs and fears that transfused blood could transfer undesirable traits from the donor, as narrated;

***Jehovah's Witnesses' religious perspective on blood transfusion**: "Blood is sacred and represents life. ..only God has authority over it, and using it outside His will is forbidden. ..accepting transfusion is a violation of divine law." KII-Healthcare Provider-2; Female*

***Cultural perceptions of blood transfusion**: "I (caregiver) fear that if he (the patient) receives blood from another person, he will acquire their (blood donors') traits and*

illnesses. ..in our culture (Bakonzo), blood is more than just life. It is an identity and essence. ..we can not take that risk.' IDI-Caregiver-16; 32 Years, Male

"In our community (Somali), receiving blood touches our identity and family lineage. ..the foreign blood can bring unwanted behaviors!" IDI-Caregiver-6; 60, Female

Relating transfusion to end-of-life care

A few caregivers related blood transfusions to very sick and near-death patients, as quoted;

"I (the caregiver) grew up believing that when a patient reaches the stage of needing a transfusion, it often means they are about to die! ..the patient was too weak, and I feared that if she got a transfusion, she might die!" IDI-Caregiver-4; 25 years, Female

Sub-theme 3: Alternatives to blood transfusion

Drugs

Healthcare providers perceived patient management as multifaceted and extending beyond blood transfusion. They reportedly administered tranexamic acid, prophylactic anticoagulants, hematinics, vitamin supplementation, folic acid, and iron. These medications were prescribed as part of a comprehensive therapeutic approach aimed at controlling bleeding, preventing thromboembolic complications, and correcting the underlying defects.

Complementary and traditional approaches

Most caregivers indicated supplementing the care of patients with cancer with fruits and vegetables, such as mangoes, passion fruits, watermelons, hibiscus, beetroots, and a vegetable locally known as '*buga*'. Additionally, patients with cancer consumed foods like millet, liver, fresh blood from slaughtered animals (like cows, goats, sheep), and others used herbal concoctions comprising the leaves of avocado, pawpaw, jackfruit, and mango, among others. These remedies were viewed as supportive therapies intended to replenish blood, enhance the patient's overall

health, and aid in recovery. A caregiver reportedly gave a herbal concoction to a patient following a blood transfusion, as quoted;

> *"By the time the healthcare provider tells you that the patient needs a blood transfusion, the patient is critically ill. ..you accept the transfusion, but you also provide herbs to add to that blood!" IDI-Caregiver-6; 60 Year, Female*

Collectively, the acceptability of blood transfusion was shaped by therapeutic benefits, lived experiences of clinical improvement, trust, sociocultural and religious beliefs, and recourse to alternative therapies. On the contrary, blood transfusion was simultaneously constrained by deeply rooted religious and cultural beliefs where blood was not merely regarded as a biological fluid but as an embodiment of identity, lineage, essence, and moral attributes generating concerns that the transfused blood could transmit undesirable traits, illnesses, or spiritual qualities. Additional hesitation emerged from the perception that transfusion signifies impending death or extreme disease severity. Due to the barriers, alternatives to transfusion were opted for and sometimes were reportedly co-administered, illustrating medical pluralism.

Discussion

This study explored the perceptions of blood availability, safety, and acceptability among caregivers and healthcare providers of patients with cancer at Mbarara Regional Referral Hospital in southwestern Uganda. From this study, the availability of blood was affected by school holidays, donor complacency, high demand, scarcity of O and RhD-negative blood groups, rainy seasons, and disease outbreaks. Caregivers generally viewed blood as safe amidst concerns of adverse events and infection risks. Blood transfusions were highly valued; however, cultural and religious beliefs hindered their acceptability. Thus, alternatives such as medicines and complementary therapies were used.

Healthcare providers perceived the blood supply as fragile and largely reliant on institution-based donors, and it was characterized by marked seasonal fluctuations during school terms and recesses. This perception is supported by data from UBTS: in 2022-2023, only 313,659 units were collected, satisfying approximately 70% of the country's requirements (22) and falling short of the WHO's recommended minimum donation level of 1% of the population annually (23). The high dependency on educational institution-based donors reflects organized campaigns targeting students, a group perceived as a lower-risk population for TTIs (24), and aligns with patterns documented in low- and middle-income countries. In contrast, adult participation in voluntary blood donation was viewed as lower, mostly attributed to their socio-economic demands and, as some healthcare providers reported, a lax attitude. Additional challenges of blood availability were compounded by the perceived scarcity of O and RhD-negative blood groups, possibly due to a low donor-to-recipient ratio. As reported by previous studies (25–27), factors such as donor complacency and disease outbreaks remain a hindrance to blood collection. The above factors collectively were perceived to result in the scarcity of blood, undermining the healthcare provision by prolonging the waiting times and hospital stays. Moreover, the reports of 'token of facilitation' in the attempt to secure blood for the patients contravene the basis of altruism in blood banking. Further, private health facilities reportedly included 'blood transfusion-related costs' in their medical bills, likely reflecting charges for the transfusion services themselves rather than the cost of the blood unit. These practices were viewed to create a perception of commodification of blood and likely discourage sustainable participation by altruistically motivated donors, as previously reported (28). The reported malpractices require further research to inform the punitive measures. Given the challenges posed by the insufficiency of blood, there is a need for public awareness to address the persistent suboptimal annual blood collection in Uganda. More, although not explored

in this study, avoidable transfusions create an imbalance between blood supply and demand, leading to unavailability for patients with critical needs (29). This study adds to the advocacy for the proper utilization of blood as a scarce and expensive resource.

Caregivers of patients with cancer perceived blood safety with ‘mixed reactions’. Although blood was highly regarded as safe, concerns about the associated risks emerged. These perceptions reflect awareness of potential risks, even in systems that adhere to WHO-recommended safety standards. The safety attributes are evidence of the rigorous pre-donation screening, laboratory testing, and quality assurance protocols, aligning perceptions with reality and reinforcing trust in the blood supply. On the other hand, caregivers expressed worries about infections and adverse events, similar to previous reports (7,9–11,17). The perceived safety concerns underscore the challenges caregivers may face in balancing the perceived risks of blood transfusion with its life-saving benefits (11).

Blood transfusion was generally acceptable; however, underlying expression of discontent revealed safety and appropriateness concerns. The enormous acceptance correlates with the existing data on the indispensable life-saving role of blood transfusion. For instance, in the United States, blood transfusion was regarded as “critical” and “of greatest importance” (30), similar to the findings in a study conducted at Ohio Hospital, where the benefits of blood transfusion were difficult to discern due to the patient's disease condition (31). Notably, cultural and religious contempt of blood use was also reported, similar to previous studies (10,11,32), and may negatively influence transfusion uptake. Due to the limited availability, safety concerns, and objections to blood, the use of alternative therapy was reported. The prospect of alternative therapy remains complex and requires further research to safeguard patients' health.

While blood transfusion is generally perceived as acceptable and life-saving, various perceptions identified in this study may undermine both its perceived safety and acceptability. For instance, some caregivers viewed transfusion as interventions reserved for the ‘very sick patients and as a last resort’, which may explain the misbelief that transfused patients were likely to die. This perception is likely shaped by the clinical context in which transfusions are frequently administered after alternative treatments have failed or when the patient’s condition is severe. Thus associating transfusion symbolically with impending death rather than recovery. Moreover, delayed consent or hesitation in such cases can compromise timely management of severe anemia or hemorrhagic shock, thereby increasing the risk of mortality. Therefore, the perceived association between transfusion and death likely reflects temporal proximity rather than causation, and hemovigilance data indicate that transfusion-attributable mortality is rare and usually linked to identifiable adverse events (33,34). Further, caregivers reported concerns about donors in the window period, during which infections may not be detectable, contributing to fears of TTIs. Healthcare providers also perceived risk from ‘walk-in’ donors seeking infectious disease testing through donation, while caregivers reported anecdotes of individuals testing HIV-negative before transfusion but positive afterwards. Although TTIs remain theoretically possible, rigorous donor screening has markedly reduced the risk. Additionally, the perceptions that transfused blood was from non-human sources or the transfer of a donor’s trait are incorrect, as xenogeneic transfusion is not practiced due to immunological incompatibility, and red blood cells function solely in oxygen delivery. Finally, animal blood or herbal remedies can not physiologically substitute for transfusion in severe or chemotherapy-induced anemia. Of concern, in already resource-constrained settings, such unsupported perceptions may delay consent, reduce transfusion uptake,

and exacerbate avoidable morbidity, thereby compounding genuine structural challenges within blood systems.

The strengths of this study are highlighted by its dual focus on transfusion perceptions from both patients' caregivers and healthcare providers in a context that has not been studied before in SSA. On the other hand, the study findings ought to be interpreted in light of the following: its single-center design and the fact that its results may be skewed towards the studied categories, excluding blood donors. Despite these limitations, the findings offer critical insights into the caregivers' perception on blood availability, safety, and acceptability, which will guide future research.

Conclusions

This study reports divergent perceptions on blood availability, safety, and acceptability at a tertiary healthcare facility. The perceptions of blood availability reflected systemic challenges, including seasonal shortages, donor complacency, scarcity of certain blood groups, and increased clinical demand. Blood safety remains a worry due to the perceived risk of infections and adverse events. Also, blood acceptability was subjective, mainly affected by religious, cultural, and experiential misconceptions. Notably, perceptions that are not supported by evidence, such as beliefs in non-human sources of blood, transfer of personality, and inherent cause of death, may delay consent, reduce uptake, and exacerbate preventable mortality, compounding the existing structural challenges. Given that patients with cancer often require blood transfusions, efforts to ensure availability, safety, and improve acceptability are critical. Additionally, addressing both evidence-based and misinformed perceptions is essential for ensuring timely and appropriate use of blood. Thus, multipronged approaches such as information, education, and communication campaigns are essential for guiding communities on blood donation and improving the acceptability of blood transfusion.

Acknowledgments

We are grateful to all the study interviewees for their invaluable time and responses. We acknowledge Korukiiko Milliam, Nuwe Mworozi, and Tumukunde Penninah for supporting the data collection. We are indebted to Dr. Badru Kayongo, who offered psychosocial support to the caregivers. The authors are grateful to Katie Cooke for the support with NVivo software, data management guidance, and review of this manuscript.

Funding

This research was funded by the Government of Uganda through Mbarara University of Science and Technology, and MicroResearch Canada (24J_MUS_01). The funders did not influence the design, conduct, data acquisition, analysis, interpretation of the results, or the decision to publish.

References

1. Mathers CD. History of global burden of disease assessment at the World Health Organization. Archives of Public Health. 2020;78:1–13.
2. Sung H, Ferlay J, Siegel RL, Laversanne M, Soerjomataram I, Jemal A, et al. Global cancer statistics 2020: GLOBOCAN estimates of incidence and mortality worldwide for 36 cancers in 185 countries. CA Cancer J Clin. 2021;71(3):209–49.
3. Dhabangi A, Dzik WH, Idro R, John CC, Butler EK, Spijker R, et al. Blood use in sub-Saharan Africa: a systematic review of current data. 2019;59(7):2446-2454.
4. World Health Organization. WHO model list of essential medicines, 20th list (March 2017, amended August 2017). 2017. Available from: https://www.who.int/publications/i/item/eml-20 Accessed on 3 August 2025.
5. Goel R, Tobian AAR, Shaz BH. Noninfectious transfusion-associated adverse events and their mitigation strategies. Blood, The Journal of the American Society of Hematology. 2019;133(17):1831–9.
6. Hong H, Duque MA, Al Mana AF, Wu Y. Noninfectious transfusion-associated adverse events. Ann Blood. 2022;7:19.
7. Vetter TR, Adhami LF, Porterfield Jr JR, Marques MB. Perceptions about blood transfusion: a survey of surgical patients and their anesthesiologists and surgeons. Anesth Analg. 2014;118(6):1301–8.
8. Volkmer B, Lorencatto F, Stanworth SJ, Hirani SP, Francis JJ. Blood transfusion in haematology: A qualitative exploration of patients' and healthcare professionals' perceptions. Br J Health Psychol. 2022;27(4):1241–74.
9. Abdul-Aziz B, Lorencatto F, Stanworth SJ, Francis JJ. Patients' and health care professionals' perceptions of blood transfusion: a systematic review. Transfusion (Paris). 2018;58(2):446–55.

10. Dhabangi A, Idro R, John CC, Dzik WH, Opoka R, Siu GE, et al. Caregivers and community perceptions of blood transfusion for children with severe anaemia in Uganda. Transfusion Medicine. 2019;29(1):61–7.
11. Alsharidah AS, Alsuhaibani HA, Almansour BS, Alsharidah MS. Perceptions About Blood Transfusion Therapy Among the General Public and Healthcare Professionals in the Qassim Region of Saudi Arabia. J Blood Med. 2021;139–45.
12. Dei-Adomakoh Y, Asamoah-Akuoko L, Appiah B, Yawson A, Olayemi E. Safe blood supply in sub-Saharan Africa: challenges and opportunities. Lancet Haematol. 2021 Oct 1;8(10):e770–6.
13. Kibralew G, Wassie YA, Kelebie M, Rtbey G, Tadesse G, Melkam M, et al. Psychological distress among cancer patients in African countries: a systematic review and meta-analysis study. BMC Psychol. 2025;13(1):128.
14. Gambe RG, Clark J, Meddick-Dyson SA, Ukoha-Kalu BO, Nyaaba GN, Murtagh FEM. The roles and experiences of informal carers providing care to people with advanced cancer in Africa—A systematic review and critical interpretive analysis. PLOS Global Public Health. 2023;3(4):e0001785.
15. Fugard AJB, Potts HWW. Supporting thinking on sample sizes for thematic analyses: a quantitative tool. Int J Soc Res Methodol. 2015;18(6):669–84.
16. Braun V, Clarke V. (Mis) conceptualising themes, thematic analysis, and other problems with Fugard and Potts'(2015) sample-size tool for thematic analysis. Int J Soc Res Methodol. 2016;19(6):739–43.
17. Graw JA, Eymann K, Kork F, Zoremba M, Burchard R. Risk perception of blood transfusions–a comparison of patients and allied healthcare professionals. BMC Health Serv Res. 2018;18:1–8.
18. Guest G, Bunce A, Johnson L. How many interviews are enough? An experiment with data saturation and variability. Field methods. 2006;18(1):59–82.
19. Byrne D. A worked example of Braun and Clarke's approach to reflexive thematic analysis. Qual Quant. 2022;56(3):1391–412.
20. QSR International Pty Ltd. NVivo. 12 ed. [computer software]. Melbourne, Australia; 2018.
21. Braun V, Clarke V. Using thematic analysis in psychology. Qual Res Psychol. 2006;3(2):77–101.
22. https://redcrossug.org/health-and-social-services/blood-donor-recruitment/. Accessed on 21 August 2025.
23. Jacobs JW, Bates I, Cohn CS, Choudhury N, Owusu-Ofori S, Vrielink H, et al. The global blood donation index: an imperfect measure of transfusion need. The Lancet. 2024;404(10456):988–90.
24. Allain J. Current approaches to increase blood donations in resource-limited countries. Transfusion Medicine. 2019;29(5):297–310.
25. Aneke JC, Okocha CE. Blood transfusion safety; current status and challenges in Nigeria. Asian J Transfus Sci. 2017;11(1):1–5.
26. Weimer A, Tagny CT, Tapko JB, Gouws C, Tobian AAR, Ness PM, et al. Blood transfusion safety in sub-Saharan Africa: a literature review of changes and challenges in the 21st century. Transfusion (Paris). 2019;59(1):412–27.
27. Barro L, Drew VJ, Poda GG, Tagny CT, El-Ekiaby M, Owusu-Ofori S, et al. Blood transfusion in sub-Saharan Africa: understanding the missing gap and responding to present and future challenges. Vox Sang. 2018;113(8):726–36.
28. Bruers S. Blood donation and monetary incentives: a meta-analysis of cost-effectiveness. Transfus Med Rev. 2022;36(1):48–57.
29. Goodnough LT, Levy JH, Murphy MF. Concepts of blood transfusion in adults. The Lancet. 2013;381(9880):1845–54.
30. Odejide OO, Huntington SF, Shi E, Johnson KS, Tulsky JA, Abel GA. Perceptions of hospice and transfusion access among patients with advanced blood cancers: results from a best-worst scaling survey. Blood. 2021;138:3020.
31. Adams KW, Tolich D. Blood transfusion: the patient's experience. AJN The American Journal of Nursing. 2011;111(9):24–30.

32. Ringnes HK, Hegstad H. Refusal of medical blood transfusions among Jehovah's Witnesses: Emotion regulation of the dissonance of saving and sacrificing life. J Relig Health. 2016;55:1672–87.
33. Jhaveri P, Bozkurt S, Moyal A, Belov A, Anderson S, Shan H, et al. Analyzing real-world data of blood transfusion adverse events: Opportunities and challenges. Transfusion (Paris). 2022;62(5):1019–26.
34. World Health Organization. Global status report on blood safety and availability 2021. Geneva: World Health Organization; 2022. Available from: https://iris.who.int/handle/10665/356165. Accessed 11 September 2025.

List of legends

Supplementary Material_Data Collection Tool

Supporting Information_Research and Ethics Committee Approval
