# Supplementary material for: Caregivers’ and healthcare providers’ perceptions of blood availability, safety, and acceptability for patients with cancer in southwestern Uganda
Source: PLOS Glob Public Health. 2026 Sep 11;6(9):e0007203. doi: 10.1371/journal.pgph.0007203 (PMC13567789; doi:10.1371/journal.pgph.0007203)
Supplement: Supplementary file 2 [file pgph.0007203.s001.docx]

# **S1 Appendix: Interview guide**

**Introduction**

Good morning/afternoon. We are researchers from the Mbarara University of Science and Technology. We are conducting a study titled: “Assessment of Transfusion Safety and Efficacy among Cancer Patients in Uganda and Caregivers’ Perceptions on Blood Use.” We will organize an interview with you to find out your views and ideas regarding this topic. The research seeks to understand the caregivers' perceptions on blood use and availability in southwestern Uganda. Your responses will not in any way be linked to your identity and your responses will be kept confidential. You are kindly requested to participate in this study by voluntarily sharing your experiences and/or views.

Interview number _________________	Role: ___________________

**Socio-Demographic Characteristics**

Age of respondent [__ __] Years

Relationship with the patient: ___________________________________________________

Marital status:	Single ❒	Married ❒	Separated ❒	Divorced ❒	Widowed ❒	Cohabitation ❒

Education level: Primary ❒	Secondary level ❒	Tertiary ❒	No formal education ❒

Religion (optional): Protestant ❒	Catholic ❒	Muslim ❒	Traditional ❒ Other (specify) __________

**Patient’s caregiver level** *(to be answered by the caregivers’)*

Introduction

-Tell me about yourself

-How did you come to this hospital

-For how long have you been at this hospital?

-How is the patient now?

I understand your patient received a blood transfusion, tell me why you think the patient needed it. *Probe for:*

-Caregivers understanding of transfusion’s necessity

-What information did the healthcare provider give you about transfusion?

-Were you given information?

-Were you satisfied with the information?

What signs and symptoms did your patient have that would necessitate a blood transfusion? *Probe:*

-Do you think these signs and symptoms necessitated a blood transfusion?

-When the healthcare provider told you about blood transfusion, what concerns did you have?

Please tell us, how you feel about blood transfusion for your cancer patients. *Probe:*

-Family dynamics as a structure of blood transfusion acceptance or decline?

-How does your community feel about blood transfusion?

How did the patient react when they were told that they were to receive a blood transfusion? *Probe:*

- The patient's response to a transfusion reaction

What was the general mood of the patient after a blood transfusion?

How often has your patient been transfused? *(If multiply transfused)*:

-What is your feeling about frequent transfusion to the patient?

-How does your patient feel about frequent transfusions? Probe:

-Any emotions experienced about transfusions that caused (or could cause) distress

Do you think or believe that blood transfusion can result in new health problems? *Probe:*

-What are they and Why?

-How did you experience that with your patient?

In your view, what are the benefits of blood transfusion? *Probe:*

-Positive gains such as feeling better post-transfusion

-Shared experience such as improved strength, ability to work on own

-The physical or psychological benefits of the transfusion such as caseation of headache, fatigue

-Distinction between expected benefits vs instant and later benefits

-For any benefits, explore what exactly they benefited from the transfusion

How concerned or worried are you (about your patient) regarding the risks of blood transfusion? *Probe:*

-Risks of contracting infections like HIV, hepatitis, malaria, …

-Risks of transfusion reactions and death

-Risk of bacterial infection due to donor blood contamination

Do you think there may be a risk of non-human sources of donor blood and that this may affect the behavior of the recipient? Tell us more about it.

Do you think there could be some mistakes in the process of blood transfusion such as errors that may affect your patient? Tell us more about it. If yes, did you experience any

What alternatives to blood transfusion do you know? *Probe:*

-Trust and preference in the alternatives to blood transfusion

For your patient, were you informed of the decision to be given blood? *Probe:*

-Involvement of caregivers in transfusion decision-making,

-If counseling changed their perceptions

Were you given a chance to ask about blood transfusion and the consequences if you declined it? *Probe:*

-Lack of choice or questioning of the blood transfusion decision

Would you accept a blood transfusion for yourself or a relative/friend in the future? *Probe for as much as possible the reasons as to why, or why not*.

**Hospital healthcare provider level (Medical Officer, Nurse)**

Introduction

-Thank you for sacrificing some time to attend our interview.

-For how long have you been at the cancer ward?

-How many cancer patients do you have on the ward now? How has been the trend since you joined this hospital?

-In a week, how many of the cancer patients require blood transfusion?

Do you get the blood and components you need for all the cancer patients in the ward? If you don’t get it, why? *Probe:*

-Availability of whole blood, packed cells, platelets

-Availability of products such as cryoprecipitate and others

How much would you say cancer patients need transfusions as supportive therapy? *Probe for: Distinction between protecting health presently or long-term*

How do patients clinically feel or report after the patient was transfused? *Probe: Changes that you think were attributed to blood transfusion*

Do you find a noticeable hematological change in blood parameters such as Hb levels?

Please share with us some of your perspectives about blood transfusion. Share with us your perspectives on blood transfusion. *Probe:*

-Transfusion safety in terms of sterility for bacteria, malaria, and other TTIs

-Short vs long terms risk perceptions, especially with multiple allogeneic transfusion

As a health care provider who has been interacting with caregivers and patients, what are their concerns about blood transfusion?

What are the health benefits of blood transfusion to cancer patients? *Probe: Improved health of the patient (Keep going with daily life) and support to other treatments given to the patient*

In your view, what are the benefits of blood transfusion compared with other treatments?

As a health care provider, share with us the risks of blood transfusion. *Probe*:

-Contamination risk of transfused blood (bacteria, blood parasites and viruses)

-Transfusion adverse events such as transfusion reactions

-Risks compared to other treatments

-Generalized risks such as transfusion dependency

How concerned or worried are you about these perceived risks of blood transfusion?

Are there alternatives to blood transfusion for cancer patients? *Probe*: *Product availability (autologous, cell salvage)*

What factors do you think may influence the use of alternatives? (availability, suitability, and cost) *Probe*:

-Advantages of alternatives

-Disadvantages of alternatives

How often are the transfusion alternatives considered for cancer patients? *Probe*: *Reasons that transfusions may be the preferred or the only way available*

What challenges do you face with patients who need blood transfusions?

Share with us your experiences of blood transfusion. *Probe*:

-Favorable outcome (the patient improved and was discharged)

-Adverse events that compromised the patient’s life or even led to death

Are the caregivers of cancer patients informed of the decision or involved in the decision to transfuse their patients? *Probe*:

-Caregivers' involvement in decision-making to transfuse and counseling support if needed

-Caregivers are informed of the benefits, risks, and alternatives.

How can blood transfusion be improved and ensure that blood is safe for patients with cancer? *Probe: Personal experience based on the challenges.*

**Hospital healthcare provider (Laboratory staff)**

Introduction:

-Thank you for sacrificing some time to attend our interview.

-For how long have you been in the transfusion laboratory?

-How many blood transfusion requests do you receive for cancer patients in a day?

-How has been the trend since you joined this laboratory?

-In a week, how many transfusion requests of the cancer patients do you receive?

Do you receive enough units of blood and components you need from the blood bank for all the cancer patients in the ward? If you don’t get them, tell us why. *Probe*:

-Availability of whole blood, packed cells, platelets

-Availability of products such as cryoprecipitate and others

How much would you say cancer patients need transfusions as supportive therapy? *Probe*: *Distinction between protecting health presently or long-term*

Do you find a noticeable hematological change in blood parameters such as Hb or platelet levels?

Share with us your experiences of blood transfusion. *Probe*:

-Transfusion safety in terms of sterility for bacteria, malaria, and other TTIs

-Short vs long terms risk perceptions, especially with multiple allogeneic transfusion

With your interactions with healthcare workers and patients, what are the major concerns about blood transfusion?

What are the health benefits of blood transfusion to cancer patients? *Probe*: *Improved health of the patient (keep going with daily life) and support to other treatments given to the patient*

In your view, what are the benefits of blood transfusion?

As a health care provider, share with us the risks of blood transfusion. *Probe*:

-Contamination risk of transfused blood (bacteria, blood parasites and viruses)

-Transfusion adverse events such as transfusion reactions

-Risks compared to other treatments

-Generalized risks such as transfusion dependency

-Share experiences they have heard (risks), what outcomes? What led to the risks?

How concerned or worried are you about these perceived risks of blood transfusion?

Are there alternatives to blood transfusion for cancer patients? *Probe*: *Product availability (autologous, cell salvage)*

What factors do you think may influence the use of alternatives? (availability, suitability, and cost) *Probe*:

-Advantages of alternatives

-Disadvantages of alternatives

How often are the transfusion alternatives considered for cancer patients? *Probe*: *Reasons that transfusions may be the preferred or the only way available*

Share with us your experiences of blood transfusion. *Probe*:

-Favorable outcome (the patient improved and was discharged)

-Adverse events that compromised the patient’s life or even led to death. How many adverse events are reported?

Do you provide all the necessities for the blood transfusion process including giving sets?

If yes, what are they?

If no move to the next question.

How can blood transfusion be improved and ensure that blood is safe for patients with cancer? *Probe*: *Personal experience based on the challenges.*

**Blood bank healthcare provider (Laboratory Staff)**

Introduction:

-Thank you for sacrificing some time to attend our interview.

-For how long have you been at the blood bank?

-How many requests for blood do you receive from the hospitals?

-How has been the trend since you joined this blood bank?

Do you prepare blood units and components for all the hospital requests? If you don’t get it, why? *Probe*:

-Availability of whole blood, packed cells, platelets

-Availability of products such as cryoprecipitate and others

How much would you say cancer patients need transfusions as supportive therapy? *Probe*: *Distinction between protecting health presently or long-term*

Please share with us about the blood screening process at the blood bank before you issue it to the requesting facilities *Probe*: *If tests confirm bacterial contamination, malaria, and transfusion transmissible infections.*

Share with us your experiences of blood transfusion. *Probe*:

-Favorable outcome (the patient improved and was discharged)

-Adverse events that compromised the patient’s life or even led to death. How many adverse events are reported?

As a health care provider, share with us the risks of blood transfusion. *Probe*:

-Contamination risk of transfused blood (bacteria, blood parasites and viruses)

-Transfusion adverse events such as transfusion reactions

-Risks compared to other treatments

-Generalized risks such as transfusion dependency

How concerned or worried are you about these perceived risks of blood transfusion?

What in your view do you think can cause a breakdown in the aseptic process of donor blood?

What measures do you consider to minimize or eliminate contamination of donor blood? *Probe*:

-Tests to rule out bacterial contamination such as pH changes, cultures

-Tests to screen and confirm TTIs

-Risk of transfusion transmissible infections such as HIV, Hepatitis

Do you think we face a risk of other emerging infections? If yes, list them.

Are you satisfied with the technology used to screen and confirm TTIs? If not, tell us more and your recommendation.

Are there any breakdowns in the cold chain systems that may pose a hazard to blood? If yes, tell us more and your recommendation.

What quality assurance practices are in place to warrant blood testing protocol? Tell us more about it.

Share with us experiences of screening and issuing blood and components for transfusion. *Probe*:

-Favorable outcome (the patient improved and was discharged)

-Adverse events that compromised the patient’s life or even led to death

Have you had any reports of a transfusion reaction? Probe: If yes, what was the cause?

How can blood transfusion be improved and ensure that blood is safe for patients with cancer? *Probe*: *Personal experience based on the challenges.*

**Blood bank healthcare provider** (donor screening and bleeding staff)

Introduction:

-Thank you for sacrificing some time to attend our interview.

-For how long have you been working at the blood bank?

-How many potential donors do you receive a day, and how many are screened out? How has been the trend since you joined?

-In a week, how many donor units do you collect?

-Do you receive enough donors? If you don’t get them, tell us why.

What are the donors' considerations to ensure blood safety? *Probe*:

-Healthy donors

-Non-renumerated blood donors

-Least risk population including school children

Is the skin disinfection properly done to ensure blood safety? *Probe*: *Disinfectant and concentration being used*

Tell us about the disinfection protocol you use while in the field.

Is the phlebotomy process properly done to ensure blood safety? *Probe*:

-Not touching the disinfected area to feel the blood vein before venipuncture

-Avoiding double puncture if the first attempt is unsuccessful

-Use of diversion pouching

What in your view do you think can cause a breakdown in the aseptic process of blood collection?

What other ways can lead blood to be contaminated? *Probe*:

-Latent blood donors

-Donors who may have multiple and risky sex partners

- Emerging endemic infections

What measures do you consider to minimize or eliminate contamination of donor blood? *Probe*:

-Tests to rule out bacterial contamination such as pH changes, cultures

-Tests to screen and confirm TTIs

In your view, is the donor blood safe? *Probe*:

-Risk of bacterial contamination in blood

-Risk of hemo-parasites

How concerned or worried are you about the safety of blood and components for transfusion?

How can UBTS improve blood safety in this region?

In your view, do we have sufficient availability of blood? *Probe*:

-Supply vs demand

-Specific components such as platelets, fresh blood

What are the challenges that limit the stock of blood and components? *Probe*:

-Reliable donor

-Rare blood groups

-School holidays

How concerned or worried are you about the availability of blood and components for transfusion?

How can UBTS improve blood availability in this region?

**Hospital healthcare manager (Cancer ward in-charge)**

Introduction:

-Thank you for sacrificing some time to attend our interview.

-For how long have you overseen the cancer ward?

-How many cancer patients do you have on the ward now? How has been the trend since you joined this ward?

-In a week, how many of the cancer patients require blood transfusion?

Do you get the blood and components you need for all the cancer patients in the ward? If you don’t get it, why? *Probe*:

-Availability of whole blood, packed cells, platelets

-Availability of products such as cryoprecipitate and others

How much would you say cancer patients need transfusions as supportive therapy? *Probe: Distinction between protecting health presently or long-term*

What are the health benefits of blood transfusion to cancer patients? *Probe*: *Improved health of the patient (Keep going with daily life) and support to other treatments given to the patient*

In your view, what are the benefits of blood transfusion compared with other treatments?

As a health care provider, share with us the risks of blood transfusion. *Probe*:

-Contamination risk of transfused blood (bacteria, blood parasites and viruses)

-Transfusion adverse events such as transfusion reactions

-Risks compared to other treatments

-Generalized risks such as transfusion dependency

How concerned or worried are you about these perceived risks of blood transfusion?

Are there alternatives to blood transfusion for cancer patients? *Probe*: *Product availability (autologous, cell salvage),*

What factors do you think may influence the use of alternatives? (availability, suitability, and cost). *Probe*:

-Advantages of alternatives

-Disadvantages of alternatives

How often are the transfusion alternatives considered for cancer patients? *Probe*: *Reasons that transfusions may be the preferred or the only way available*

What in your view do you think can cause a breakdown in the aseptic conditions of the blood process?

Share with us your lived experiences of blood transfusion. *Probe*:

-Favorable outcome (the patient improved and was discharged)

-Adverse events that compromised the patient’s life or even led to death

How can blood transfusion be improved and ensure that blood is safe for patients with cancer? *Probe*: *Personal experience based on the challenges*

**Blood bank (Healthcare manager: Director)**

Introduction:

-Thank you for sacrificing some time to attend our interview.

-For how long have you been working at the blood bank?

-How many units of blood do you collect in a year and what are your targets?

What are the donors' considerations to ensure blood safety? *Probe*:

-Heath of donors

-Non-renumerated blood donors

-Least risk population including school children

-Implementing a diversion pouch to minimize donor blood contamination

What in your view do you think can cause a breakdown in the aseptic process of blood collection?

What other ways can lead blood to be contaminated? *Probe*:

-Latent blood donors

-Donors who may have multiple and risky sex partners

-Emerging endemic infections

What measures do you consider to minimize or eliminate contamination of donor blood? *Probe*:

-Tests to rule out bacterial contamination such as pH changes, cultures

-Tests to screen and confirm TTIs

In your view, is the donor blood safe? *Probe*:

-Risk of bacterial contamination in blood

-Risk of hemo-parasites

-Risk of transfusion transmissible infections such as HIV, Hepatitis

Is there a risk of other emerging infections? Tell us about them

Is the technology used to screen and confirm TTIs sufficient to warrant safe donor blood?

How concerned or worried are you about the safety of blood and components for transfusion?

How can UBTS improve blood safety in this region?

In your view, do we have sufficient availability of blood? *Probe*:

-Supply vs demand

-Specific components such as platelets, fresh blood

What are the challenges that limit the stock of blood and components? *Probe*:

-Budget available vs demand

-Human resource available

-Limitation of expiry dates

-Reliable donor

-Rare blood groups

-School holidays

How concerned or worried are you about the availability of blood and components for transfusion?

What are alternatives to blood and components?

How can UBTS improve blood availability in this region
