# Supplementary material for: Caregivers’ and healthcare providers’ perceptions of blood availability, safety, and acceptability for patients with cancer in southwestern Uganda
Source: PLOS Glob Public Health. 2026 Sep 11;6(9):e0007203. doi: 10.1371/journal.pgph.0007203 (PMC13567789; doi:10.1371/journal.pgph.0007203)
Supplement: Supplementary file 3 [file pgph.0007203.s002.docx]

**To explore the perceptions of blood availability, safety, and acceptability among caregivers and healthcare providers of patients with cancer at Mbarara Regional Referral Hospital in southwestern Uganda**.

**Findings from in-depth interviews and key informant interviews.**

We conducted twenty-seven in-depth interviews (IDIs) and 3 key informant interviews (KIIs) with adult caregivers of patients with cancer, healthcare providers at Mbarara Regional Referral Hospital, and Mbarara Regional Blood Bank. The details of the study participants are given in Tables 1.

**Table 1a: Details of caregivers of patients with cancer**

| **Participant ID** | **Sex** | **Age** | **Relationship with the patient** | **Marital status** | **Education level** | **Religion** | **Occupation** |
| --- | --- | --- | --- | --- | --- | --- | --- |
| IDI-01-Caregiver | Female | 43 | Parent | Married | Diploma | Catholic | Self-employed (Business) |
| IDI-02-Caregiver | Female | 29 | Spouse | Married | Primary | Protestant | Self-employed (Business) |
| IDI-03-Caregiver | Female | 27 | Parent | Single | University | Catholic | Unemployed (Housewife) |
| IDI-04-Caregiver | Female | 25 | Parent | Divorced | Primary | Pentecostal | Farmer |
| IDI-05-Caregiver | Female | 33 | Spouse | Married | Secondary | Catholic | Farmer |
| IDI-06-Caregiver | Female | 60 | Spouse | Married | None | Protestant | Farmer |
| IDI-07-Caregiver | Male | 25 | Parent | Married | Secondary | Protestant | Self-employed (Casual laborer) |
| IDI-08-Caregiver | Female | 40 | Parent | Married | Primary | Protestant | Farmer |
| IDI-09-Caregiver | Male | 42 | Child | Married | Primary | Protestant | Farmer |
| IDI-10-Caregiver | Female | 20 | Spouse | Married | Secondary | Protestant | Self-employed (Business) |
| IDI-11-Caregiver | Female | 53 | Child | Windowed | Primary | Catholic | Farmer |
| IDI-12-Caregiver | Female | 36 | Parent | Married | Secondary | Protestant | Tailor |
| IDI-13-Caregiver | Female | 34 | Child | Married | Secondary | Pentecostal | Farmer |
| IDI-14-Caregiver | Male | 29 | Parent | Married | Secondary | Protestant | Self-employed (Builder) |
| IDI-15-Caregiver | Male | 48 | Parent | Married | Primary | Muslim | Self-employed-(Bussiness) |
| IDI-16-Caregiver | Male | 32 | Child | Married | Primary | Muslim | Farmer |

**Table 1b: Details of healthcare providers (Mbarara Regional Referral Hospital and Mbarara Regional Blood Bank**

| **Participant ID** | **Sex** | **Role** | **Years of work** |
| --- | --- | --- | --- |
| KII-01-Manger-Blood Bank | Male | Laboratory Quality Assurance Manager | 14 |
| KII-02-Manger-Hospital | Female | Medical Officer | 2 |
| KII-03-Manger-Hospital | Male | Medical Officer | 6 |
| IDI-01-Laboratory-Hospital | Male | Laboratory technologist | 4 |
| IDI-02- Healthcare Worker | Male | Laboratory technologist | 23 |
| IDI-03-Healthcare Worker | Female | Medical Officer | 8 |
| IDI-04-Healthcare Worker | Male | Nurse | 3 |
| IDI-05-Healthcare Worker | Female | Nurse | 7 |
| IDI-06-Healthcare Worker | Female | Medical Officer | 2 |
| IDI-07-Healthcare Worker | Male | Nurse | 3 |
| IDI-08-Healthcare Worker | Male | Nurse | 6 |
| IDI-09- Healthcare Worker | Female | Nurse | 10 |
| IDI-10- Healthcare Worker | Male | Nurse | 1 |
| IDI-11- Healthcare Worker | Male | Laboratory technologist | 2 |

The summary of the study participants is given in Table 2.

**Table 2: Summary of study participants**

| Interview type | Description | Number of interviewees per site (category by gender) | |
| --- | --- | --- | --- |
|  |  | MRRH | MRBB |
| In-depth Interviews | Adult caregivers (parents, spouse, or adult sons/ daughters) of patients with cancer | 16 (11 Females, 5 Males | Not Applicable |
|  | Healthcare providers | 8 (3 Females, 5 Males) | 3 (1 Female, 2 Males) |
| Key Informant Interviews | Health care providers with the oversight responsibility | 2 (1 Female, 1 Male) | 1 Male |
| Total |  | 26 (15 Females, 11 Males) | 4 (1 Female, 3 Males) |

Among the caregivers of patients with cancer (N=16), eleven were females, the majority (eight participants) were parents, and therefore caring for their children. Thirteen were married, and eight caregivers were protestants.

Among the healthcare providers, seven of them had worked for at least six years and above. More, nine of them were males, and six were nurses. The participants’ sociodemographic details are given in Table 3.

**Table 3: Demographic characteristics of participants**

| **Variable** | **N (%)** |
| --- | --- |
| *Caregivers of patients with cancer (N=16)* | |
| **Age category (Years)**  < 35  35-65 | 9 (56.2%)  7 (43.8%) |
| **Sex**  Female  Male | 11 (68.7%)  5 (31.3%) |
| **Relationship with the patient**  Child  Parent  Spouse | 4 (25.0%)  8 (50.0%)  4 (25.0%) |
| **Marital status**  Single  Married  Others (divorced, widowed) | 1 (6.3%)  13 (81.2%)  2 (12.5%) |
| **Level of education**  No formal education  Primary  Secondary level  Tertiary | 1 (6.3%)  7 (43.7%)  6 (37.5%)  2 (12.5%) |
| **Religion**  Protestant  Catholic  Muslim  Pentecostal | 8 (50.0%)  4 (25.0%)  2 (12.5%)  2 (12.5%) |
| *Healthcare providers (N=14)* | |
| **Duration of healthcare service (Years)**  ≤5  6-10  >10 | 7 (50.0%)  5 (35.7%)  2 (14.3%) |
| **Sex**  Female  Male | 5 (35.7%)  9 (64.3%) |
| **Healthcare role**  Medical Officer  Nurse  Laboratory staff | 4 (28.6%)  6 (42.8%)  4 (28.6%) |

**Perceptions of blood availability**

Blood availability was perceived as generally insufficient and structurally constrained by donor dependence patterns, logistical limitations in collection and processing, and variability in demand. Participants described a system highly dependent on school-based donations, vulnerable to seasonal disruptions, and affected by shortages of O and RhD negative blood groups as well as components like platelets and fresh frozen plasma. More operational inefficiencies, such as transport delays from the field and equipment breakdowns, were as well perceived to contribute to intermittent shortages and rationing of the scarce blood products.

***Perceived barriers to blood supply***

Dependence on school-based blood donation cycles: Healthcare providers from UBTS stated that most of the blood donors were students in educational institutions, and thus they perceived the school calendar to significantly influence blood collection, with donations reportedly peaking during the school term and declining during the school recess;

*“Over 80% of all the donated blood comes from school-going donors, and the availability of blood depends on school time. ..during the school term, we collect≥100 units, but this drops to < 20 units during the holidays.” (IDI, Healthcare Provider 7, Male)*

Scarcity of certain blood groups: Healthcare providers reported a general scarcity of certain blood groups;

*“There is a scarcity of O and RhD-negative donor groups. …this frustrates patients and their caregivers, who may resort to alternatives.” (IDI, Healthcare Provider 1, Male)*

MRBB indicated challenges in mobilizing RhD-negative donors, noting that most of them were perceived to reside outside the region and would require transportation support to donate.

Incentive expectations undermining altruistic blood donation: A healthcare provider from UBTS reported that some donors expected material incentives or benefits in exchange for donating blood, including tangible rewards such as bicycles or assurances of free medical services, as expressed;

*“Some donors believe in entitlements and rewards like bicycles. ..others demand assurance of completely free medical services if they fell sick.” (IDI, Healthcare Provider 4, Female)*

Barriers to adult participation in blood donation: A healthcare provider from UBTS perceived adult participation in blood donation to be low due to daily life commitments. Some of the healthcare providers also expressed negative sentiments regarding adults’ willingness to donate blood, as quoted;

*“Some adults believe that blood donation is the responsibility of a specific group of people. …they express sentiments like, ‘why should I donate? I won’t be the one needing the blood anyway.”* *(IDI, Healthcare Provider 4, Female)*

Impact of adverse weather on donor turnout and blood collections: Healthcare providers from UBTS indicated that adverse weather negatively affected donor turnout during field collections. They reported that cold conditions made phlebotomy more challenging and were occasionally associated with failed donation attempts.

Fear during disease outbreaks limiting blood collection: Healthcare providers from UBTS recognized that disease outbreaks, such as Ebola, appeared to reduce communities’ willingness to donate blood. They viewed community members as being less willing to engage in donation due to fear and self-isolation, as reported;

*“During an outbreak, blood collections decline as field engagements are reduced. ..people self-isolate; reducing blood supply.” (IDI, Healthcare Provider 2, Male)*

***Perceived barriers to voluntary blood donation***

Some caregivers and healthcare providers expressed concerns that acted as barriers to voluntary blood donation. They reported that communities sometimes held negative attitudes toward the donation process, partly influenced by a perceived lack of reciprocity between blood donation and access to transfusion services. While blood donation is voluntary and non-remunerated, caregivers and healthcare providers reported that concerns about transfusion-related expenses incurred in healthcare facilities contributed to apprehension about donating blood, as expressed;

*“Blood transfusion isn’t for free! In private facilities, costs related to transfusion are included in a patient’s medical bill. ..in government healthcare facilities, you offer ‘a token of facilitation’..” (IDI, Caregiver 16, 32 Years, Male)*

*“In the communities, people are generally willing to donate blood. However, a common concern raised to us is: ‘why do patients have to pay for a transfusion when we donate blood for free?” (IDI, Healthcare Provider 4, Female)*

***Perceived turnaround time and operational constraints affecting blood component preparation***

Field collections as a barrier to the preparation of components: Healthcare providers from UBTS reported that the majority of blood donations were collected in the field, and they noted that delays in transporting blood could sometimes limit its timely availability for component preparations;

*“The blood collection teams travel to distant locations, often spending the entire day in the field. By the time the blood reaches the blood bank, it is no longer within the required time frame for immediate use or for preparing platelets or fresh frozen plasma.” (IDI, Healthcare Provider 2, Male)*

Centrifuge breakdown and prolonged equipment downtime affecting component preparation: Healthcare providers perceived the breakdown of the only centrifuge used for platelet preparation as a major factor compromising the timely and adequate availability of platelets. The impact was worsened by prolonged equipment downtime due to the absence of resident servicing personnel.

***Perceived chronic scarcity of blood and its clinical consequences***

Increasing demand vis-à-vis limited supplies: Healthcare providers described blood as a scarce and highly needed resource, with demands extending beyond cancer care. They noted that in situations of limited blood supply, prioritization often favored ‘emergency cases’ as described;

*“During times of blood scarcity, there are prioritization decisions. Whenever a transfusion is requested for a patient with cancer and patients from other wards, the priority is the patient whose condition is more likely to be immediately stabilized by the transfusion. ..unfortunately, patients with cancer are often the least prioritized.”* (*IDI, Healthcare Provider 3, Female)*

Missed blood transfusions due to shortages: Most caregivers reported that their patients had missed transfusions due to blood shortages. They noted that this resulted in the rescheduling of medical appointments for surgeries and chemotherapy. Caregivers attributed some deaths to missed transfusions.

**Perceptions of blood safety**

Blood safety perceptions were largely shaped by institutional trust in screening systems, but also tempered by concerns regarding residual risk of transfusion-transmissible infections, donor risk disclosure, and possible adverse transfusion reactions. While most participants trusted laboratory safeguards, some expressed concerns related to limitations of testing windows, procedural errors, and cultural misconceptions.

***Institutional trust in screening and laboratory safeguards***

Healthcare providers and most caregivers expressed confidence in the safety of donor blood due to the rigorous pre-donation screening and laboratory testing protocols for TTIs, as quoted;

*“I trust the safety of this blood. ..the blood bank team assured us that all donated units are tested, and blood units are only transfused if found free of infections. ..units found to be infected are discarded.” (IDI, Caregiver 11, 53 Years, Female)*

*“The donor blood is safe. The Uganda Blood Transfusion Services use the best technology to screen the blood.” (IDI, Healthcare Provider 2, Male)*

***Perceived limitation of donor screening and residual risk of TTIs***

High-risk donors: A key informant from UBTS noted that some walk-in donors were primarily motivated by access to diagnostic testing for HIV and other infections. This perception raised concerns regarding the reliability of self-reported risk histories collected during donor assessment, as quoted;

*“Some of the walk-in donors, including sex workers, come to donate blood with the intention of testing for HIV and other infections.” (KII, Healthcare Provider 1, Male)*

Residual risk of TTIs: A few caregivers expressed worry about the limitations of laboratory testing, noting that the residual risk of TTI raised fears of possible transfusion-transmission, as quoted;

*“Early-stage infections may be missed during blood testing, risking transmission of HIV, hepatitis, or syphilis. ..a relative contracted HIV possibly through multiple childhood transfusions.” (IDI, Caregiver 10, 20years, Female)*

In some instances, caregivers’ prior experiences with post-transfusion illness appeared to reinforce concerns about transfusion safety, even when a direct causal link was uncertain. One caregiver expressed concern based on prior experiences with bacterial infections, leading to skepticism about whether or not the transfusions were the cause, as expressed;

*“After a blood transfusion, the patient got ill with a diagnosis of a bacterial infection. One cannot rule out whether it was through the transfusion.*” *(IDI, Caregiver 1, 43years, Female)*

***Concerns about adverse transfusion reactions and errors***

A few caregivers described apprehension arising from lived experiences of transfusion reactions and suspected laboratory error, which shaped their perceptions of transfusion safety as reported;

*“The patient started scratching her body. I (the caregiver) thought that the ‘new blood’ was mixing with the one it had found in the body.” (IDI, Caregiver 1, 43years, Female)*

*“The child was transfused with the wrong blood group, which caused the body to swell. ..I worry that such an error can kill a patient!” (IDI, Caregiver 9, 42years, Male)*

***Misbeliefs/Misconceptions***

Because of adverse effects and the prevailing community misconceptions, a few caregivers expressed concerns and believed that donor blood was from non-human sources, as narrated;

*“After transfusion, the patient often becomes restless. I (the caregiver) sometimes wonder where the blood comes from! ..other caregivers ask which animal did this blood come from?” (IDI, Caregiver 4, 25 years, Female)*

**Acceptability of blood transfusion**

Blood transfusion was widely accepted and perceived as life-saving, particularly among cancer patients requiring repeated transfusions. Acceptance was driven by perceived clinical benefit, lived experiences of recovery, and necessity within oncology care. However, acceptability was moderated by religious beliefs, cultural perceptions, and preference for complementary therapies.

***Enablers of blood transfusions uptake***

Perceived benefit of restoring life and the patient’s well-being: Healthcare providers and most caregivers viewed blood transfusions as highly beneficial in the care and management of patients with cancer. Most caregivers trusted that healthcare providers administered treatments, including transfusions, with the intent to heal. Transfusions helped alleviate symptoms such as fatigue, pallor, restlessness, and palpitations. A caregiver highlighted the life-impacting benefits of blood transfusion, as quoted;

*“If he (the patient) hadn’t been transfused, he likely wouldn’t be alive. ..whenever he doesn’t receive a transfusion, he feels unhappy and dissatisfied with the treatment, often complaining that he has missed out on a lot.” (IDI, Caregiver 7, 25years, Male)*

Most caregivers expressed deep gratitude for the health improvements observed following transfusions, describing them as providing “a second chance at life” that restored energy and daily functioning. Healthcare providers similarly perceived blood transfusions as indispensable in clinical care, particularly for patients with cancer and those requiring palliative support. They highlighted that transfusions were essential for controlling bleeding, improving blood cell parameters, and sustaining physiological stability, which in turn enabled other therapeutic interventions.

Life-saving impact as a driver of transfusion acceptance: A caregiver reported that the patient’s recovery from a near-death condition was clear evidence that blood transfusion saves lives and expressed willingness to accept transfusions, as reported;

*“I (the caregiver) have witnessed how much better the patient feels after a transfusion. I support it (blood transfusion), and would never refuse it*.” *(IDI, Caregiver 1, 43years, Female)*

Failure of the alternative remedies: A few caregivers stated transitioning from alternative remedies to formal healthcare, choosing to accept blood transfusions for their patients, as other remedies, such as herbal treatments, did not improve the patients’ health conditions, as stated;

*"After saucepans of herbs, he (the patient) didn’t improve. ..I (the caregiver) took him to the hospital and accepted a blood transfusion.”* (*IDI, Caregiver 15, 48 years, Male)*

***Fears and misconceptions about blood transfusion***

Religious and cultural beliefs: Caregivers belonging to the Jehovah’s Witness denomination and some patients from the Kasese region and of Somali origin were reported to decline blood transfusions. Healthcare providers indicated that the Jehovah’s Witnesses involved their pastoral leaders, who strongly condemned blood transfusion, arguing that ‘it is forbidden and sacred’. To them, ‘blood symbolizes life, and God has reserved its use for His divine authority.’ Patients and caregivers from Somalia and Kasese were reported to cite cultural beliefs and fears that transfused blood could transfer undesirable traits from the donor, as narrated;

*“Blood is sacred and represents life. ..only God has authority over it, and using it outside His will is forbidden. ..accepting transfusion is a violation of divine law.”* *(KII, Healthcare Provider 2, Female)*

*“I (caregiver) fear that if he (the patient) receives blood from another person, he will acquire their (blood donors’) traits and illnesses. ..in our culture (Bakonzo), blood is more than just life. It is an identity and essence. ..we can not take that risk.” (IDI, Caregiver 16, 32 Years, Male)*

*“In our community (Somali), receiving blood touches our identity and family lineage. ..the foreign blood can bring unwanted behaviors!” (IDI, Caregiver 6, 60 Years, Female)*

Fear associated with blood transfusion: A few caregivers related blood transfusions to very sick and near-death patients, as quoted;

*“I (the caregiver) grew up believing that when a patient reaches the stage of needing a transfusion, it often means they are about to die! ..the patient was too weak, and I feared that if she got a transfusion, she might die!”* *(IDI, Caregiver 4, 25 Years, Female)*

***Complementary and supportive therapeutic practices alongside blood transfusion***

Drugs: Healthcare providers perceived patient management as multifaceted and extending beyond blood transfusion. They reportedly administered tranexamic acid, prophylactic anticoagulants, hematinics, vitamin supplementation, folic acid, and iron. These medications were prescribed as part of a comprehensive therapeutic approach aimed at controlling bleeding, preventing thromboembolic complications, and correcting the underlying defects.

Complementary and traditional approaches: Most caregivers indicated supplementing the care of patients with cancer with fruits and vegetables, such as mangoes, passion fruits, watermelons, hibiscus, beetroots, and a vegetable locally known as ‘*buga*’. Additionally, patients with cancer consumed foods like millet, liver, fresh blood from slaughtered animals (like cows, goats, sheep), and others used herbal concoctions comprising the leaves of avocado, pawpaw, jackfruit, and mango, among others. These remedies were viewed as supportive therapies intended to replenish blood, enhance the patient’s overall health, and aid in recovery. A caregiver reportedly gave a herbal concoction to a patient following a blood transfusion, as quoted;

*“By the time the healthcare provider tells you that the patient needs a blood transfusion, the patient is critically ill. ..you accept the transfusion, but you also provide herbs to add to that blood!” (IDI, Caregiver 6, 60 Year, Female)*
