# Supplementary material for: Caregivers’ and healthcare providers’ perceptions of blood availability, safety, and acceptability for patients with cancer in southwestern Uganda
Source: PLOS Glob Public Health. 2026 Sep 11;6(9):e0007203. doi: 10.1371/journal.pgph.0007203 (PMC13567789; doi:10.1371/journal.pgph.0007203)
Supplement: Supplementary file 4 [file pgph.0007203.s004.docx]

Kampala, Uganda

15^th^ June 2026

Professor Ferdinand C Mukumbang,

PLOS Global Public Health

Dear Professor Ferdinand C Mukumbang,

**RE: Responses to the reviewers Manuscript ID: [PGPH-D-26-01258]- [EMID:8eac9832aa771c19]**

We sincerely appreciate the reviewer’s critical comments and guidance on our manuscript entitled **“Caregivers’ and healthcare providers’ perceptions of blood availability, safety, and acceptability for patients with cancer in southwestern Uganda” with Manuscript ID: [PGPH-D-26-01258]-[EMID:8eac9832aa771c19]**

**Journal Requirements:**

**Comment 1**. In the ethics statement in the Methods, you have specified that verbal consent was obtained. Please provide additional details regarding how this consent was documented and witnessed, and state whether this was approved by the IRB.

**Response**: Thank you for this comment. Under the Materials and Methods section, specifically sub-section titled: ‘Participant enrollment’, we would like to clarify that verbal consent or assent referred to was obtained during participant eligibility screening, and was distinct from the written informed consent obtained from adult caregiver of patients with cancer prior to study participation.

Specifically, the treating medical officer obtained verbal consent or assent from eligible patients with cancer before disclosing their cancer diagnosis and transfusion history to the study team for study enrollment. Following this initial permission, a member of the research team met with the patient with cancer and the caregiver, provided detailed study information, assessed eligibility, and obtained written informed consent from the caregiver before any study procedures were conducted.

To improve clarity, we have revised the ‘Participant Enrollment’ section of the manuscript to explicitly distinguish the verbal consent or assent used to authorize the disclosure of the patients information for recruitment from the written informed consent obtained from study participants. We have also clarified that all study procedures including the recruitment process were approved by the Mbarara University of Science and Technology Research Ethics Committee. The change is seen in the Track change document, page 8, lines 158-166.

**Comment 2**. Please provide a detailed online Financial Disclosure statement. This is published with the article. It must therefore be completed in full sentences and contain the exact wording you wish to be published.

a) State the initials, alongside each funding source, of each author to receive each grant. For example: “This work was supported by the National Institutes of Health (####### to AM; ###### to CJ) and the National Science Foundation (###### to AM).”

b) State what role the funders took in the study. If the funders had no role in your study, please state: “The funders had no role in study design, data collection and analysis, decision to publish, or preparation of the manuscript.”

For more information, please go to our submission guidelines:
https://journals.plos.org/globalpublichealth/s/submission-guidelines#loc-financial-disclosure-statement

**Response**: Thank you for this guidance. We have revised the Financial disclosure statement to comply with the journal’s requirements by identifying the authors associated with each funding source and clarifying the role of the funders. We have also clarified that the funders had no role in study design, data collection and analysis, decision to publish, or preparation of the manuscript.

The revised statement now reads: ‘This research was funded by the Government of Uganda through Mbarara University of Science and Technology (grant awarded to EK, who mentored IMT in the conduct of this research), and by MicroResearch Canada (24J_MUS_01 to IMT). The funders had no role in study design, conduct, data acquisition, analysis, interpretation of the results, or the decision to publish.’ The change is seen in the Track change document, pages 30 and 31, lines 645-650.

**Comment 3**. Please ensure that the funders and grant numbers match between the Financial Disclosure field and the Funding Information tab in your submission form. Note that the funders must be provided in the same order in both places as well.

**Response**: We take note of this critical requirement. We confirm that we have reviewed and revised the funding information to ensure consistency between the Financial disclosure statement in the manuscript and the Funding information section of the submission form. The funders, grant numbers, author recipients, and the order in which the funding sources are listed have been harmonized across both locations. Thank you.

**Comment 4**. Please update your online Competing Interests statement. If you have no competing interests to declare, please state: "The authors have declared that no competing interests exist."

**Response**: We appreciate this reminder. We have updated the Competing Interests statement in the manuscript and the online submission system to read: ‘The authors have declared that no competing interests exist.’

**Comment 5**. We note that your Data Availability Statement is currently as follows: "The relevant data used is available in the manuscript."

Please confirm at this time whether or not your submission contains all raw data required to replicate the results of your study. Authors must share the “minimal data set” for their submission. PLOS defines the minimal data set to consist of the data required to replicate all study findings reported in the article, as well as related metadata and methods (https://journals.plos.org/globalpublichealth/s/data-availability#loc-minimal-data-set-definition).

For example, authors should submit the following data:

- The values behind the means, standard deviations and other measures reported;
- The values used to build graphs;
- The points extracted from images for analysis.

Authors do not need to submit their entire data set if only a portion of the data was used in the reported study.

If your submission does not contain these data, please either upload them as Supporting Information files or deposit them to a stable, public repository and provide us with the relevant URLs, DOIs, or accession numbers. For a list of recommended repositories, please see https://journals.plos.org/globalpublichealth/s/recommended-repositories.

If there are ethical or legal restrictions on sharing a de-identified data set, please explain them in detail (e.g., data contain potentially sensitive information, data are owned by a third-party organization, etc.) and who has imposed them (e.g., an ethics committee). Please also provide contact information for a data access committee, ethics committee, or other institutional body to which data requests may be sent. If data are owned by a third party, please indicate how others may request data access.

**Response**: We acknowledge that the ‘minimal dataset’ used was not initially provided. We confirm that a S1 Data (Supporting Information file with minimal dataset) has now been uploaded as well. We confirm that this file has as well been included on the list of legends in the revised manuscript.

Also, a revision of the Data Availability Statement has been considered. The new Data Availability Statement reads: ‘The minimal dataset underlying the findings of this study has been provided as a supplementary file (S1 Dataset). The dataset includes de-identified qualitative interview excerpts, thematic summaries, and supporting quotations derived from in-depth interviews and key informant interviews conducted with caregivers of patients with cancer and healthcare providers. Further inquiries regarding the dataset may be directed to the corresponding author upon reasonable request, in accordance with institutional ethical guidelines.’

**Comment 6**. "Supplementary Material.docx" is currently uploaded as an 'Other' file type, which is not viewable by reviewers. Please ensure that all files meant for review are uploaded as 'Supporting Information' and include a legend in the manuscript.

**Response**: We apologize for this error. We have revised the ‘Supplementary Material.docx’ to 'Supporting Information' and we confirm that this already appears as a legend in the manuscript.

**Comment 7**. If the reviewer comments include a recommendation to cite specific previously published works, please review and evaluate these publications to determine whether they are relevant and should be cited. There is no requirement to cite these works unless the editor has indicated otherwise.

**Response**: We confirm that there was no reviewer recommendation to cite a specific previously published work.

**Additional Editor Comments (if provided):**
[Note: HTML markup is below. Please do not edit.]

**Response**: We acknowledge receipt of the marked-up file, and we confirm that we did not edit the file. We used it to revise the manuscript as per the reviewers comments.

**Reviewers’ comments:**
**Reviewer's Responses to Questions**

**Comments to the Author**
**Comment 1**. Does this manuscript meet PLOS Global Public Health’s [publication criteria](https://track.editorialmanager.com/CL0/https:%2F%2Fjournals.plos.org%2Fglobalpublichealth%2Fs%2Fcriteria-for-publication/1/010f019ec9cb2eb0-5349e38d-ae4c-4ba0-b403-5f5eeb34bfb6-000000/zojxzBD0UhtpWGyVHToEJQQuQjNrxR-JyMPe97sDW9M=258)? Is the manuscript technically sound, and do the data support the conclusions? The manuscript must describe methodologically and ethically rigorous research with conclusions that are appropriately drawn based on the data presented.

Reviewer #1: Partly

Reviewer #2: Yes

**Response**: We thank the reviewer for this feedback. We appreciate Reviewer #2 assessment that the manuscript is technically sound and that the data support the conclusions. We confirm that the revised file is much improved, and hopefully these revisions have strengthened the manuscript and further support the rigor of the study and the validity of its conclusions. Thank you.

**Comment 2**. Has the statistical analysis been performed appropriately and rigorously?

Reviewer #1: Yes

Reviewer #2: N/A

**Response**: We are pleased that Reviewer #1 found that the statistical analysis was performed appropriately and rigorously, and we note that Reviewer #2 highlighted that this item was not applicable. We have not modified the statistical analysis.

**Comment 3**. Have the authors made all data underlying the findings in their manuscript fully available (please refer to the Data Availability Statement at the start of the manuscript PDF file)?

The [PLOS Data policy](https://track.editorialmanager.com/CL0/https:%2F%2Fjournals.plos.org%2Fglobalpublichealth%2Fs%2Fdata-availability/1/010f019ec9cb2eb0-5349e38d-ae4c-4ba0-b403-5f5eeb34bfb6-000000/wePpYgqIJZLP9bD_EU5PpNTSHbYb0fAMGOoRlwXRLZw=258) requires authors to make all data underlying the findings described in their manuscript fully available without restriction, with rare exception. The data should be provided as part of the manuscript or its supporting information, or deposited to a public repository. For example, in addition to summary statistics, the data points behind means, medians and variance measures should be available. If there are restrictions on publicly sharing data—e.g. participant privacy or use of data from a third party—those must be specified.

Reviewer #1: No

Reviewer #2: Yes

**Response**: We sincerely apologize and acknowledge that the ‘minimal dataset’ used was not initially provided. We confirm that a S1 Data (Supporting Information file with minimal dataset) has now been uploaded as well. We confirm that this file has as well been included on the list of legends in the revised manuscript.

**Comment 4**. Is the manuscript presented in an intelligible fashion and written in standard English?
PLOS Global Public Health does not copyedit accepted manuscripts, so the language in submitted articles must be clear, correct, and unambiguous. Any typographical or grammatical errors should be corrected at revision, so please note any specific errors here.

Reviewer #1: Yes

Reviewer #2: Yes

**Response**: We thank both reviewers for their positive assessment of the manuscript’s clarity and language quality. We are grateful that both reviewers found the manuscript to be presented in an intelligible fashion and written in standard English. We have nonetheless carefully proofread the revised manuscript (file named: <<R1_Perceptions of blood in Uganda_Track Change>>) to ensure clarity, corrected any minor typographical issues, and improved the overall readability where necessary.

**Review Comments to the Author**
Please use the space provided to explain your answers to the questions above. You may also include additional comments for the author, including concerns about dual publication, research ethics, or publication ethics. (Please upload your review as an attachment if it exceeds 20,000 characters)

**Reviewer #1:**

This manuscript addresses an important and underexplored issue regarding perceptions of blood availability, safety, and acceptability among caregivers and healthcare providers of patients with cancer in Uganda. The topic is highly relevant to global public health, transfusion systems, and cancer care in low-resource settings. The manuscript is generally well structured, and the qualitative approach is appropriate for exploring complex sociocultural and system-level perceptions. The study has several strengths, including inclusion of both caregivers and healthcare providers, use of in-depth interviews and key informant interviews, and attention to ethical considerations. The findings provide valuable insights into how structural shortages, sociocultural beliefs, and trust in healthcare systems shape transfusion-related perceptions and practices.
However, several areas require strengthening before the manuscript is suitable for publication.

**Comment 1**: First, the methodological framing as a “case study approach” requires further clarification. The authors should explain how the case boundaries were defined, which type of case study design was adopted, and how the case study framework informed sampling, analysis, and interpretation.

**Response**: We thank the reviewer for this highlight. In the Materials and Methods section, specifically ‘Study design, duration, and sites’, we have revised this section to provide a clearer methodological framing of the case study approach. Specifically, we have clarified that an instrumental case study design was used and have explicitly defined the case boundaries as the full transfusion pathway linking Mbarara Regional Blood Bank and Mbarara Regional Referral Hospital, from blood donation through processing, distribution, clinical decision-making, and transfusion.

Additionally, we have clarified that the case study framework informed purposive sampling across key points in the transfusion pathway, guided data collection across both institutions, and supported thematic analysis and interpretation of findings within the bounded system.

The revised section of the manuscript now reads: ‘This qualitative study used an instrumental case study approach, examining Mbarara Regional Blood Bank (MRBB) and Mbarara Regional Referral Hospital (MRRH) as an interconnected blood transfusion unit, from donor to recipient. The case was defined as the full transfusion pathway linking the two institutions, encompassing blood donation, processing, distribution, clinical decision-making, and transfusion, and was geographically and functionally bound to these sites. The case study framework informed purposive sampling across key points in the transfusion pathway, guided data collection across both institutions, and supported thematic analysis and interpretation of the findings within the bounded system. This approach allowed an in-depth, interpretive exploration of processes and experiences within an integrated blood transfusion system.’ The change is seen in the Track change document, page 6, lines 110-117.

**Comment 2**: Second, the manuscript would benefit from stronger reporting of qualitative rigor and reflexivity. While coding procedures are described, there is limited discussion of researcher positionality, reflexive practice, credibility strategies, dependability, confirmability, or transferability. The authors should elaborate on how trustworthiness was ensured beyond coder discussions and transcript review. The authors should confirm if they followed the Consolidated Criteria for Reporting Qualitative Research (COREQ) to ensure all reporting standards for qualitative studies are met.

**Responses**: We thank the reviewer for this critical comment. We also acknowledge that additional clarification was needed regarding qualitative rigor and reflexivity shared in the initial manuscript. In the revised manuscript specifically subsection titled: ‘Data management and analysis’, we have strengthen the reporting of trustworthiness and to explicitly described the strategies used to ensure credibility, dependability, confirmability, and transferability beyond the coding process.

In the revised manuscript, we have clarified that analytic rigor was enhanced through multiple strategies, including independent coding by three researchers, iterative codebook development, regular consensus meetings, and documentation of analytic decisions to support dependability and confirmability. We have also noted that reflexive discussions were held throughout the analytic process to critically examine assumptions and interpretations and to minimize potential researcher bias. More, we have clarified that credibility was supported through triangulation of analyst perspectives and iterative review of emerging themes, while transferability was supported through thick description of the study context and participant experiences.

The revised section of ‘Data management and analysis’ now reads: ‘Qualitative data were analyzed using an inductive, team based thematic approach incorporating codebook development and iterative consensus coding. While the analysis was informed by the study objectives, no predetermined coding framework was applied. Instead, codes were generated from the data through repeated reading of transcripts and identification of concepts expressed by the participants. Three individuals (IMT, CA, and AS) independently reviewed and coded the transcripts. An initial codebook was developed using conventional content analysis after the coders independently reviewed the first six transcripts and was iteratively refined as new codes and themes emerged during analysis.

The coding process involved a line-by-line review of each transcript to identify patterns, relationships, and emerging themes. To enhance analytic rigor and minimize bias, three members of the research team (IMT, CA, and AS) independently coded transcripts and compared interpretations through regular consensus meetings. Discrepancies were resolved through discussion and re-reading the relevant sections of the transcripts together, and agreed on the best-fit interpretation of the data. Further analysis involved grouping similar codes to develop themes, which were refined through iterative team discussion.

All transcripts were uploaded into NVivo Software (19), checked for accuracy, and systematically analyzed. A structured 6-step thematic analysis process was used to to guide the analytical process, including data familiarization, initial code generation, code refinement and theme development, consensus-based theme review, theme naming and definition, and synthesis of findings (20). In reporting the findings, qualitative frequency descriptions such as “most participants” “some participants” and “a few participants” were used to indicate the relative prevalence of views across interviews without implying statistical quantification.’ The change is seen in the Track change document, pages 11 and 12, lines 227-252.

More, we confirm that all transcripts were systematically managed using NVivo software, and an audit trail of coding and theme development was maintained. Lastly, we confirm that the study was guided by the Consolidated Criteria for Reporting Qualitative Research (COREQ) checklist, and the manuscript has been checked against COREQ standards to ensure comprehensive reporting of qualitative methods and findings.

**Comment 3**: Third, some conclusions appear stronger than warranted by the qualitative data. Statements suggesting that misconceptions may exacerbate preventable mortality or references to “malpractices” requiring punitive measures should be presented more cautiously unless directly supported by the findings.

**Response**: We thank the reviewer for this comment. We acknowledge that such statements in the initial conclusion were overly strong for a qualitative study and required more cautious interpretation.

In the revised manuscript, we have moderated the language to ensure that all conclusions are directly grounded in the data and appropriately framed within the limits of qualitative inference. Specifically, we have revised statements that implied stronger causal implications by removing references to potential effects on mortality and replacing them with more measured language. We have also softened wording suggesting direct influence on outcomes and instead emphasize that participants’ perceptions and misconceptions may shape or influence transfusion-related decision-making. In addition, we have highlighted the social and symbolic meanings attached to blood in a more interpretive manner, consistent with qualitative findings. We believe these revisions have improved the balance, accuracy, and interpretive rigor of the conclusion while ensuring alignment with the study design and reported findings.

The revised conclusion section now reads: ‘This study reports divergent perceptions on blood availability, safety, and acceptability at a tertiary healthcare facility. The perceptions of blood availability reflected systemic challenges, including seasonal shortages, donor complacency, scarcity of certain blood groups, and increased clinical demand. Blood safety remains a worry due to the perceived risk of infections and adverse events. Also, blood acceptability was subjective, mainly affected by religious, cultural, and experiential misconceptions. Notably, some perceptions that are not supported by evidence, such as beliefs in non-human sources of blood, transfer of personality, and inherent cause of death reflect the social and symbolic meanings attached to blood and may influence how patients and caregivers approach transfusion-related decision making.

Given that patients with cancer often require blood transfusions, efforts to ensure availability, safety, and improve acceptability are critical. Additionally, addressing both evidence-based and misinformed perceptions is essential for ensuring timely and appropriate use of blood. Thus, multipronged approaches such as information, education, and communication campaigns may help improve understanding of blood donation and improving the acceptability of blood transfusion.’ The change is seen in the Track change document, pages 29 and 30, lines 650-665.

**Comment 4**: Fourth, the analysis could be deepened by exploring variations across participant groups. For example, sociocultural and religious perceptions may differ meaningfully across communities, caregiver categories, or healthcare provider roles, yet these distinctions are not explored analytically.

**Response**: We are grateful to the reviewer for this critical insight. We acknowledge that exploring variation across participant groups can strengthen qualitative analysis. To this, we have revisited the findings and revised the ‘Discussion section’ to highlight differences in perceptions between participant categories. Specifically, we now clarify that caregivers of patients with cancer tended to emphasize sociocultural, religious, and experiential concerns regarding blood transfusion, whereas healthcare providers more frequently highlighted structural and system-level constraints affecting blood availability and use. The change is seen in the Track change document, page 25, lines 557-561.

**Comment 5**: Fifth, the Data Availability Statement does not appear fully aligned with PLOS requirements. The statement that “relevant data used is available in the manuscript” is insufficiently detailed. The authors should clarify whether anonymized transcripts, coding frameworks, or supporting qualitative datasets are available, and if restrictions apply due to confidentiality, these should be explicitly stated.

**Response**: We sincerely apologize and acknowledge that the ‘minimal dataset’ used was not initially provided. We confirm that a S1 Data (Supporting Information file with minimal dataset) has now been uploaded as well. We confirm that this file has as well been included on the list of legends in the revised manuscript. Also, a revision of the Data Availability Statement has been considered.

**Comment 6**: Minor revisions are also recommended regarding language editing, grammar, spacing, and repetitive phrasing.

**Response**: We are grateful for your critical insights. We confirm that we have reviewed the entire manuscript, and the language editing, grammar, spacing and repetitive phrasing have been addressed in the revised manuscript (file titled: <<R1_Perceptions of blood in Uganda_Track Change>>).

**Comment 7**: Overall, this manuscript addresses a highly important topic and has potential to contribute meaningfully to the literature on transfusion practices and cancer care in sub-Saharan Africa. With revisions to strengthen methodological transparency, qualitative rigor, and interpretive caution, the manuscript would be substantially improved.

**Response**: Thank you so much for the critical review and insightful comments. We appreciate your feedback.

**Reviewer #2**:

Overall, the manuscript presents a qualitative exploration of perceptions surrounding blood availability, safety, and acceptability among caregivers and healthcare providers in southwestern Uganda. The study addresses an important clinical and operational gap in cancer care and transfusion services within resource-limited settings in sub-Saharan Africa. The qualitative design is appropriate for the study objectives, and the findings provide valuable insights into sociocultural beliefs, safety perceptions, blood availability challenges, and factors influencing transfusion acceptability.
The manuscript is generally well structured and demonstrates good integration of participant narratives with existing literature. The topic is highly relevant to strengthening transfusion services, cancer care, and patient-centered care in resource-limited settings. However, several areas require clarification and refinement before the manuscript can fully meet the publication standards of PLOS ONE.

**Comment 1**: Specifically, the authors should consider improving methodological clarity regarding the thematic analysis approach, particularly the alignment between the cited reflexive thematic analysis literature and the consensus/codebook-oriented analytic procedures described in the methods section.

**Response**: We thank the reviewer for this critical highlight. We agree that greater methodological clarity was needed regarding the alignment between the thematic analysis framework and the analytic procedures described in the manuscript. In response, we have revised the ‘Data management and analysis section’ of the manuscript to improve consistency and clarity.

The revised Data management and analysis section now reads: ‘Qualitative data were analyzed using an inductive, team based thematic approach incorporating codebook development and iterative consensus coding. While the analysis was informed by the study objectives, no predetermined coding framework was applied. Instead, codes were generated from the data through repeated reading of transcripts and identification of concepts expressed by the participants. Three individuals (IMT, CA, and AS) independently reviewed and coded the transcripts. An initial codebook was developed using conventional content analysis after the coders independently reviewed the first six transcripts and was iteratively refined as new codes and themes emerged during analysis.

The coding process involved a line-by-line review of each transcript to identify patterns, relationships, and emerging themes. To enhance analytic rigor and minimize bias, three members of the research team (IMT, CA, and AS) independently coded transcripts and compared interpretations through regular consensus meetings. Discrepancies were resolved through discussion and re-reading the relevant sections of the transcripts together, and agreed on the best-fit interpretation of the data. Further analysis involved grouping similar codes to develop themes, which were refined through iterative team discussion.

All transcripts were uploaded into NVivo Software (19), checked for accuracy, and systematically analyzed. A structured 6-step thematic analysis process was used to guide the analytical process, including data familiarization, initial code generation, code refinement and theme development, consensus-based theme review, theme naming and definition, and synthesis of findings (20). In reporting the findings, qualitative frequency descriptions such as “most participants” “some participants” and “a few participants” were used to indicate the relative prevalence of views across interviews without implying statistical quantification.’

We believe these revisions improve methodological transparency and ensure alignment between the analytic approach described and the procedures implemented in the study. The change is seen in the Track change document, pages 11 and 12, lines 227-252.

**Comment 2**: Some thematic sub-headings may also require refinement to better reflect the participant narratives and analytic interpretation of the findings.

**Response**: Thank you for this guidance. As guided in the attachment shared with the reviewer’s comments, the thematic sub-headings were refined. The included: ‘Dependence on school-based blood donation cycles’, ‘Scarcity of certain blood groups’, ‘Barriers to adult participation in blood donation’, ‘Perceived barriers to voluntary blood donation’, ‘Perceived turnaround time and operational constraints affecting blood and component preparation’, ‘Centrifuge breakdown and prolonged equipment downtime affecting component preparation’, ‘Fears and misconceptions about blood transfusion’, ‘Religious and cultural beliefs’, Fear associated with blood transfusion’, and ‘Complementary and supportive therapeutic practices alongside blood transfusion’. These revised thematic headings and sub-headings are found in the revised version of the manuscript. Thank you so much.

**Comment 3**: In addition, certain sections of the discussion would benefit from a more interpretive qualitative framing rather than strongly corrective biomedical explanations of participant beliefs and perceptions. A few statements may also require more cautious wording to avoid overgeneralization beyond the scope of the qualitative findings. Minor revisions are further needed to improve clarity, consistency of terminology, and reference formatting.

**Response**: Thank you for this helpful comment. We agree that certain sections of the Discussion section required a more consistently interpretive qualitative framing, as well as more cautious wording to avoid overgeneralization. We have carefully revised the Discussion accordingly, shifting from strongly corrective biomedical explanations toward a more interpretive presentation of participants’ beliefs and perceptions, in line with qualitative methodology.

Additionally, we have moderated statements that could be interpreted as overgeneralizing beyond the scope of the study findings, ensuring that interpretations remain grounded in the data. In addition, we have addressed the minor editorial issues highlighted in the annotated manuscript, including improvements to clarity, consistency of terminology, and reference formatting throughout the revised text.

The revised Discussion section now reads: ‘This study explored the perceptions of blood availability, safety, and acceptability among caregivers and healthcare providers of patients with cancer at Mbarara Regional Referral Hospital in southwestern Uganda. From this study, the availability of blood was affected by school holidays, donor complacency, high demand, scarcity of O and RhD-negative blood groups, rainy seasons, and disease outbreaks. Caregivers generally viewed blood as safe amidst concerns of adverse events and infection risks. Blood transfusions were highly valued; however, cultural and religious beliefs hindered their acceptability. Thus, supportive medical therapies and complementary practices were used alongside blood transfusion, and in some instances when transfusion was declined or unavailable. Healthcare providers perceived the blood supply as fragile and largely reliant on institution-based donors, and it was characterized by marked seasonal fluctuations during school terms and recesses.

Overall, while both caregivers of patients with cancer and healthcare providers identified similar structural constraints affecting blood availability, differences were observed in how these challenges were framed. Caregivers of patients with cancer tended to emphasize cultural, religious and experiential concerns related to transfusions, whereas the healthcare providers more frequently highlighted systemic and supply-chain limitations within the blood transfusion system.

This perception is supported by data from UBTS: in 2022-2023, only 313,659 units were collected, satisfying approximately 70% of the country's requirements (21) and falling short of the WHO’s recommended minimum donation level of 1% of the population annually (22). The high dependency on educational institution-based donors reflects organized campaigns targeting students, a group perceived as a lower-risk population for TTIs (23), and aligns with patterns documented in low- and middle-income countries. In contrast, adult participation in voluntary blood donation was viewed as lower, mostly attributed to their competing socio-economic demands and, as some healthcare providers reported, limited motivation or engagement with blood donation initiatives. Additional challenges of blood availability were compounded by the perceived scarcity of O and RhD-negative blood groups, possibly due to a low donor-to-recipient ratio. As reported by previous studies (24–26), factors such as donor complacency and disease outbreaks remain a hindrance to blood collection. The above factors collectively were perceived to result in the scarcity of blood, undermining the healthcare provision by prolonging the waiting times and hospital stays. Moreover, the reports of ‘token of facilitation’ in the attempt to secure blood for the patients contravene the basis of altruism in blood banking. Further, private health facilities reportedly included ‘blood transfusion-related costs’ in their medical bills, likely reflecting charges for the transfusion services themselves rather than the cost of the blood unit. These reported practices were viewed to create a perception of commodification of blood and likely discourage sustainable participation by altruistically motivated donors, as previously reported (27). Further research is needed to better understand the nature, extent, and implications of these reported practices and to inform appropriate policy and regulatory responses. Given the challenges posed by the insufficiency of blood, there is a need for public awareness to address the persistent suboptimal annual blood collection in Uganda. More, although not explored in this study, avoidable transfusions create an imbalance between blood supply and demand, leading to unavailability for patients with critical needs (28). This study adds to the advocacy for the proper utilization of blood as a scarce and expensive resource.

Caregivers of patients with cancer perceived blood safety with ‘mixed reactions’. Although blood was highly regarded as safe, concerns about the associated risks emerged. These perceptions reflect awareness of potential risks, even in systems that adhere to WHO-recommended safety standards. The safety attributes are evidence of the rigorous pre-donation screening, laboratory testing, and quality assurance protocols, aligning perceptions with reality and reinforcing trust in the blood supply. On the other hand, caregivers expressed worries about infections and adverse events, similar to previous reports (7,9–11,17). The perceived safety concerns underscore the challenges caregivers may face in balancing the perceived risks of blood transfusion with its life-saving benefits (11).

Blood transfusion was generally acceptable; however, underlying expression of discontent revealed safety and appropriateness concerns. The enormous acceptance correlates with the existing data on the indispensable life-saving role of blood transfusion. For instance, in the United States, blood transfusion was regarded as “critical” and “of greatest importance” (29), similar to the findings in a study conducted at Ohio Hospital, where the benefits of blood transfusion were difficult to discern due to the patient’s disease condition (30). Notably, cultural and religious contempt of blood use was also reported, similar to previous studies (10,11,31), and may negatively influence transfusion uptake. Due to the limited availability, safety concerns, and objections to blood, participants reportedly used a range of complementary and traditional therapies. The role, effectiveness, and safety of these complementary and traditional practices remains complex and requires further research to safeguard patients’ health.

While blood transfusion is generally perceived as acceptable and life-saving, various perceptions identified in this study may undermine both its perceived safety and acceptability. For instance, some caregivers viewed transfusion as interventions reserved for the ‘very sick patients and as a last resort’, which may explain the misbelief that transfused patients were likely to die. This perception is likely shaped by the clinical context in which transfusions are frequently administered after alternative treatments have failed or when the patient’s condition is severe. Thus associating transfusion symbolically with impending death rather than recovery. Moreover, delayed consent or hesitation in such cases can compromise timely management of severe anemia or hemorrhagic shock, thereby increasing the risk of mortality. Therefore, the perceived association between transfusion and death likely reflects temporal proximity rather than causation, and hemovigilance data indicate that transfusion-attributable mortality is rare and usually linked to identifiable adverse events (32,33). Further, caregivers reported concerns about donors in the window period, during which infections may not be detectable, contributing to fears of TTIs. Healthcare providers also perceived risk from ‘walk-in’ donors seeking infectious disease testing through donation, while caregivers reported anecdotes of individuals testing HIV-negative before transfusion but positive afterwards. Although TTIs remain theoretically possible, rigorous donor screening has markedly reduced the risk. Additionally, the perceptions that transfused blood might originate from non-human sources or the transfer of a donor’s trait are incorrect. These perceptions suggest that blood may be understood not only as a biological substance but also as a carrier of identity, personal attributes, and social meaning. The findings highlight the importance of culturally sensitive educational and counselling interventions that address transfusion-related concerns while acknowledging the social and symbolic meanings attached to blood. Finally, although participants reported the use of animal blood or herbal remedies to support recovery, the role, effectiveness and safety of such practices in the management of severe or chemotherapy-induced anemia remain insufficiently understood and warrants further investigation. Of concern, in already resource-constrained settings, such unsupported perceptions may shape how patients and caregivers view blood transfusion and could influence transfusion-related decision-making. Addressing these concerns through culturally sensitive communication may help support informed decisions regarding transfusion within the broader context of the existing blood system challenges. The strengths of this study are highlighted by its dual focus on transfusion perceptions from both patients’ caregivers and healthcare providers in a context that has not been studied before in SSA. On the other hand, the study findings ought to be interpreted in light of the following: its single-center design and the fact that its results may be skewed towards the studied categories, excluding blood donors. Despite these limitations, the findings offer critical insights into the caregivers’ perception on blood availability, safety, and acceptability, which will guide future research.’ These changes are seen in the Track change document, pages 25, 26, 27, 28, and 29, lines 519-621.

6. PLOS authors have the option to publish the peer review history of their article ([what does this mean?](https://track.editorialmanager.com/CL0/https:%2F%2Fjournals.plos.org%2Fglobalpublichealth%2Fs%2Feditorial-and-peer-review-process%23loc-peer-review-history/1/010f019ec9cb2eb0-5349e38d-ae4c-4ba0-b403-5f5eeb34bfb6-000000/iBALsyh69ABr2hZD7W9svR6_pod6nkVp7B540fmjy50=258)). If published, this will include your full peer review and any attached files.
**Do you want your identity to be public for this peer review?** If you choose “no”, your identity will remain anonymous but your review may still be made public.

For information about this choice, including consent withdrawal, please see our [Privacy Policy](https://track.editorialmanager.com/CL0/https:%2F%2Fwww.plos.org%2Fprivacy-policy/1/010f019ec9cb2eb0-5349e38d-ae4c-4ba0-b403-5f5eeb34bfb6-000000/IXp14z5cVnkvKjVnQrxfWbOLnc-ph1snk7H7XSVH8-w=258).

Reviewer #1: **Yes:** Clara Abou Samra

Reviewer #2: **Yes:** Adekogbe Adebola Sunday

 [NOTE: If reviewer comments were submitted as an attachment file, they will be attached to this email and accessible via the submission site. Please log into your account, locate the manuscript record, and check for the action link "View Attachments". If this link does not appear, there are no attachment files.]

**Figure Resubmissions**:
While revising your submission, we strongly recommend that you use PLOS’s NAAS tool (https://ngplosjournals.pagemajik.ai/artanalysis) to test your figure files. NAAS can convert your figure files to the TIFF file type and meet basic requirements (such as print size, resolution), or provide you with a report on issues that do not meet our requirements and that NAAS cannot fix.

After uploading your figures to PLOS’s NAAS tool - https://ngplosjournals.pagemajik.ai/artanalysis, NAAS will process the files provided and display the results in the "Uploaded Files" section of the page as the processing is complete. If the uploaded figures meet our requirements (or NAAS is able to fix the files to meet our requirements), the figure will be marked as "fixed" above. If NAAS is unable to fix the files, a red "failed" label will appear above. When NAAS has confirmed that the figure files meet our requirements, please download the file via the download option, and include these NAAS processed figure files when submitting your revised manuscript.

**Response**: Thank you for this guidance. We confirm that the manuscript does not contain any figures; therefore, no figure files required processing through the NAAS tool.

**Corrections made in the manuscript**:

**Comment 1**: In the Abstract, page 4, lines 80-82, the original sentence wording: ‘These findings show that caregivers experienced persistent shortages in blood availability in Uganda, while transfusion acceptance for patients with cancer was driven by system-level and sociocultural constraints.’ The specific reviewer comment is that: ‘Consider replacing the above with: ‘These findings reveal persistent blood shortages, with transfusion acceptance influenced by system-level and sociocultural constraints.’

**Response**: Thank you for the guidance. The initial sentence has been revised, and it now reads: “These findings reveal persistent blood shortages, with transfusion acceptance influenced by system-level and sociocultural constraints.” The change is seen in the Track change document, page 4, lines 80-82.

**Comments 2**: In the Introduction section, lines 92-94, the initial sentence: ‘Of concern, frequent blood transfusions among patients with cancer are undermined by the documented risks (5,6).’ The reviewer comment is that: ‘replace the words undermined with complicated, and risks with clinical and perceived risks.’

**Responses**: The change has been made and the revised sentence now reads: “Of concern, frequent blood transfusions among patients with cancer are complicated by the documented clinical and perceived risks (5,6).” The change is seen in the Track change document, page 5, lines 92-94.

**Comment 3**: In the Introduction section, line 101, an additional statement on justification for the study leading to the aim statement to be considered. The specific reviewer comment is that: ‘add a statement on justification for the study leading to the aim statement.’

**Response**: Thank you for this advice. A correction has been made, and the additional statement reads: “However, evidence on caregivers’ and healthcare providers’ perceptions of blood transfusion in cancer care remains limited in Uganda. Understanding these perspectives is essential for identifying barriers to optimal transfusion care as well as informing strategies to strengthen transfusion services in cancer settings.” This additional statement is found in the Track change document, page 5, lines 102-105.

**Comment 4**: In the Materials and Methods section, specifically sub-section titled: ‘*study design, duration, and sites*’, a revision in the sentence is required to replace the word ‘considering’.

**Response**: We appreciate the guidance, and the revised sentence now reads: “This qualitative study used an instrumental case study approach, examining Mbarara Regional Blood Bank (MRBB) and Mbarara Regional Referral Hospital (MRRH) as an interconnected blood transfusion unit, from donor to recipient. The case was defined as the full transfusion pathway linking the two institutions, encompassing blood donation, processing, distribution, clinical decision-making, and transfusion, and was geographically and functionally bound to these sites. The case study framework informed purposive sampling across key points in the transfusion pathway, guided data collection across both institutions, and supported thematic analysis and interpretation of the findings within the bounded system.” The revision is found in the Track change document, page 6, lines 110-117.

**Comment 5**: In the Materials and Methods section, specifically sub-section titled: ‘*study participants and size*’, a revision is required to the sentence: ‘The sample size was guided by achieving data saturation (15,16).’

**Response**: Thank you so much for this comment. A correction has been made and in the revised sentence, the word ‘achieving’ has been replaced with ‘the principle of, and the corrected sentence now reads: “The sample size was guided by the principle of data saturation (15,16).” This change is found in the Track change document, page 7, line 138.

**Comment 6**: In the Materials and Methods section, specifically sub-section titled: ‘*study participants and size*’. The table included (Table 1: Characteristics of participants) shows a description of in-depth interviews as: ‘Adult caregivers (parents, spouse, or child) of patients with cancer.’

**Table 1: Characteristics of participants**

| Interview type | Description | Number of interviewees per site (category by gender) | |
| --- | --- | --- | --- |
|  |  | MRRH | MRBB |
| In-depth Interviews | Adult caregivers (parents, spouse, or child) of patients with cancer | 16 (11 Females, 5 Males | Not Applicable |
|  | Healthcare providers | 8 (3 Females, 5 Males) | 3 (1 Female, 2 Males) |
| Key Informant Interviews | Health care providers with the oversight responsibility | 2 (1 Female, 1 Male) | 1 Male |
| Total |  | 26 (15 Females, 11 Males) | 4 (1 Female, 3 Males) |

The reviewer’s comment is: ‘A child among the adult caregiver? Kindly re-confirm the child’s age and frame appropriately.’

**Response**: We appreciate this important comment. In the description, “Adult caregivers (parents, spouse, or child) of patients with cancer,” the term “child” was intended to refer to an adult son or daughter caring for a parent with cancer. The term described the caregiver’s relationship to the patient rather than the caregiver’s age. We confirm that all study participants were adults. In light of the reviewer’s comment, we agree that the term “child” could be interpreted as referring to a minor. To avoid this ambiguity, we have revised Table 1 by replacing “child” with “adult child (son/daughter).” This revision is found in the Track change document (Table 1), page 8.

**Comment 7**: In the Materials and Methods section, specifically sub-section titled: ‘*participant enrollment*’, the reviewer raises this concern: ‘Are all cancer patients adults? If there are minors among them, it would be great to know whether the caregiver are their parents or not because that may have implications on given “verbal consent” for their medical records to be released and for caregiver participation in the study. This should be reworded appropriately to protect ethical integrity.’

**Response:** Thank you for this critical observation. We can confirm that not all patients with cancer in our study were adults. Three caregivers were recruited from the pediatric oncology ward taking care of children, and one of these caregivers was not the child’s parent. We agree that the initial wording did not adequately distinguish between procedures taken for adult and pediatric patients. For adult patients, verbal consent was obtained from the patient before disclosing their diagnosis and the transfusion history to the research team. For pediatric patients, permission was obtained from the parents or legal guardians, with assent obtained from a child if aged ≥8years in accordance with the approved ethics procedure. we have also clarified that informed consent for study participation was obtained from the caregiver who was recruited for the interview. We have now revised the manuscript to clarify this.

The revised section of the manuscript now reads: “The treating medical officer identified the eligible patients with a cancer diagnosis who had previously received blood transfusions. For adult patients, the medical officer informed them about a study seeking to recruit their caregivers and obtained verbal consent to disclose the patient’s diagnosis and transfusion history to the research team for purposes of the caregiver recruitment. For pediatric patients, consent was obtained from the parent or legally authorized guardian, and assent was obtained from the stable child if the age was ≥8 in accordance with approved ethical procedures. This verbal consent or assent was documented in the recruitment study log and the procedure was approved by the Mbarara University of Science and Technology Research Ethics Committee. Following permission, a designated member of the research team met with the patient and their caregiver in privacy, provided detailed study information, assessed caregiver eligibility, obtained written informed consent from the caregiver, and then scheduled the interview.” This change is found in the Track change document, on pages 8 and 9, lines 157-169. We thank you and hope that this rewording protects the ethical integrity.

**Comment 8**: In the Materials and Methods section, specifically sub-section titled: ‘Inclusion and exclusion criteria’, the first sentence reads: ‘Caregivers were eligible if they were aged ≥18 years, had provided care to a patient with cancer for at least 1 month, were aware that the patient had been diagnosed with cancer, and the patient had received at least two blood transfusions in the past 3 months, including a transfusion during the current hospitalization as part of their clinical care.’ The reviewer has raised this comment: ‘Is it clinically plausible for a patient who has been under care for only one month to already have received at least two blood transfusions within the past three months? The issue is the alignment between minimum caregiver exposure period (at least 1month), but within the last 3 months. If there are caregivers who have cared for patient clients for 1 month, how plausible is it that they have witnessed at least 2 transfusion history in 3 months? Consider re-framing the inclusion criteria appropriately.’

**Response**: Thank you for this insightful observation. We agree that the original wording could have implied a mismatch between the minimum caregiving period and the patient’s transfusion history. Our intention was not to require caregivers to have personally witnessed all transfusion episodes that occurred within the preceding 3months, but rather to recruit caregivers who were actively involved in the caring for the patients with recurrent transfusion needs and who were familiar with the patient’s recent transfusion experiences. To improve clarity, we have revised the inclusion criteria to explicitly state that caregivers were required to be involved in the care of a patient who had received atleast 2 blood transfusions within the previous three months, including one during the current hospitalization.

The revised section now reads: “Caregivers were eligible if they were aged ≥ 18 years, had provided care to a patient with cancer for at least 1 month, were aware that the patient had been diagnosed with cancer, and were caring for a patient who had received at least two blood transfusions in the past 3 months, including a transfusion during the current hospitalization as part of their clinical care. The 1 month caregiving period was considered sufficient to ensure familiarity with the patient’s ongoing care needs and recent transfusion experiences. ” We hope this now clarifies the situation. This change is found in the Track change document, on page 9, lines 171-176.

**Comment 9**: In the Materials and Methods section, specifically sub-section titled: ‘*inclusion and exclusion criteria*’, for the sentence: ‘We also excluded caregivers who had mental instability or if the patient died before the interview.’ the reviewer advises that: ‘consider replacing the statement with: ‘We excluded caregivers with mental instability and those whose patients died before the interview could be conducted.’

**Response**: The revised sentence reads: “We excluded caregivers with mental instability and those whose patient died before the interview could be conducted.” This correction is given in the Track change document, on page 9, lines 183-184.

**Comment 10**: In the Materials and Methods section, specifically sub-section titled: ‘*data collection tools and approach*’, for the sentence: ‘Data were collected using semi-structured in-depth interview and key informant interview (KII) topic guides developed in English from existing literature (7,9,11,17). The topic guides included open-ended questions to explore the perceptions of blood needs, availability of blood, safety concerns, acceptability, and alternatives to blood transfusion (Supplementary Material). IDIs with caregivers were conducted in commonly spoken local languages (Runyankore-Rukiiga, Runyoro-Rutoro, Rufumbiira, and Luganda), while interviews with healthcare providers were conducted in English.’ The reviewer has raised this concern: ‘The manuscript indicates that trained research assistants fluent in the local languages conducted the interviews. However, the authors may consider briefly clarifying whether the interview guides were formally translated or administered through interviewer interpretation, and how consistency of meaning across languages was maintained during data collection and transcription.’

**Response**: Thank you for this valuable suggestion. We agree that more detail regarding language procedures are required to strengthen the methods description. the in-depth interview guides were developed in English and administered in participants preferred languages by trained graduate research assistants fluent in the local languages. The research assistants were also trained on the intent and meaning of each question and used the agreed-upon terminology for key concepts to ensure consistency across the interviews. Audio recordings were then transcribed by the lead author (IMT) who is fluent in the local languages, and translated transcripts were reviewed by IMT to ensure accuracy and preservation of meaning. This clarification has been added in the manuscript and the revised section now reads: “Data were collected using semi-structured in-depth interview and key informant interview (KII) topic guides developed in English from existing literature (7,9,11,17). The topic guides included open-ended questions to explore the perceptions of blood needs, availability of blood, safety concerns, acceptability, and alternatives to blood transfusion (Supplementary Material). IDIs with caregivers were conducted in participants’ preferred commonly spoken local languages (Runyankore-Rukiiga, Runyoro-Rutoro, Rufumbiira, and Luganda) by trained graduate research assistants fluent in these languages. The interview guides were developed in English and administered through interviewer translation during the interviews. To maintain consistency across languages, the interviewers received training on the intent and meaning of each question and used the agreed-upon terminology for key concepts. On the other hand, interviews with healthcare providers were conducted in English. Each interview had a facilitator and a note-taker, who also managed audio recordings. Interviews were conducted in private rooms at the pediatric and adult wards at MRRH. Healthcare providers were interviewed at their convenience at workplaces. Interviews lasted about 40-60 minutes and were audio-recorded. Probing questions were used to clarify responses and explore emerging issues, allowing for an iterative approach to data collection. Interviews continued until thematic saturation was reached (18), and detailed field notes were taken. One of the authors (IMT) transcribed all interviews and verified transcript accuracy, as well as the preservation of meaning.” This correction is given in the Track change document, on page 10, lines 191-210.

**Comment 11**: In the Materials and Methods section, specifically sub-section titled: ‘data collection tools and approach’, for the sentence: ‘One of the authors (IMT) transcribed all interviews and verified transcript accuracy. Interviews conducted in local languages were independently translated into English by two other authors (AC and AS), and IMT reviewed translations to ensure fidelity and resolved discrepancies.’ The reviewer has raised this concern: ‘The procedures for transcription and translation are well described. However, the authors may consider briefly clarifying how participants confidentiality and anonymization were maintained during transcription, translation, and analysis.’

**Response**: We appreciate this helpful suggestion. We agree that clarification of confidentiality procedures would strengthen the methodological description. To this, we added a paragraph explaining that audio recordings and transcripts were assigned unique study identification codes, as well, participants names and other direct identifiers were removed during transcription and only authorized members of the research team had access to the study data. additionally, we have also clarified that de-identified transcripts were used during translation, coding and analysis to maintain participants confidentiality. The newly added paragraph reads like this: “To protect the participants confidentiality, audio recordings and transcripts were assigned unique study identification codes and did not contain participant names or any direct identifiers. Any potentially identifying information mentioned during the interview process (such as individuals and organizations) was removed from the transcription. More, only the authorized members of the research team had access to the audio recordings and transcripts, and de-identified transcripts were used for translation, transcription, coding as well as analysis.” This addition is given in the Track change document, on page 11, lines 213-218.

**Comment 12**:

In the Materials and Methods section, specifically sub-section titled: ‘data management and analysis’, for the sentence: ‘Qualitative data were analyzed using an inductive thematic approach. Three individuals (IMT, CA, and AS) independently coded the transcripts guided by the study objectives.’ The reviewer has raised this concern: ‘The authors may consider clarifying the relationship between the stated inductive thematic approach and coding guided by the study objectives, as this appears to combine inductive and deductive elements. Little discussion on how interpretive bias was managed can also be included to strengthen methodological rigor.’

**Response**: We agree that the original wording could be interpreted as combining inductive and deductive approaches without sufficient clarification. Our aim was to describe an inductive thematic analysis in which the study objectives informed the broad focus of inquiry, while codes and the themes were generated from the data rather than from a predefined coding framework. To address the gap in the initial write-up, we have revised the manuscript to clarify this analytical approach. We have now added information describing how interpretive bias was managed through independent coding by the three members of the team, regular consensus meetings, re-reading the relevant sections when discrepancies arose and iterative refinement of the codebook until agreement was achieved. These procedures were used to enhance the rigor and trustworthiness of the analysis.

The revised section of the manuscript now reads: “Qualitative data were analyzed using an inductive, team based thematic approach incorporating codebook development and iterative consensus coding. While the analysis was informed by the study objectives, no predetermined coding framework was applied. Instead, codes were generated from the data through repeated reading of transcripts and identification of concepts expressed by the participants. Three individuals (IMT, CA, and AS) independently reviewed and coded the transcripts. An initial codebook was developed using conventional content analysis after the coders independently reviewed the first six transcripts and was iteratively refined as new codes and themes emerged during analysis.

The coding process involved a line-by-line review of each transcript to identify patterns, relationships, and emerging themes. To enhance analytic rigor and minimize bias, three members of the research team (IMT, CA, and AS) independently coded transcripts and compared interpretations through regular consensus meetings. Discrepancies were resolved through discussion and re-reading the relevant sections of the transcripts together, and agreed on the best-fit interpretation of the data. Further analysis involved grouping similar codes to develop themes, which were refined through iterative team discussion.” This correction is given in the Track change document, on pages 11 and 12, lines 227-242.

**Comment 13**:

In the Materials and Methods section, specifically sub-section titled: ‘*data management and analysis*’, for the sentence: ‘In reporting the findings, “most participants” refers to over 75%, “some participants” refers to 25-75%, and “a few participants” refers to less than 25%.’ The reviewer has raised this concern: ‘The use of qualitative frequency descriptors such as “most”, “some”, and “a few” is acceptable; however, the authors may consider whether assigning rigid percentage thresholds to these terms is necessary, as this may unintentionally imply quantitative interpretation of qualitative findings.’

**Response**: We are grateful for this critical comment. We agree that assigning fixed percentages threshold to qualitative frequency descriptions may inadvertently suggest a quantitative interpretation that is not consistent with the inductive thematic approach as used in this study. As our intention was to provide readers with a general sense of the relative prevalence of views across interviews rather than to quantify participants responses, we have revised the manuscript by removing the percentage thresholds and clarifying that terms such as “most participants,” “some participants,” and “a few participants” are used descriptively to indicate the relative prevalence of perspectives without implying statistical measurement.

The revised sentence now reads: ‘In reporting the findings, qualitative frequency descriptions such as “most participants” “some participants” and “a few participants” were used to indicate the relative prevalence of views across interviews without implying statistical quantification.’ This correction is given in the Track change document, on page 12, lines 249-252.

**Comment 14**:

In the Materials and Methods section, specifically sub-section titled: ‘ethical considerations’, for the sentence: ‘They authorized the audio recording and the publication of anonymized responses.’ The reviewer has raised this concern: ‘It sounds like participants approved publication itself, rather than consented to the use of anonymized quotations/data during publication. Kindly reword appropriately.’

**Response**: Thank you for this observation. We admit that the initial wording could be interpreted as suggesting that participants authorized publication of the study itself rather than consented to the use of their data. in light of this, we have revised the sentence to clarify that: ‘Participants consented to audio recording of the interviews and to the use of anonymized data and quotes in research reports and publications.’ We hope that this wording accurately reflects the aspect of the informed consent and ethical use of participant data. we apologize for the language initially used. This correction is given in the Track change document, on pages 12 and 13, lines 258-260.

**Comment 15**:

In the Materials and Methods section, specifically sub-section titled: ‘ethical considerations’, for the sentence: ‘As caring for patients with cancer is associated with psychosocial distress, the study offered psychosocial support to participants through the psychiatry unit at MRRH.’ The reviewer has raised this concern: ‘the study offered psychosocial support to participants through the psychiatry unit at MRRH: suggests you replace with participants requiring emotional support were referred to the psychiatry unit at MRRH for psychosocial support services’ .

**Response**: Thank you for this guidance. We have revised the sentence to clarify that: ‘Participants requiring emotional or psychological support were referred to the psychiatry unit at MRRH for appropriate psychosocial support services.’ This correction is given in the Track change document, on page 13, lines 263-265.

**Comment 16**:

In the Results section, specifically sub-section titled: ‘*qualitative findings: themes*’ the reviewer advises that: ‘The authors should consider adding an orienting statement or some sort of introduction before presenting the themes.’

**Response**: We are grateful to this advice. We have added a brief orienting paragraph at the beginning of the “Qualitative findings: Themes” section to introduce the thematic structure of the results, summarize the themes identified during analysis, and provide context for the sub-themes. The introductory sentence reads: ‘Qualitative analysis of interviews with the caregivers of patients with cancer and healthcare providers identified three overarching themes relating to blood transfusion in the study setting: perceptions of blood availability, perceptions of blood safety, and acceptability of blood transfusion. These themes captured participants’ experiences and perceptions regarding access to blood and blood components, confidence in the transfusion safety, and factors influencing the acceptance or refusal of blood transfusion. More, each theme comprised several sub-themes that reflected the complexity of blood transfusion practices with in the studied setting. Illustrative quotations are presented to support the findings.’ This correction is given in the Track change document, on page 14, lines 273-280.

**Comment 17**:

In the Results section, specifically sub-section titled: ‘*sub-theme 1: perceived barriers to blood supply*’ the reviewer advises to ‘kindly reconsider changing the sub-topic school calendar dependence to something more appropriate for example as a guide this appears to be ‘dependence on school-based donation cycle which peaked and dropped based on school sessions’.

**Response**: Thank you for this guidance. We agree that the initial sub-theme title did not fully capture the underlying issue reflected in the participants’ accounts. The concern described by participants relates to the heavy reliance on school-going donors and the resulting fluctuations in blood collection across school terms and holidays. In light of this, we have revised the sub-theme title to ‘Dependence on school-based blood donation cycles’ to more accurately reflect the findings and improve clarity. This correction is given in the Track change document, on page 14, line 284.

**Comment 18**: In the Results section, specifically sub-section titled: ‘*sub-theme 1: perceived barriers to blood supply*’ the reviewer advises to ‘The authors may consider using more natural academic phrasing such as “students” instead of “learners” in educational institutions.’

**Response**: A revision to reflect ‘students’ instead of ‘learners’ has been made. This correction is given in the Track change document, on page 14, line 285.

**Comment 19**: For quotes throughout the result section such as: *“Over 80% of all the donated blood comes from school-going donors, and the availability of blood depends on school time. ..during the school term, we collect≥100 units, but this drops to < 20 units during the holidays.” IDI-Healthcare Provider-7; Male*

The reviewer has ‘suggested replacement to make it more readable *(IDI, Healthcare Provider 7, Male)*

**Response**: Thank you for the guidance. The correction (such as IDI, Healthcare Provider 7, Male) has been made all through the results section.

**Comment 20**: In the Results section, specifically sub-section titled: ‘*sub-theme 1: perceived barriers to blood supply*’ the reviewer advises to ‘The author should consider replacing the initial sub-heading: Scarcity of some blood group donation with Scarcity of certain blood groups.’

**Response**: The correction to the sub-heading has been made to reflect it as: ‘Scarcity of certain blood groups.’ This correction is given in the Track change document, on page 15, line 292.

**Comments 21**: In the Results section, specifically sub-section titled: ‘sub-theme 1: perceived barriers to blood supply’ under the subheading: ‘Incentive expectations undermining altruistic blood donation,’ the reviewer advises: ‘The authors to consider rewording the underlined section of the sentence ‘A healthcare provider from UBTS reported that some donors thought that blood transfusion should be associated with costly incentives, as expressed. This rewording should aim to convey the intended meaning.

Also, to clarify if the underlined means that some donors expected material incentives or benefits in exchange for donating blood?’

**Responses**: Thank you for this important clarification. We confirm that the intended meaning was that some donors expected material incentives or benefits in exchange for donating blood rather than engaging in voluntary, non-remunerated donation. To this, we have revised the sentence to provide clarity as shown: ‘A healthcare provider from UBTS reported that some donors expected material incentives or benefits in exchange for donating blood, including tangible rewards such as bicycles or assurances of free medical services, as expressed;’ This correction is given in the Track change document, on page 15, lines 299-301.

Also, to clarify that ‘yes’, some donors expected material incentives or benefits in exchange for donating blood.

**Comments 22**: In the Results section, specifically sub-section titled: ‘*sub-theme 1: perceived barriers to blood supply*’ under the subheading: ‘Low adult participation in blood donation programs due to lifestyle and attitude,’ the reviewer advises that: ‘The sub will benefit from rephrasing. For example: Barriers to adult participation in blood donation.’

In addition, in the same section, the reviewer indicates that: ‘The narrative introducing the quotation may benefit from minor rephrasing to improve clarity and reducing ambiguity regarding whose sentiments are being described. For example: ‘A healthcare provider from UBTS perceived adult participation in blood donation to be low due to daily life commitments. Some healthcare providers also expressed negative sentiments regarding adults’ willingness to donate blood.’

**Responses**: Thank you for your guidance. We confirm that the sub-heading has been revised to: ‘Barriers to adult participation in blood donation.’ This change is given in the Track change document, on page 15, line 306.

Also, a correction to the narrative introducing the quotation has been revised to clearly attribute perceptions to healthcare providers, particularly those from UBTS and to improve clarity regarding the source of the expressed sentiments. The revised narrative reads: ‘A healthcare provider from UBTS perceived adult participation in blood donation to be low due to daily life commitments. Some of healthcare providers also expressed negative sentiments regarding adults’ willingness to donate blood, as quoted;’ This change is given in the Track change document, on pages 15 and 16, lines 307-310.

**Comment 23**: In the Results section, specifically sub-section titled: ‘*sub-theme 1: perceived barriers to blood supply*’ under the subheading: ‘Impact of adverse weather on donor turnout and blood collection,’ the reviewer advises that: ‘The author should consider rephrasing the concluding introduction to make it readable. For example ….challenging occasionally resulting in failed donations.’

**Response**: We are grateful for the critical support. We have revised the sentence for clarity and readability by restructuring it to improve flow and ensure clearer communication of the reported impact of cold weather conditions on donor turnout and the success of blood collection procedure. the revised section now reads: ‘Healthcare providers from UBTS indicated that adverse weather negatively affected donor turnout during field collections. They reported that cold conditions made phlebotomy more challenging and were occasionally associated with failed donation attempts.’ This change is given in the Track change document, on page 16, lines 315-318.

**Comments 24**: In the Results section, specifically sub-section titled: ‘*sub-theme 2: perceived inequities affecting blood availability*’ the reviewer comment is that: ‘The sub-theme sounded broader and more structural beyond respondent’s submission. The author should reconsider rephrasing the sub-theme. For example: ‘perceived barriers to voluntary blood donation.’

Also, in the introductory statement: “Some caregivers and healthcare providers expressed concerns about blood donation as a key barrier to voluntary donation. They stated that communities sometimes held negative attitudes toward the donation process, influenced by a perceived sense of ‘unmatched loyalty’. While blood donation is voluntary and non-remunerated, caregivers and healthcare providers reported that concerns about transfusion-related ‘charges’ incurred in healthcare facilities contributed to apprehension.” The reviewer advices that: ‘The phrase unmatched loyalty may benefit from clarification or contextual explanation as its meaning may not be immediately clear to readers.’ More, the reviewer recommends that we consider replacement of ‘charges’ with ‘expenses.’

**Responses**: We are grateful for these insightful suggestions. We agree that the initial sub-theme title was broader than the participants’ accounts and have now been revised to: ‘Perceived barriers to voluntary donation.’ This change is given in the Track change document, on page 16, line 325.

In addition, we have replaced the word ‘charges’ to ‘expenses’, and the revised section now reads: ‘Some caregivers and healthcare providers expressed concerns that acted as barriers to voluntary blood donation. They reported that communities sometimes held negative attitudes toward the donation process, partly influenced by a perceived lack of reciprocity between blood donation and access to transfusion services. While blood donation is voluntary and non-remunerated, caregivers and healthcare providers reported that concerns about transfusion-related expenses incurred in healthcare facilities contributed to apprehension about donating blood, as expressed;’ This change is given in the Track change document, on pages 16 and 17, lines 327-333.

**Comment 25**: In the Results section, specifically sub-section titled: ‘*sub-theme 3: perceived operational limitations to blood and component availability*’ the reviewer comment is that: ‘The authors to consider re-labelling as appropriate. The authors should think around delay TAT (turn around time) between sample collection and processing during field visits.’

**Response**: Thank you for this comment. We agree that the original sub-theme title did not adequately reflect the operational concerns described by participants. To this, we have revised the title to: ‘Perceived turnaround time and operational constraints affecting blood component preparation.’ We believe that this revision captures the reported delays between field blood collection and processing at the blood bank, and the equipment-related challenges that affected timely preparation of blood components like platelets and fresh frozen plasma. This change is given in the Track change document, on page 17, lines 340-341.

**Comment 26**: In the Results section, specifically sub-section titled: ‘*sub-theme 3: perceived operational limitations to blood and component availability*’ sub-heading ‘Operational challenges undermining access to timely component availability’, the reviewer comment is that: ‘The operational challenges is too general however it was defined around breakdown and equipment downtime. Kindly consider rephrasing to something more specific as indicated in the finding.’

**Response**: We are grateful for your guidance. We acknowledge that the phrase ‘operational challenges’ was too broad and didn’t adequately reflect the specific issue reported in the findings. to address this, we have revised the sub-heading to: ‘Centrifuge breakdown and prolonged equipment downtime affecting component preparation’ . This change is given in the Track change document, on page 17, lines 350-351.

**Comments 27**: In the Results section, specifically sub-section titled: ‘*sub-theme 2: concerns of blood transfusions*’ the reviewer comment is that: ‘The authors may consider a more analytically appropriate sub-theme for this section, as the findings appear to reflect fear and misconceptions surrounding blood transfusion rather than general concerns alone. For example, a sub-theme such as Fears and misconceptions about blood transfusion may better capture the underlying perceptions expressed by participants.’

Also, under the same section, a sub-title: ‘Religious and cultural concerns,’ the reviewer comment is that: ‘Consider using beliefs instead of “concerns” as the findings appear to reflect sociocultural and religious beliefs rather than concerns alone.

**Responses**: Thank you for the insightful comments. We have revised the sub-theme 2 title to: ‘Fears and misconceptions about blood transfusion.’ This change is given in the Track change document, on page 22, line 463.

Also, the initial sub-title: ‘Religious and cultural concerns’ has been revised to: ‘Religious and cultural beliefs.’ This change is given in the Track change document, on page 22, line 464.

**Comment 28**: In the Results section, specifically sub-section titled: ‘sub-theme 2: Relating transfusion to end-of-life care’ the reviewer comment is that: ‘The title may be better framed as “Fear associated with blood transfusion.”’

**Response**: Thank you for this guidance. The sub-heading that was initially titled as: ‘Relating transfusion to end-of-life care’ has now been revised to: ‘Fear associated with blood transfusion.’ This change is given in the Track change document, on page 23, line 483.

**Comment 29**: In the Results section, specifically sub-section titled: ‘sub-theme 3: Alternatives to blood transfusion’ the reviewer comment is that: ‘The authors may consider revising the sub-theme title alternatives to transfusion, as several of the described practices appear to function as complementary or supportive therapies rather than true alternatives to transfusion. A title reflecting complementary, supportive, or co-administered therapeutic approach may better align with the findings described in supportive, co-administered therapeutic approaches may better align with the findings described in the narrative.

**Response**: Thank you so much for this critical observation. We agree that the original title: Alternatives to blood transfusion’ may incorrectly suggest substitution of transfusion while the findings reflects adjunct and co-administered practices. To address this, we have revised the title to: ‘Complementary and supportive therapeutic practices alongside blood transfusion.’ We finds this more aligned with the data, and reflects both the biomedical supportive treatments prescribed by healthcare providers and traditional or dietary practices used by caregivers in conjunction with blood transfusion. This change is given in the Track change document, on page 23, lines 488-489.

**Comment 30**: In the Discussion section, specifically paragraph one: ‘This study explored the perceptions of blood availability, safety, and acceptability among caregivers and healthcare providers of patients with cancer at Mbarara Regional Referral Hospital in southwestern Uganda. From this study, the availability of blood was affected by school holidays, donor complacency, high demand, scarcity of O and RhD-negative blood groups, rainy seasons, and disease outbreaks. Caregivers generally viewed blood as safe amidst concerns of adverse events and infection risks. Blood transfusions were highly valued; however, cultural and religious beliefs hindered their acceptability. Thus, alternatives such as medicines and complementary therapies were used.’ The reviewer recommends is that: ‘The author should consider replacing this (the underlined word--alternatives) as drugs and other things mentioned are not necessarily alternatives but complementary.

**Response**: We are grateful to the reviewer for this comment. We agree that the term initially used ‘alternatives’ could imply replacement of blood transfusion, while out findings indicate that medicines and traditional remedies were commonly used as supportive or complementary approaches often alongside transfusion rather than in place of it. To this, we have revised the specific sentence to reflect this distinction. The revised sentence now reads: ‘Thus, supportive medical therapies and complementary practices were used alongside blood transfusion, and in some instances when transfusion was declined or unavailable.’ This change is given in the Track change document, on page 25, lines 525-527.

**Comment 31**: In the Discussion section, the reviewer advised to revisit the underlined words in this sentence: ‘In contrast, adult participation in voluntary blood donation was viewed as lower, mostly attributed to their socio-economic demands and, as some healthcare providers reported, a lax attitude.’ The specific review comment is: ‘The authors may consider revising terms such as ‘lax attitude’ to more neutral language that better reflects participant perceptions without implying judgement.’

**Response**: We are grateful for this important highlight. We admit that the initial expression ‘lax attitude’ may be perceived as judgmental and could imply endorsement of participants’ views. To maintain neutrality and accurately reflect the qualitative findings, we have revised the sentence appropriately. This section now reads: ‘In contrast, adult participation in voluntary blood donation was viewed as lower, mostly attributed to their competing socio-economic demands and, as some healthcare providers reported, limited motivation or engagement with blood donation initiatives.’ This change is given in the Track change document, on page 26, lines 540-543.

**Comment 32**: In the Discussion section, the reviewer advised to revisit the underlined word in this sentence: ‘These practices were viewed to create a perception of commodification of blood and likely discourage sustainable participation by altruistically motivated donors, as previously reported (28). The reported malpractices require further research to inform the punitive measures. Given the challenges posed by the insufficiency of blood, there is a need for public awareness to address the persistent suboptimal annual blood collection in Uganda.’ The specific review comment is: ‘The term “malpractices” may be reconsidered or more cautiously framed, as the findings appear to reflect participant reports and perceptions rather than independently verified practices.’

**Response**: We are grateful to the reviewer for this important observation. We acknowledge the fact that the term ‘malpractices’ may imply independently verified wrongdoing, whereas our findings are based on participants reports and perceptions. To this, we have revised the sentence to ‘reported practices’ rather than ‘malpractices’. The revised sentence now reads: ‘Moreover, the reports of ‘token of facilitation’ in the attempt to secure blood for the patients contravene the basis of altruism in blood banking. Further, private health facilities reportedly included ‘blood transfusion-related costs’ in their medical bills, likely reflecting charges for the transfusion services themselves rather than the cost of the blood unit. These reported practices were viewed to create a perception of commodification of blood and likely discourage sustainable participation by altruistically motivated donors, as previously reported (27). Further research is needed to better understand the nature, extent, and implications of these reported practices and to inform appropriate policy and regulatory responses.’ This change is given in the Track change document, on page 26, lines 548-556.

**Comment 33**: In the Discussion section, the reviewer advised to revisit the underlined words in these sentences: ‘Notably, cultural and religious contempt of blood use was also reported, similar to previous studies (10,11,32), and may negatively influence transfusion uptake. Due to the limited availability, safety concerns, and objections to blood, the use of alternative therapy was reported. The prospect of alternative therapy remains complex and requires further research to safeguard patients' health. The specific review comment is: ‘The discussion may benefit from clearer distinction between complementary therapies and true alternatives to blood transfusion, particularly given that several practices were reportedly co-administered alongside transfusion rather than used substitutes.’

**Response**: We thank the reviewer for this critical observation. We agree that the term ‘alternative therapy’ may not accurately reflect the range of practices reported by participants. Our findings showed that many traditional and supportive therapies were used alongside blood transfusion rather than as direct substitutes, although some where reportedly used when transfusion was declined or unavailable. To this, we have revised this section of the discussion to reflect the correct and appropriate finding. The revised sentence now reads: ‘Notably, cultural and religious contempt of blood use was also reported, similar to previous studies (10,11,31), and may negatively influence transfusion uptake. Due to the limited availability, safety concerns, and objections to blood, participants reportedly using a range of complementary and traditional therapies. The role, effectiveness, and safety of these complementary and traditional practices remains complex and requires further research to safeguard patients’ health.’ This change is given in the Track change document, on pages 27 and 28, lines 576-582.

**Comments 34**: In the Discussion section, the reviewer advised to revisit the underlined words in these sentences: ‘Healthcare providers also perceived risk from ‘walk-in’ donors seeking infectious disease testing through donation, while caregivers reported anecdotes of individuals testing HIV-negative before transfusion but positive afterwards. Although TTIs remain theoretically possible, rigorous donor screening has markedly reduced the risk. Additionally, the perceptions that transfused blood was from non-human sources or the transfer of a donor’s trait are incorrect, as xenogeneic transfusion is not practiced due to immunological incompatibility, and red blood cells function solely in oxygen delivery. Finally, animal blood or herbal remedies can not physiologically substitute for transfusion in severe or chemotherapy-induced anemia. The specific review comment is: ‘The discussion provides important biomedical clarification regarding transfusion misconceptions. This may benefit from a more interpretive qualitative framing rather than directly correcting participant beliefs.’

Also, another sentence is: ‘Of concern, in already resource-constrained settings, such unsupported perceptions may delay consent, reduce transfusion uptake, and exacerbate avoidable morbidity, thereby compounding genuine structural challenges within blood systems.’ The specific review comment is: ‘There is an overgeneralization risk here as the study did not measure delay consent, reduce transfusion uptake and morbidity. The authors should word these implications cautiously.’

**Responses**: We thank the reviewer for this insightful comment. We agree that directly characterizing participants beliefs as ‘incorrect’ may be inconsistent with the interpretive aims of qualitative research. We have revised the sentence to focus on the social and symbolic meanings that participants attached to blood and how these perceptions may influence transfusion acceptability. The revised sentence now reads: ‘Additionally, the perceptions that transfused blood might originate from non-human sources or the transfer of a donor’s trait are incorrect. These perceptions suggest that blood may be understood not only as a biological substance but also as a carrier of identity, personal attributes, and social meaning. The findings highlights the importance of culturally sensitive educational and counselling interventions that address transfusion-related concerns while acknowledging the social and symbolic meanings attached to blood. Finally, although participants reported the use of animal blood or herbal remedies to support recovery, the role, effectiveness and safety of such practices in the management of severe or chemotherapy-induced anemia remain insufficiently understood and warrants further investigation.’ This change is given in the Track change document, on pages 28 and 29, lines 599-609.

Also, we sincerely apologize and admit that our study did not directly assess delays in consent, transfusion uptake or morbidity outcomes. Thus, we have revised the sentence to avoid causal inferences and instead emphasized that such perceptions may influence attitude toward blood transfusion and transfusion-related decision-making. This revised wording more accurately reflects the scope of the data and the interpretive nature of our findings. The revised sentence now reads: ‘Of concern, in already resource-constrained settings, such unsupported perceptions may shape how patients and caregivers view blood transfusion and could influence transfusion-related decision-making. Addressing these concerns through culturally sensitive communication may help support informed decisions regarding transfusion within the broader context of the existing blood system challenges.’ This change is given in the Track change document, on page 29, lines 609-613.

**Comment 35**: In the Reference section, attention or clarity is required for reference number 19: ‘Byrne D. A worked example of Braun and Clarke’s approach to reflexive thematic analysis. Qual Quant. 2022;56(3):1391–412.’ The specific review comment is: ‘The manuscript cites literature related to Braun and Clarke’s reflective thematic analysis against the analytic procedures described which appears more aligned with a codebook or consensus-oriented thematic analysis approach. The author may consider refining/removing this reference or clarify the specific thematic analysis approach used.

**Response**: Thank you so much for this guidance. We agree that the original citation to Braun and Clarke’s reflexive thematic analysis (initially given as number 19) was not fully aligned with the analytical approach used. To this, we have deleted this reference, and revisions both in the ‘Materials and Methods’ section and ‘Reference’ list made. The in-text citations have all been updated accordingly.

We are very grateful to the editorial team and the reviewers for these critical corrections and guidance that have helped improve the manuscript, and we apologize for the oversight in the previous submission. We confirm that all revisions are highlighted in the ‘Track changes’ of the R1 manuscript. We look forward to your consideration and eventual publication of this manuscript.

Sincerely,

Ivan Mugisha Taremwa

+256774346368

[imugisha@ymail.com](mailto:imugisha@ymail.com)
