# Supplementary material for: Caregivers’ and healthcare providers’ perceptions of blood availability, safety, and acceptability for patients with cancer in southwestern Uganda
Source: PLOS Glob Public Health. 2026 Sep 11;6(9):e0007203. doi: 10.1371/journal.pgph.0007203 (PMC13567789; doi:10.1371/journal.pgph.0007203)
Supplement: Peer review history

# Peer review: Decision Letter 0

**DOI:** 10.1371/journal.pgph.0007203.r001

**Authors:** Ferdinand C Mukumbang

15 Jun 2026

PGPH-D-26-01258

Caregivers’ and healthcare providers’ perceptions of blood availability, safety, and acceptability for patients with cancer in southwestern Uganda.

PLOS Global Public Health

Dear Dr. Taremwa,

Thank you for submitting your manuscript to PLOS Global Public Health. After careful consideration, we feel that it has merit but does not fully meet PLOS Global Public Health’s publication criteria as it currently stands. Therefore, we invite you to submit a revised version of the manuscript that addresses the points raised during the review process.

The reviewers and I feel that the work is original and fills an important gap in understanding blood transfusion services among patients undergoing cancer treatment, and that, for the most part, the manuscript is well-written. To strengthen this submission, the reviewers have suggested that the authors pay more attention to the methods section. Specific attention was drawn to the nature of the case study design and the thematic analytic approach that were applied. I therefore invite the authors to consider the reviewers' comments and any other relevant editorial comments to strengthen the quality of their submission.

Please submit your revised manuscript by August 10, 2026. If you will need more time than this to complete your revisions, please reply to this message or contact the journal office at globalpubhealth@plos.org. When you're ready to submit your revision, log on to https://www.editorialmanager.com/pgph/ and select the 'Submissions Needing Revision' folder to locate your manuscript file.

Please include the following items when submitting your revised manuscript:

- A letter that responds to each point raised by the editor and reviewer(s). You should upload this letter as a separate file labeled 'Response to Reviewers'.
- A marked-up copy of your manuscript that highlights changes made to the original version. You should upload this as a separate file labeled 'Revised Manuscript with Track Changes'.
- An unmarked version of your revised paper without tracked changes. You should upload this as a separate file labeled 'Manuscript'.

Guidelines for resubmitting your figure files are available below the reviewer comments at the end of this letter.

As the corresponding author, your ORCID iD is verified in the submission system and will appear in the published article. PLOS supports the use of ORCID, and we encourage all coauthors to register for an ORCID iD and use it as well. Please encourage your coauthors to verify their ORCID iD within the submission system before final acceptance, as unverified ORCID iDs will not appear in the published article. *Only*  the individual author can complete the verification step; PLOS staff *cannot*  verify ORCID iDs on behalf of authors.

We look forward to receiving your revised manuscript.

Kind regards,

Ferdinand C Mukumbang, PhD

Academic Editor

PLOS Global Public Health

Journal Requirements:

1. In the ethics statement in the Methods, you have specified that verbal consent was obtained. Please provide additional details regarding how this consent was documented and witnessed, and state whether this was approved by the IRB.

2. Please provide a detailed online Financial Disclosure statement. This is published with the article. It must therefore be completed in full sentences and contain the exact wording you wish to be published.

a) State the initials, alongside each funding source, of each author to receive each grant. For example: “This work was supported by the National Institutes of Health (####### to AM; ###### to CJ) and the National Science Foundation (###### to AM).”

b) State what role the funders took in the study. If the funders had no role in your study, please state: “The funders had no role in study design, data collection and analysis, decision to publish, or preparation of the manuscript.”

For more information, please go to our submission guidelines:

https://journals.plos.org/globalpublichealth/s/submission-guidelines#loc-financial-disclosure-statement

3. Please ensure that the funders and grant numbers match between the Financial Disclosure field and the Funding Information tab in your submission form. Note that the funders must be provided in the same order in both places as well.

4. Please update your online Competing Interests statement. If you have no competing interests to declare, please state: "The authors have declared that no competing interests exist."

5. We note that your Data Availability Statement is currently as follows: "The relevant data used is available in the manuscript."

Please confirm at this time whether or not your submission contains all raw data required to replicate the results of your study. Authors must share the “minimal data set” for their submission. PLOS defines the minimal data set to consist of the data required to replicate all study findings reported in the article, as well as related metadata and methods (https://journals.plos.org/globalpublichealth/s/data-availability#loc-minimal-data-set-definition).

For example, authors should submit the following data:

- The values behind the means, standard deviations and other measures reported;

- The values used to build graphs;

- The points extracted from images for analysis.

Authors do not need to submit their entire data set if only a portion of the data was used in the reported study.

If your submission does not contain these data, please either upload them as Supporting Information files or deposit them to a stable, public repository and provide us with the relevant URLs, DOIs, or accession numbers. For a list of recommended repositories, please see https://journals.plos.org/globalpublichealth/s/recommended-repositories.

If there are ethical or legal restrictions on sharing a de-identified data set, please explain them in detail (e.g., data contain potentially sensitive information, data are owned by a third-party organization, etc.) and who has imposed them (e.g., an ethics committee). Please also provide contact information for a data access committee, ethics committee, or other institutional body to which data requests may be sent. If data are owned by a third party, please indicate how others may request data access.

6. "Supplementary Material.docx" is currently uploaded as an 'Other' file type, which is not viewable by reviewers. Please ensure that all files meant for review are uploaded as 'Supporting Information' and include a legend in the manuscript.

7. If the reviewer comments include a recommendation to cite specific previously published works, please review and evaluate these publications to determine whether they are relevant and should be cited. There is no requirement to cite these works unless the editor has indicated otherwise.

Additional Editor Comments (if provided):

[Note: HTML markup is below. Please do not edit.]

Reviewers' comments:

Reviewer's Responses to Questions

**Comments to the Author**

1. Does this manuscript meet PLOS Global Public Health’s publication criteria?

Reviewer #1: Partly

Reviewer #2: Yes

**********

2. Has the statistical analysis been performed appropriately and rigorously?

Reviewer #1: Yes

Reviewer #2: N/A

**********

3. Have the authors made all data underlying the findings in their manuscript fully available (please refer to the Data Availability Statement at the start of the manuscript PDF file)?

The PLOS Data policy

Reviewer #1: No

Reviewer #2: Yes

**********

4. Is the manuscript presented in an intelligible fashion and written in standard English?

Reviewer #1: Yes

Reviewer #2: Yes

**********

Reviewer #1: This manuscript addresses an important and underexplored issue regarding perceptions of blood availability, safety, and acceptability among caregivers and healthcare providers of patients with cancer in Uganda. The topic is highly relevant to global public health, transfusion systems, and cancer care in low-resource settings. The manuscript is generally well structured, and the qualitative approach is appropriate for exploring complex sociocultural and system-level perceptions.

The study has several strengths, including inclusion of both caregivers and healthcare providers, use of in-depth interviews and key informant interviews, and attention to ethical considerations. The findings provide valuable insights into how structural shortages, sociocultural beliefs, and trust in healthcare systems shape transfusion-related perceptions and practices.

However, several areas require strengthening before the manuscript is suitable for publication.

First, the methodological framing as a “case study approach” requires further clarification. The authors should explain how the case boundaries were defined, which type of case study design was adopted, and how the case study framework informed sampling, analysis, and interpretation.

Second, the manuscript would benefit from stronger reporting of qualitative rigor and reflexivity. While coding procedures are described, there is limited discussion of researcher positionality, reflexive practice, credibility strategies, dependability, confirmability, or transferability. The authors should elaborate on how trustworthiness was ensured beyond coder discussions and transcript review. The authors should confirm if they followed the Consolidated Criteria for Reporting Qualitative Research (COREQ) to ensure all reporting standards for qualitative studies are met.

Third, some conclusions appear stronger than warranted by the qualitative data. Statements suggesting that misconceptions may exacerbate preventable mortality or references to “malpractices” requiring punitive measures should be presented more cautiously unless directly supported by the findings.

Fourth, the analysis could be deepened by exploring variations across participant groups. For example, sociocultural and religious perceptions may differ meaningfully across communities, caregiver categories, or healthcare provider roles, yet these distinctions are not explored analytically.

Fifth, the Data Availability Statement does not appear fully aligned with PLOS requirements. The statement that “relevant data used is available in the manuscript” is insufficiently detailed. The authors should clarify whether anonymized transcripts, coding frameworks, or supporting qualitative datasets are available, and if restrictions apply due to confidentiality, these should be explicitly stated.

Minor revisions are also recommended regarding language editing, grammar, spacing, and repetitive phrasing.

Overall, this manuscript addresses a highly important topic and has potential to contribute meaningfully to the literature on transfusion practices and cancer care in sub-Saharan Africa. With revisions to strengthen methodological transparency, qualitative rigor, and interpretive caution, the manuscript would be substantially improved.

Reviewer #2: Overall, the manuscript presents a qualitative exploration of perceptions surrounding blood availability, safety, and acceptability among caregivers and healthcare providers in southwestern Uganda. The study addresses an important clinical and operational gap in cancer care and transfusion services within resource-limited settings in sub-Saharan Africa. The qualitative design is appropriate for the study objectives, and the findings provide valuable insights into sociocultural beliefs, safety perceptions, blood availability challenges, and factors influencing transfusion acceptability.

The manuscript is generally well structured and demonstrates good integration of participant narratives with existing literature. The topic is highly relevant to strengthening transfusion services, cancer care, and patient-centered care in resource-limited settings.

However, several areas require clarification and refinement before the manuscript can fully meet the publication standards of PLOS ONE. Specifically, the authors should consider improving methodological clarity regarding the thematic analysis approach, particularly the alignment between the cited reflexive thematic analysis literature and the consensus/codebook-oriented analytic procedures described in the methods section. Some thematic sub-headings may also require refinement to better reflect the participant narratives and analytic interpretation of the findings.

In addition, certain sections of the discussion would benefit from a more interpretive qualitative framing rather than strongly corrective biomedical explanations of participant beliefs and perceptions. A few statements may also require more cautious wording to avoid overgeneralization beyond the scope of the qualitative findings. Minor revisions are further needed to improve clarity, consistency of terminology, and reference formatting.

**********

what does this mean?). If published, this will include your full peer review and any attached files.

**Do you want your identity to be public for this peer review?**  If you choose “no”, your identity will remain anonymous but your review may still be made public.

For information about this choice, including consent withdrawal, please see our Privacy Policy

Reviewer #1: **Yes:**  Clara Abou Samra

Reviewer #2: **Yes:**  Adekogbe Adebola Sunday

**********

[NOTE: If reviewer comments were submitted as an attachment file, they will be attached to this email and accessible via the submission site. Please log into your account, locate the manuscript record, and check for the action link "View Attachments". If this link does not appear, there are no attachment files.]

Attachment Submitted filename: PGPH-D-26-01258_reviewer.pdf

# Author comment: Author response to Decision Letter 1

**DOI:** 10.1371/journal.pgph.0007203.r002

21 Jun 2026

Attachment Submitted filename: Response Letter to the reviewers comments..docx

# Peer review: Decision Letter 1

**DOI:** 10.1371/journal.pgph.0007203.r003

**Authors:** Julia Robinson

12 Aug 2026

Caregivers’ and healthcare providers’ perceptions of blood availability, safety, and acceptability for patients with cancer in southwestern Uganda.

PGPH-D-26-01258R1

Dear Mr. Taremwa,

We are pleased to inform you that your manuscript 'Caregivers’ and healthcare providers’ perceptions of blood availability, safety, and acceptability for patients with cancer in southwestern Uganda.' has been provisionally accepted for publication in PLOS Global Public Health.

Before your manuscript can be formally accepted you will need to complete some formatting changes, which you will receive in a follow up email. A member of our team will be in touch with a set of requests.

Please note that your manuscript will not be scheduled for publication until you have made the required changes, so a swift response is appreciated.

IMPORTANT: The editorial review process is now complete. PLOS will only permit corrections to spelling, formatting or significant scientific errors from this point onwards. Requests for major changes, or any which affect the scientific understanding of your work, will cause delays to the publication date of your manuscript.

If your institution or institutions have a press office, please notify them about your upcoming paper to help maximize its impact. If they'll be preparing press materials, please inform our press team as soon as possible -- no later than 48 hours after receiving the formal acceptance. Your manuscript will remain under strict press embargo until 2 pm Eastern Time on the date of publication. For more information, please contact globalpubhealth@plos.org.

Thank you again for supporting Open Access publishing; we are looking forward to publishing your work in PLOS Global Public Health.

Best regards,

Julia Robinson

Executive Editor

PLOS Global Public Health

***********************************************************

Reviewer Comments (if any, and for reference):

Reviewer's Responses to Questions

**Comments to the Author**

Reviewer #2: All comments have been addressed

**********

publication criteria?

Reviewer #2: Yes

**********

3. Has the statistical analysis been performed appropriately and rigorously?

Reviewer #2: N/A

**********

4. Have the authors made all data underlying the findings in their manuscript fully available (please refer to the Data Availability Statement at the start of the manuscript PDF file)?

The PLOS Data policy

Reviewer #2: Yes

**********

5. Is the manuscript presented in an intelligible fashion and written in standard English?

Reviewer #2: Yes

**********

Reviewer #2: I thank the authors for carefully revising the manuscript and providing detailed responses to the reviewers' comments. The revised version has improved considerably in terms of methodological transparency, clarity of reporting, and overall presentation. In particular, the additional descriptions of participant recruitment, language translation procedures, confidentiality safeguards during transcription and translation, and the justification of the analytical approach have strengthened the manuscript. Subject to minor editorial proofreading to correct a few typographical and formatting inconsistencies, I consider the manuscript suitable for publication.

**********

what does this mean?). If published, this will include your full peer review and any attached files.

**Do you want your identity to be public for this peer review?**  If you choose “no”, your identity will remain anonymous but your review may still be made public.

For information about this choice, including consent withdrawal, please see our Privacy Policy

Reviewer #2: **Yes:**  Adekogbe Adebola Sunday

**********
